# Pyridine Regioisomerism and Aryl Substitution Govern the Divergent Cyclization of Isoniazid-Derived Thiosemicarbazides: Mechanistic DFT Analysis and Antimycobacterial Evaluation

**DOI:** 10.3390/ijms27219477

**Published:** 2026-10-23

**Authors:** Agata Paneth, Nazar Trotsko, Agnieszka Głogowska, Ewa Augustynowicz-Kopeć, Piotr Paneth

**Affiliations:** 1 Department of Organic Chemistry, Medical University of Lublin, Chodzki 4A, 20-093 Lublin, Poland; agata.paneth@umlub.edu.pl; 2 Department of Microbiology, National Tuberculosis and Lung Diseases Research Institute, Plocka 26, 01-138 Warsaw, Poland; a.glogowska@igichp.edu.pl (A.G.); e.kopec@igichp.edu.pl (E.A.-K.); 3 Institute of Applied Radiation Chemistry, Faculty of Chemistry, Lodz University of Technology, Zeromskiego 116, 90-924 Lodz, Poland

**Keywords:** isoniazid, thiosemicarbazides, pyridine regioisomerism, thiazolidin-4-ones, 1,3,4-oxadiazoles, cyclization, DFT, reaction mechanism, *Mycobacterium tuberculosis*

## Abstract

Isoniazid is an established antitubercular pharmacophore widely used as a structural platform for developing compounds with potential activity against *Mycobacterium tuberculosis*. This study investigated isoniazid-derived thiosemicarbazides as precursors for the synthesis of thiazolidin-4-one derivatives, focusing on the influence of pyridine regioisomerism and aryl substitution on cyclization outcomes. Thiosemicarbazides containing 2-, 3-, or 4-pyridyl moieties and fluoro- or chloro-substituted phenyl groups were synthesized and subjected to cyclization with ethyl bromoacetate. Density functional theory (DFT) calculations were performed to compare the competing pathways leading to thiazolidin-4-one and 1,3,4-oxadiazole formation, and the resulting heterocyclic compounds were evaluated against four *M. tuberculosis* strains. An initial control experiment using a 2-pyridyl/2-fluorophenyl thiosemicarbazide yielded the target thiazolidin-4-one as the sole product. However, investigation of the broader substrate series revealed pronounced substrate-dependent differences in cyclization selectivity. Most substrates afforded 1,3,4-oxadiazoles, whereas 2-pyridyl thiosemicarbazides bearing *ortho*-fluoro or *ortho*-chloro substituents selectively formed thiazolidin-4-ones. DFT calculations provided an energetic rationale for the observed divergence in cyclization pathways. Despite systematic structural diversification, none of the investigated 1,3,4-oxadiazole or thiazolidin-4-one derivatives, including ester-functionalized analogues, exhibited appreciable antimycobacterial activity (MIC ≥ 512 µg/mL). These findings demonstrate that pyridine regioisomerism combined with aryl substitution strongly influences the cyclization pathways of isoniazid-derived thiosemicarbazides. However, the pronounced structure–reactivity relationship governing cyclization was not accompanied by a meaningful structure–activity relationship against *Mycobacterium tuberculosis*. The combined experimental and computational results provide insight into the factors governing cyclization selectivity and inform the design of future INH-derived analogues.

## 1. Introduction

Tuberculosis (TB), caused primarily by *Mycobacterium tuberculosis* (Mtb), remains a major global health challenge despite the availability of effective antimicrobial therapy [1,2,3,4,5]. The emergence and continued spread of drug-resistant Mtb strains, particularly multidrug-resistant and rifampicin-resistant tuberculosis, further complicate treatment and underscore the need for new chemical entities with antimycobacterial activity [1,2,3,4,5,6,7,8,9,10,11]. The identification of structurally diverse antimycobacterial agents and the elucidation of structural factors governing their chemical and biological properties therefore remain important objectives in medicinal chemistry [8,9,10,11,12].

Isoniazid (INH) is a key component of first-line antitubercular therapy [1,5,13,14]. Structural modification of the INH scaffold has been widely explored as an approach to the development of new antimycobacterial agents, including INH derivatives and hybrid compounds with diverse biological activities [13,14,15,16,17,18,19,20,21,22]. Such structural modifications can substantially alter the physicochemical, chemical, and biological properties of the resulting compounds. In particular, structural modification of the pyridine-containing moiety of INH-derived molecules provides an opportunity to investigate how relatively small changes in molecular architecture affect both chemical reactivity and biological behaviour.

Thiosemicarbazides are versatile synthetic intermediates that can be converted into a broad range of biologically relevant heterocyclic scaffolds [23,24,25,26,27,28]. In particular, their reactions with α-halo carbonyl compounds provide a convenient route to thiazolidin-4-one derivatives through cyclization [22,23,26,29,30,31,32]. Thiazolidin-4-ones represent an important class of heterocyclic scaffolds in medicinal chemistry and exhibit a broad spectrum of biological activities [33]. Considerable attention has been devoted to their antitubercular potential, with numerous thiazolidin-4-one derivatives demonstrating activity against Mtb [13,14,20,21,22,34,35]. The synthesis of thiazolidin-4-one derivatives from INH-derived thiosemicarbazide precursors therefore represents a rational strategy for expanding the chemical space of INH-derived compounds.

Importantly, the reaction of thiosemicarbazides with α-haloacetates is not necessarily restricted to the formation of thiazolidin-4-ones, as the reaction pathway may depend strongly on the structure of the precursor and the reaction conditions, with alternative cyclization pathways leading to other heterocyclic scaffolds, including 1,3,4-oxadiazoles [23,26,29,30,36,37,38,39]. Recent mechanistic studies have further shown that the formation of thiazolidin-4-ones and 1,3,4-oxadiazoles can be mechanistically interconnected, with carbodiimide intermediates implicated in the conversion of thiazolidin-4-ones into oxadiazoles [40]. The factors determining which pathway predominates are therefore of interest not only for synthetic control but also for understanding how relatively small structural modifications can alter reaction selectivity.

Against this background, the present study was designed to investigate how pyridine regioisomerism and aryl substitution affect the cyclization behaviour of INH-derived thiosemicarbazides upon reaction with ethyl bromoacetate. A series of thiosemicarbazides incorporating 2-, 3-, and 4-pyridyl moieties and fluoro- or chloro-substituted phenyl groups was prepared, with systematic variation in the halogen position. These thiosemicarbazides were subsequently subjected to cyclization with ethyl bromoacetate, with the initial aim of obtaining a structurally diversified series of thiazolidin-4-one derivatives for subsequent antimycobacterial evaluation.

Because structurally related thiosemicarbazides have also been reported to undergo 1,3,4-oxadiazole formation, an initial control experiment was performed with a representative 2-pyridyl/2-fluorophenyl thiosemicarbazide to determine whether the selected reaction conditions would afford the intended thiazolidin-4-one scaffold. This experiment afforded the expected thiazolidin-4-one as the sole isolated product, confirming that the targeted cyclization pathway was accessible under the selected conditions. Extension of the reaction to the complete substrate series, however, revealed an unexpected divergence in product formation, with the cyclization outcome depending on both the pyridine regioisomerism and the aryl substitution pattern.

This substrate-dependent divergence provided an opportunity to investigate the structural determinants governing the competition between thiazolidin-4-one and 1,3,4-oxadiazole formation. To elucidate the molecular basis of the observed selectivity, density functional theory (DFT) calculations were subsequently performed to compare the competing reaction pathways and evaluate the energetic factors associated with the experimentally observed product distribution.

The resulting heterocyclic compounds were also evaluated for their antimycobacterial activity against *M. tuberculosis*. The biological evaluation included both the originally targeted thiazolidin-4-one derivatives and the predominant oxadiazole products obtained during the cyclization study. In addition, a second series of ester-functionalized thiazolidin-4-one derivatives was designed and synthesized via an alternative synthetic route to determine whether this structural modification could enhance their antimycobacterial activity.

Accordingly, the present study addresses two closely related but distinct questions: how molecular structure controls the cyclization of INH-derived thiosemicarbazides and whether this structure-dependent chemical behaviour translates into differences in antimycobacterial activity. By combining systematic synthesis, structural characterization, DFT analysis, and biological evaluation, the study provides insight into the relationship between structure–reactivity and structure–activity in this class of INH-derived compounds.

## 2. Results

### 2.1. Study Design, Optimization, and Cyclization Product Distribution

The present study was initiated with a systematic investigation of the cyclization behaviour of the INH-derived thiosemicarbazides described above, with particular emphasis on the formation of thiazolidin-4-one derivatives. The substrate series was designed to assess the effects of pyridine regioisomerism and phenyl-ring substitution on the outcome of the cyclization with ethyl bromoacetate. Accordingly, thiosemicarbazides incorporating 2-, 3-, and 4-pyridyl moieties and fluoro- or chloro-substituted phenyl groups were selected for investigation. For clarity, the thiosemicarbazides were designated according to the notation **1-(Py/Ar)**, where **1** identifies the thiosemicarbazide series, **Py** denotes the pyridine regioisomer (2-Py, 3-Py, or 4-Py), and **Ar** denotes the halogen-substituted phenyl group (*o*-F, *m*-F, *p*-F, *o*-Cl, *m*-Cl, or *p*-Cl). Thus, for example, **1-(2-Py/*o*-Cl)** denotes the thiosemicarbazide bearing a 2-pyridyl moiety and an *ortho*-chlorophenyl group.

**Figure 1:**
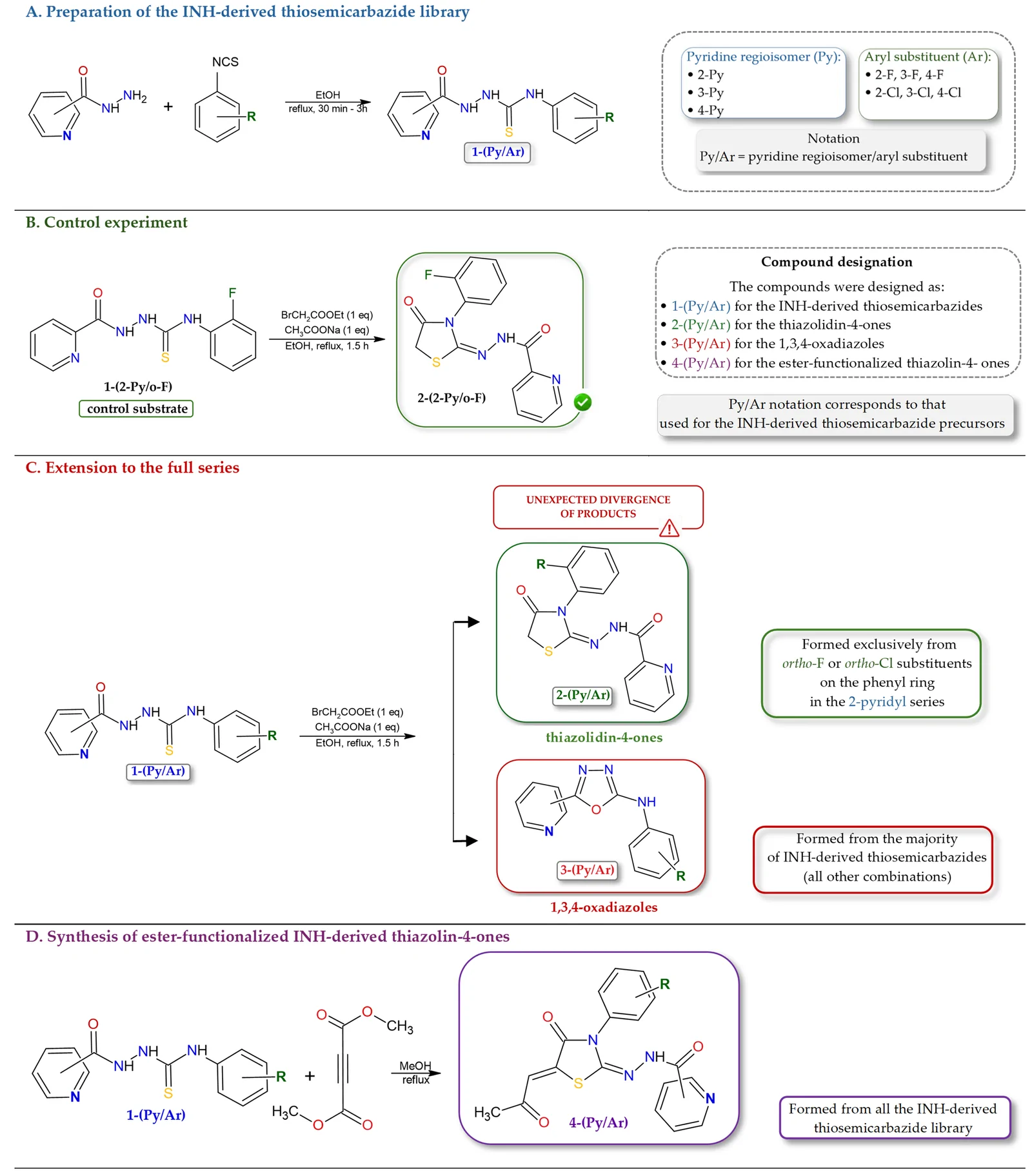
Synthetic strategy and divergent cyclization pathways of INH-derived thiosemicarbazides. For compound structures and isolated yields, see Table 1.

Given the possibility of competing cyclization pathways, the initial experiments were designed to establish whether the selected reaction conditions were suitable for the formation of the intended thiazolidin-4-one scaffold. A representative 2-pyridyl thiosemicarbazide bearing a 2-fluorophenyl substituent, **1-(2-Py/*o*-F)**, was therefore selected as the control substrate (Figure 1).

**Table 1:** Structures of the INH-derived thiazolidin-4-ones and 1,3,4-oxadiazoles, together with the corresponding isolated yields.

Compound	Structure of Cyclization Products	Isolated Yield (%)
**1-(2-Py/*o*-Cl)**	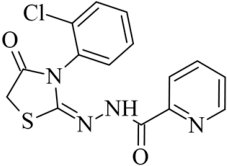	70
**1-(2-Py/*m*-Cl)**	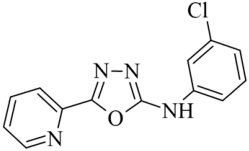	57
**1-(2-Py/*p*-Cl)**	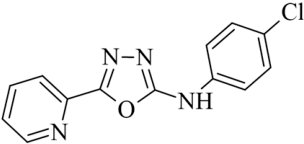	62
**1-(2-Py/*o*-F)**	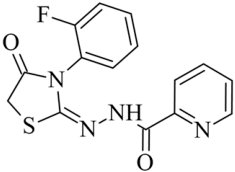	66
**1-(2-Py/*m*-F)**	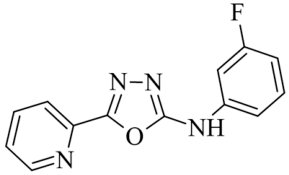	47
**1-(2-Py/*p*-F)**	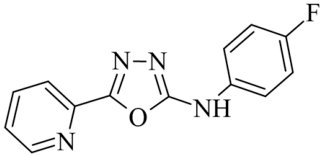	64
**1-(3-Py/*o*-Cl)**	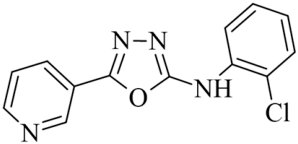	48
**1-(3-Py/*m*-Cl)**	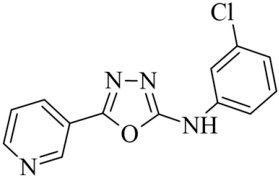	73
**1-(3-Py/*p*-Cl)**	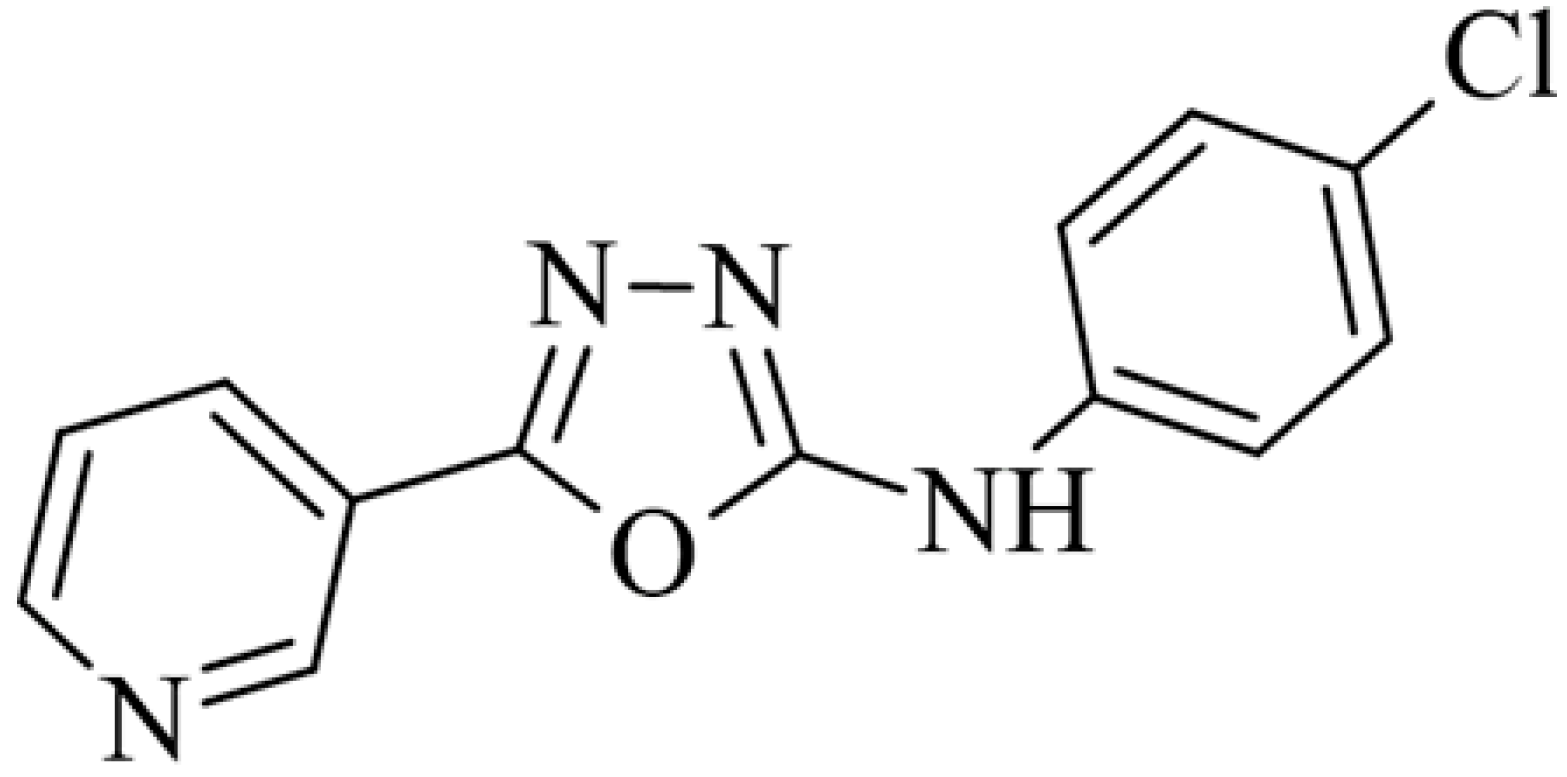	54
**1-(3-Py/*o*-F)**	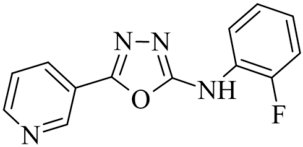	48
**1-(3-Py/*m*-F)**	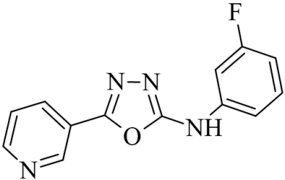	70
**1-(3-Py/*p*-F)**	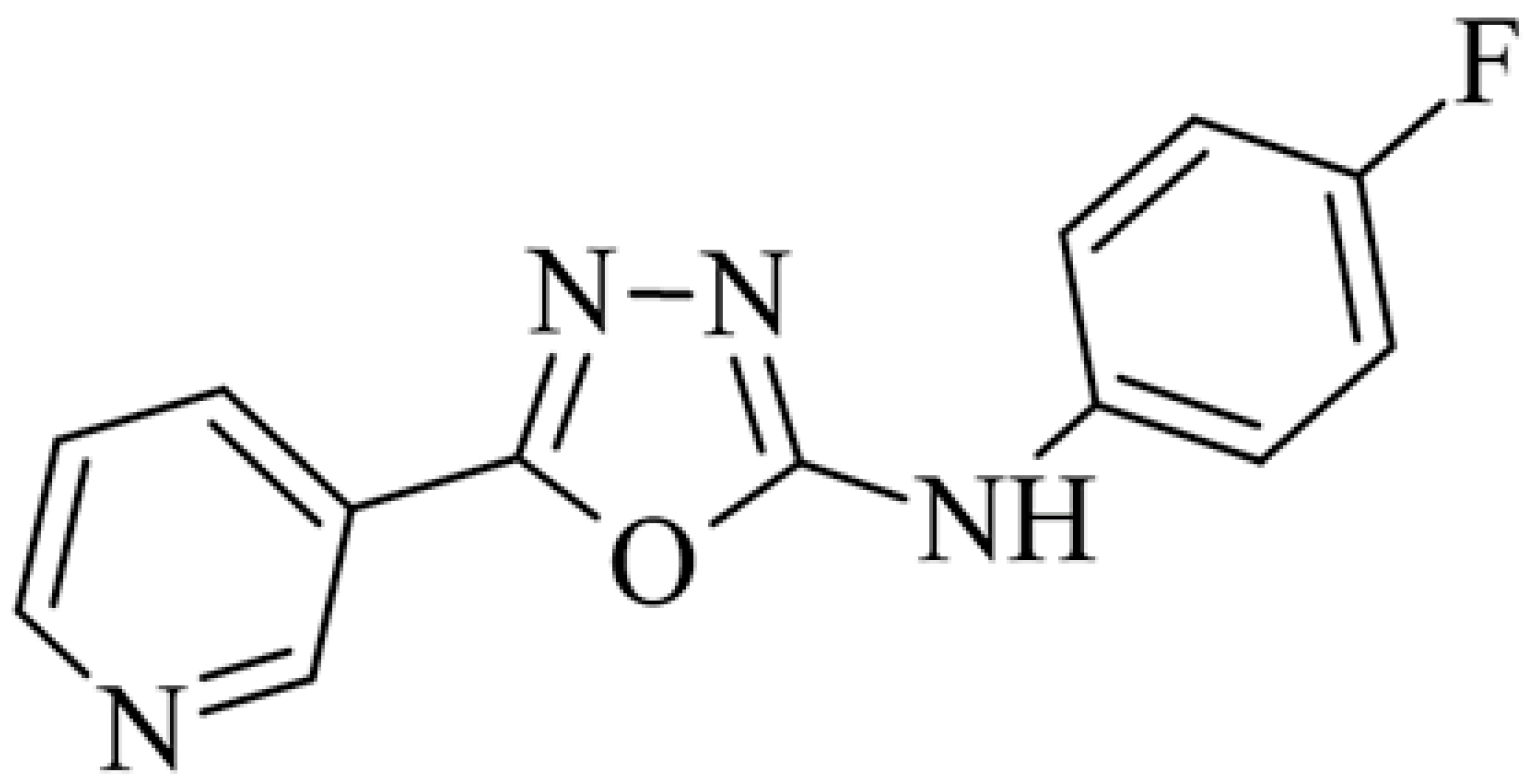	73
**1-(4-Py/*o*-Cl)**	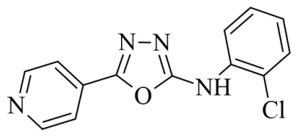	51
**1-(4-Py/*m*-Cl)**	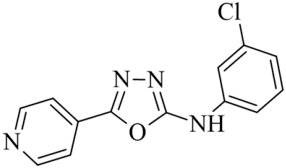	49
**1-(4-Py/*p*-Cl)**	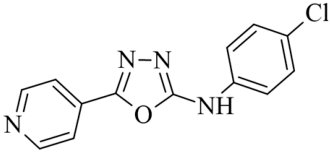	47
**1-(4-Py/*o*-F)**	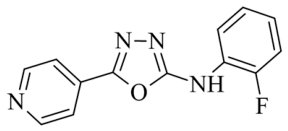	79
**1-(4-Py/*m*-F)**	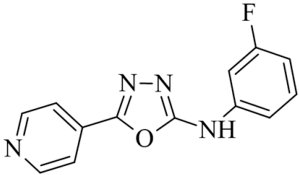	71
**1-(4-Py/*p*-F)**	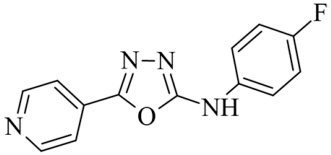	68

The cyclization of the control substrate with ethyl bromoacetate was first optimized with respect to the base, solvent, and temperature. Among the bases examined, anhydrous sodium acetate provided the most favourable outcome, whereas NaOH and K_2_CO_3_ were less effective. The reaction outcome was also strongly influenced by the solvent and temperature. Little or no product formation was observed at room temperature in water, dimethylformamide, ethanol, or acetone, whereas heating in ethanol promoted efficient cyclization. The optimized conditions therefore consisted of equimolar amounts of thiosemicarbazide and ethyl bromoacetate in the presence of anhydrous sodium acetate in absolute ethanol under reflux. A detailed summary of the optimization experiments, including the reaction conditions and corresponding isolated yields, is provided in Table S1 (Supporting Information).

Under these conditions, the selected 2-pyridyl/2-fluorophenyl thiosemicarbazide **1-(2-Py/*o*-F)** afforded the expected thiazolidin-4-one derivative, **2-(2-Py/*o*-F)**, as the sole isolated product in 66% yield. The structure of **2-(2-Py/*o*-F)** was confirmed by ^1^H and ^13^C NMR. The ^1^H NMR spectrum showed the characteristic methylene resonance of the newly formed thiazolidin-4-one ring at δ 4.29 ppm, while the hydrazide NH proton appeared as a downfield singlet at δ 11.53 ppm. The ^13^C NMR spectrum displayed the expected carbonyl resonances together with the characteristic signal of the endocyclic methylene carbon.

Following optimization, the complete substrate series was prepared from the corresponding 2-, 3-, and 4-pyridinecarboxylic acid hydrazides and appropriately substituted phenyl isothiocyanates. Fluoro and chloro substituents were introduced at the *ortho*, *meta*, and *para* positions of the phenyl ring, generating a systematically organized set of 18 thiosemicarbazide precursors in which pyridine regioisomerism and aryl substitution could be varied independently. The corresponding thiosemicarbazides were obtained in high yields of 90–97%, according to previously reported procedures [41,42].

For clarity, the products obtained in the first-generation cyclization were designated as **2-(Py/Ar)** for thiazolidin-4-ones and **3-(Py/Ar)** for 1,3,4-oxadiazoles, with the Py/Ar notation corresponding to that used for the thiosemicarbazide precursors. The second series of thiazolidin-4-one derivatives, incorporating an additional ester functionality, was designated using the notation **4-(Py/Ar)**, where the Py/Ar notation corresponds to that used for the thiosemicarbazide precursors.

Application of the optimized conditions to the complete substrate series revealed a clear divergence in the reaction outcome. As shown in Figure 1, rather than proceeding uniformly toward the targeted thiazolidin-4-one scaffold, the reaction pathway depended on the substitution pattern of the thiosemicarbazide precursor. Most substrates afforded the corresponding 1,3,4-oxadiazoles as the sole isolated products, with yields ranging from 47 to 79%. This outcome was observed consistently among the 3-pyridyl and 4-pyridyl derivatives, whereas the corresponding oxadiazoles were also obtained as the sole isolated products from most of the 2-pyridyl derivatives. The exceptions within the 2-pyridyl series were specifically associated with the presence of an *ortho*-halogen substituent on the phenyl ring. Both **1-(2-Py/*o*-F)** and **1-(2-Py/*o*-Cl)** afforded the corresponding thiazolidin-4-ones, **2-(2-Py/*o*-F)** and **2-(2-Py/*o*-Cl)**, respectively, as the sole isolated products, in yields of 66–70%. In contrast, relocation of the same fluoro or chloro substituent from the *ortho* position to the *meta* or *para* position resulted in formation of the corresponding 1,3,4-oxadiazoles. Thus, within the investigated series, thiazolidin-4-one formation was observed exclusively for substrates combining a 2-pyridyl moiety with an *ortho*-halogen-substituted phenyl group.

The similar behaviour of the *ortho*-fluoro and *ortho*-chloro substrates was particularly noteworthy. Despite differences in the size and electronic properties of fluorine and chlorine, both substituents favoured thiazolidin-4-one formation only when combined with the 2-pyridyl moiety and positioned at the *ortho* position of the phenyl ring. Thus, neither the pyridine regioisomerism nor the aryl halogen substitution pattern alone appears to account for the observed selectivity; rather, the cyclization outcome seems to depend on their specific structural combination within the thiosemicarbazide precursor. This finding suggests that the overall molecular arrangement, including the relative positioning of the pyridyl and *ortho*-halogen-substituted phenyl groups, plays a more important role in determining the preferred cyclization pathway than the identity of the halogen alone.

The structures of the isolated products were confirmed by spectroscopic analysis. The thiazolidin-4-one derivatives showed the characteristic NMR features of the newly formed sulfur-containing five-membered ring, including the methylene resonance and the carbonyl signals observed in the ^13^C NMR spectra. In contrast, the 1,3,4-oxadiazole derivatives lacked the methylene resonance and the carbonyl signals characteristic of the thiazolidin-4-one ring, and their spectroscopic data were consistent with the formation of the more highly unsaturated 1,3,4-oxadiazole scaffold.

Overall, the experimental results established that although thiazolidin-4-one formation was readily accessible under the optimized reaction conditions, the cyclization outcome was strongly substrate-dependent. Systematic variation in pyridine regioisomerism and aryl substitution revealed a distinct structural pattern associated with preferential formation of either thiazolidin-4-ones or 1,3,4-oxadiazoles. To further elucidate the molecular basis of this substrate-dependent selectivity, DFT calculations were subsequently performed to compare the competing cyclization pathways.

### 2.2. DFT Investigation of the Cyclization Pathways

To elucidate the origin of the substrate-dependent cyclization selectivity, DFT calculations were performed for the representative ester-functionalized thiosemicarbazide derivatives **1-(2-Py/*o*-Cl)** and **1-(3-Py/*o*-Cl)**, which show contrasting preferences for formation of thiazolidin-4-one and 1,3,4-oxadiazole products, respectively. The calculations compared the energetic requirements of the competing pathways, with particular attention to proton transfer, ring closure, and the conformational reorganization required for oxadiazole formation.

#### 2.2.1. Computational Model and Conformational Analysis

**Figure 2:**
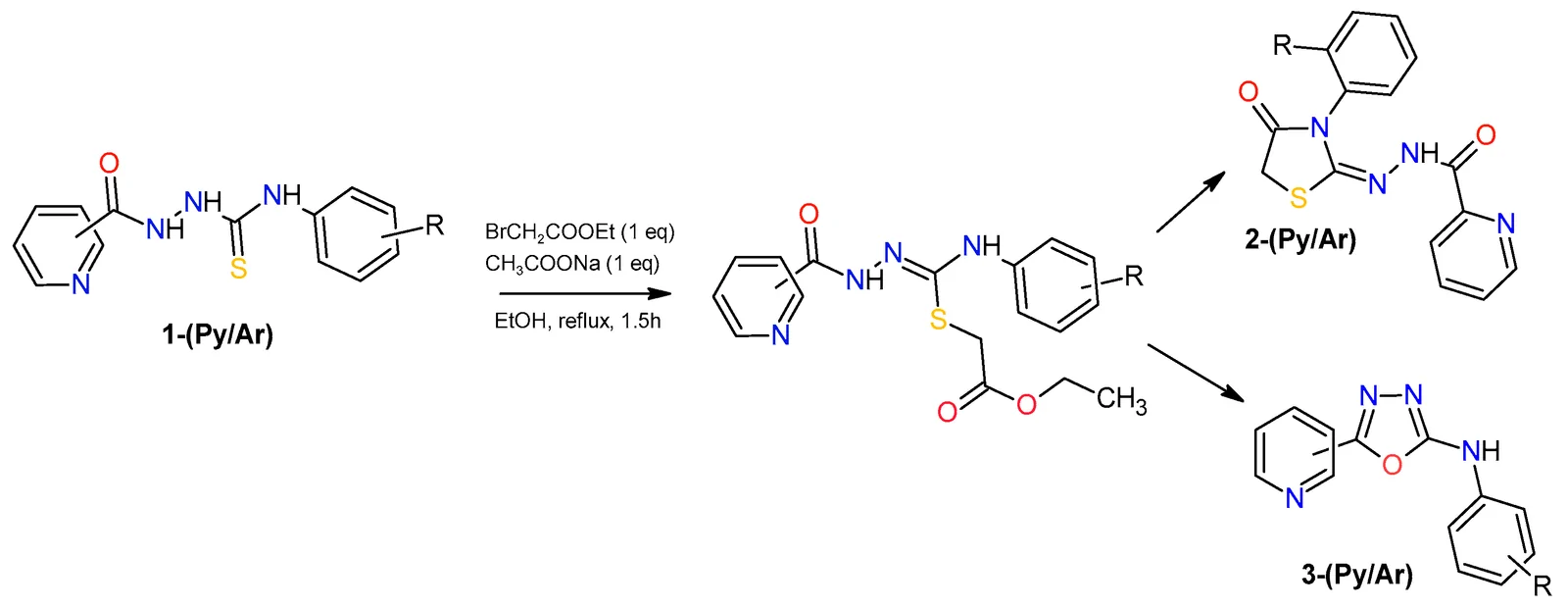
Synthetic strategy of ester-functionalized INH-derived thiosemicarbazides.

The initial structures of ester-functionalized **1-(2-Py/*o*-Cl)** and **1-(3-Py/*o*-Cl)** (Figure 2) were subjected to conformational analysis using the OPLS force field. The lowest-energy conformers identified in this analysis were subsequently optimized at the DFT level using a continuum solvent model for ethanol. The same level of theory was employed for all subsequent calculations.

**Figure 3:**
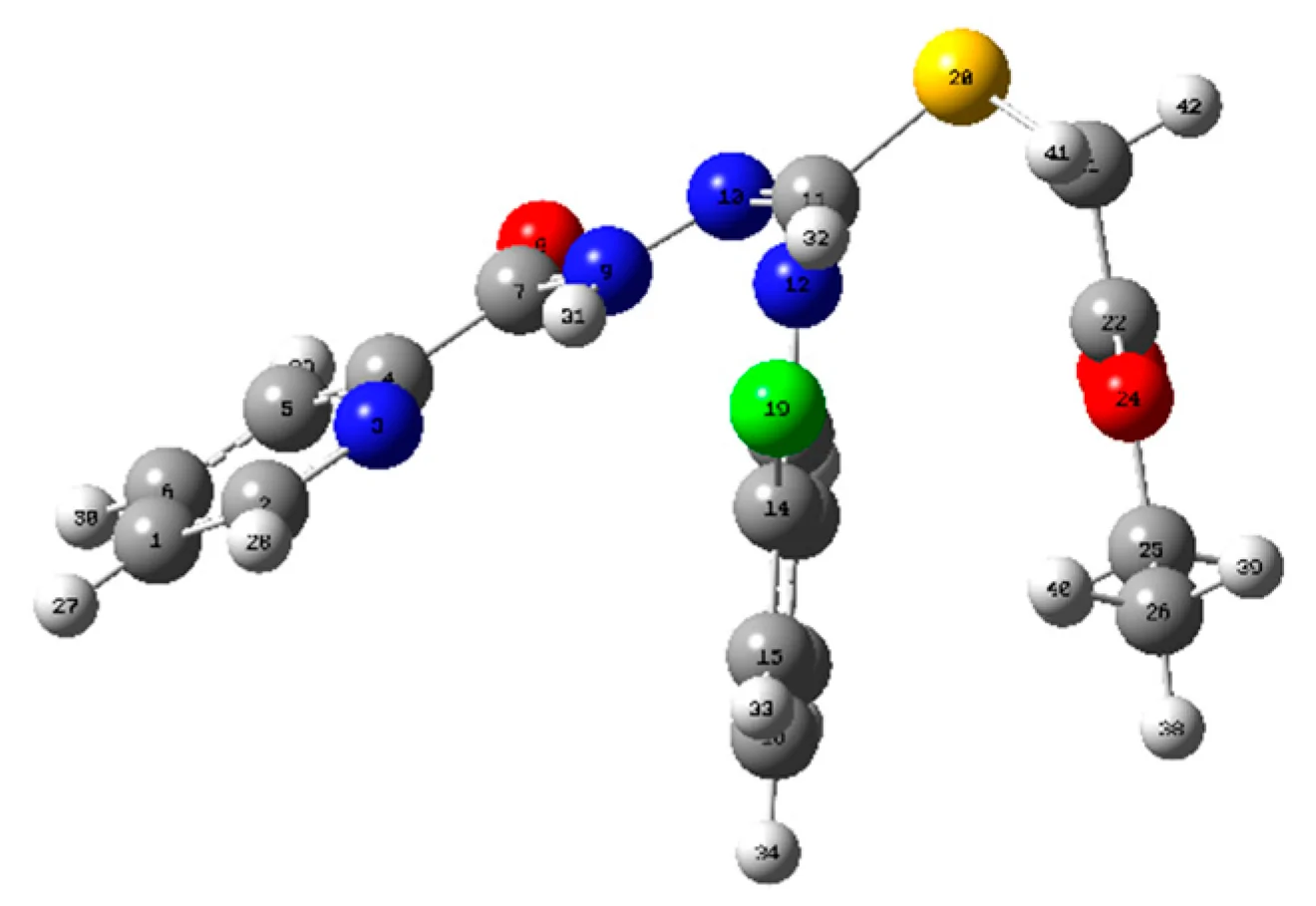
Most stable conformer obtained from molecular mechanics and subsequently optimized at the DFT level, exemplified for ester-functionalized **1-(2-Py/*o*-Cl)**. The atom numbering used in the discussion is indicated. Carbon atoms are rendered in grey, hydrogens in white, oxygen in red, sulfur in yellow and chlorine in green.

The optimized neutral structures of ester-functionalized **1-(2-Py/*o*-Cl)** and **1-(3-Py/*o*-Cl)** were closely similar in terms of their overall geometry. In both compounds, the chlorophenyl moiety and the ester-containing side chain adopted approximately parallel orientations (Figure 3). The atom numbering used throughout the subsequent mechanistic analysis is also shown in this figure.

#### 2.2.2. DFT Analysis of Thiazolidin-4-One Formation

Formation of the thiazolidin-4-one ring was first examined by a relaxed scan of the N12-C22 distance, which was reduced from approximately 3.1 Å in the reactant to 1.4 Å, corresponding to formation of an N-C single bond. The highest-energy structures along the resulting profiles were used as starting geometries for transition-state searches.

**Figure 4:**
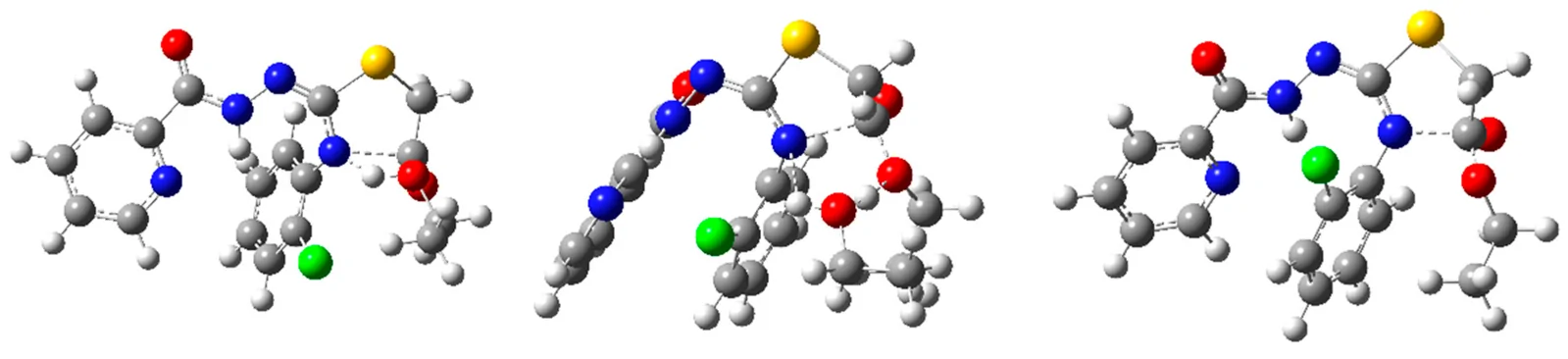
Transition-state structures for thiazolidin-4-one formation under uncatalyzed (**left**), solvent-assisted (**middle**), and acetate-catalyzed (**right**) conditions, exemplified for ester-functionalized **1-(2-Py/*o*-Cl)**. Dotted lines indicate bonds being formed or broken.

Direct cyclization gave Gibbs free-energy barriers of 48.3 kcal/mol for ester-functionalized **1-(2-Py/*o*-Cl)** and 47.5 kcal/mol for ester-functionalized **1-(3-Py/*o*-Cl)** (Figure 4, left), making this pathway energetically inaccessible under the experimental conditions. Inclusion of one explicit ethanol molecule lowered the barrier for ester-functionalized **1-(2-Py/*o*-Cl)** to 43.4 kcal/mol, with a comparable decrease for ester-functionalized **1-(3-Py/*o*-Cl)** (Figure 4, middle), but the barriers remained above 40 kcal/mol.

Acetate-assisted catalysis was therefore examined. Acetate abstracts the proton from N12, changing the mechanism from a concerted process to a two-step pathway. This proton-transfer step has only a very small barrier on the potential-energy surface and is effectively barrierless on the Gibbs free-energy surface [43,44]. The subsequent ring closure proceeds through a distinct transition state (Figure 4, right) with Gibbs free-energy barriers of 12.1 kcal/mol for ester-functionalized **1-(2-Py/*o*-Cl)** and 11.1 kcal/mol for ester-functionalized **1-(3-Py/*o*-Cl)**. Departure of the enolate species and its protonation by acetic acid introduce no additional significant barrier, giving an energetically accessible acetate-assisted pathway in both systems.

#### 2.2.3. DFT Analysis of 1,3,4-Oxadiazole Formation

The pathway to the 1,3,4-oxadiazole ring introduced an additional conformational requirement. In the neutral ester-functionalized **1-(2-Py/*o*-Cl)** and **1-(3-Py/*o*-Cl)** structures, the C(=O)-N(H)-N=C scaffold preferentially adopted a trans arrangement, with O8 and C11 on opposite sides of the N9-N10 bond (Figure 3), whereas ring closure requires the corresponding cis geometry.

**Figure 5:**
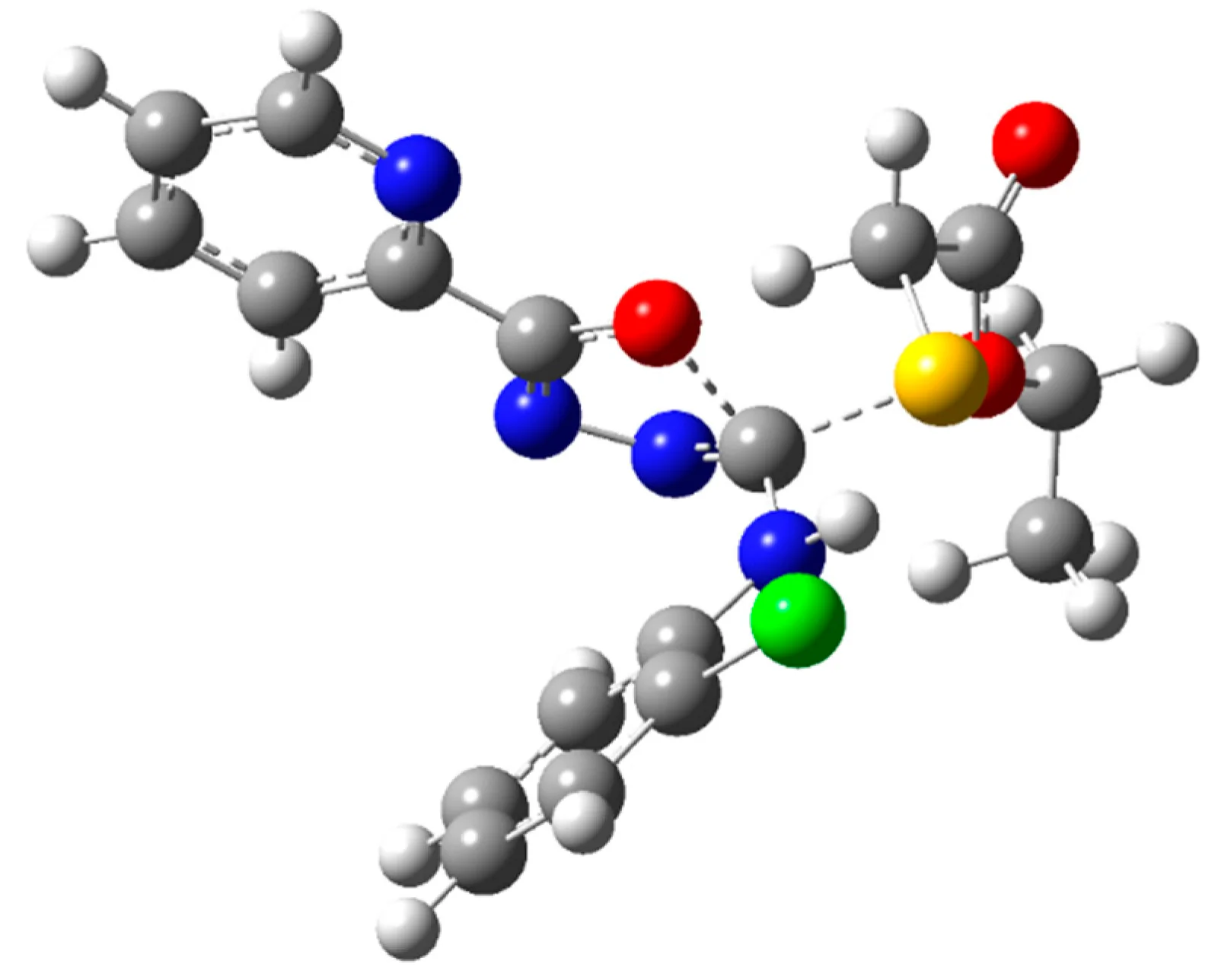
Transition-state structure corresponding to cyclization toward the 1,3,4-oxadiazole ring.

Attempts to identify direct pathways from the N- or O-protonated forms to a cyclization-competent geometry were unsuccessful. The analysis therefore focused on the N9-deprotonated species generated by acetate-assisted proton transfer. Deprotonation proceeded without an appreciable barrier and produced an anion with the negative charge predominantly localized at N9. Subsequent ring closure occurred through the transition state shown in Figure 5, with Gibbs free-energy barriers of 29.7 and 30.8 kcal/mol for ester-functionalized **1-(2-Py/*o*-Cl)** and **1-(3-Py/*o*-Cl)**, respectively.

#### 2.2.4. Conformational Rotation Preceding Oxadiazole Cyclization

Because the ring-closing barriers themselves differed little between ester-functionalized **1-(2-Py/*o*-Cl)** and **1-(3-Py/*o*-Cl)**, the conformational rearrangement preceding oxadiazole formation was examined separately. A relaxed scan of the C7-N9-N10-C11 torsional angle was performed for the N9-deprotonated reactants, following rotation from the preferred trans geometry toward the cis arrangement required for cyclization. Deprotonation reversed the relative conformational stability, making the cis conformer more stable than the trans conformer in both systems.

The trans-to-cis rotational barrier was 8.8 kcal/mol for ester-functionalized **1-(2-Py/*o*-Cl)** but only 3.1 kcal/mol for ester-functionalized **1-(3-Py/*o*-Cl)**; the corresponding reaction free energies were −2.8 and −3.1 kcal/mol, respectively (Table 2). Thus, in contrast to the nearly substrate-independent ring-closing barriers, the conformational step showed a pronounced energetic difference between the two substrates.

### 2.3. Synthesis of Selected Ester-Functionalized Thiazolidin-4-One Derivatives

Because thiazolidin-4-one formation remained the original synthetic objective, the study was further extended to a second, independently prepared series of ester-functionalized thiazolidin-4-one derivatives **4-(Py/Ar)**. Eight selected thiosemicarbazide precursors were used to prepare the corresponding derivatives, which retained the same core structural variables investigated in the initial study, namely pyridine regioisomerism and the fluoro- or chloro-substitution pattern of the aryl ring, while incorporating an additional ester functionality into the thiazolidin-4-one scaffold. This structural modification was intended to provide insight into whether the introduction of ester functionality could influence the antimycobacterial properties of the resulting compounds.

**Figure 6:**
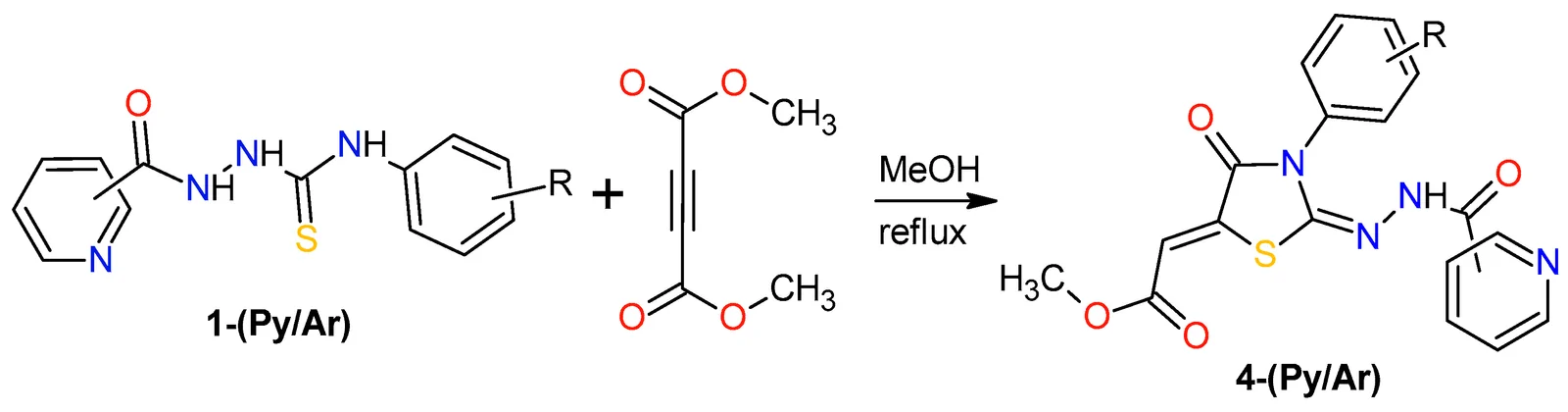
Synthesis of the C5-ester-functionalized thiazolidin-4-one derivatives.

The ester-functionalized thiazolidin-4-one derivatives **4-(Py/Ar)** were obtained through an alternative synthetic route involving a one-pot reaction of the corresponding thiosemicarbazide precursors with dimethyl acetylenedicarboxylate (DMAD) in methanol under reflux [45], as shown in Figure 6. This approach afforded the corresponding C5-ester-functionalized thiazolidin-4-one derivatives in excellent yields.

The molecular structures of the synthesized compounds were confirmed by ^1^H and ^13^C NMR spectroscopy. The ^1^H NMR spectra displayed the characteristic resonances expected for the proposed C5-ester-functionalized thiazolidin-4-one derivatives. The methyl protons of the ester group appeared as a singlet at δ 3.81 ppm, whereas the vinylic proton of the exocyclic C=C bond at the C-5 position of the thiazolidin-4-one ring was observed as a characteristic downfield singlet at δ 6.87 ppm. The downfield position of this signal is consistent with the conjugated environment of the vinylic proton within the exocyclic double bond and the adjacent electron-withdrawing ester group. The NH proton of the hydrazide moiety appeared as a downfield singlet at δ 11.34–11.82 ppm. The aromatic protons resonated within the expected region at δ 7.04–8.97 ppm.

The ^13^C NMR spectra provided further support for the proposed structures, showing the expected resonances of the ester, thiazolidin-4-one, and hydrazide carbonyl groups at δ 163.0–164.0, 165.3–166.5, and 160.5–162.3 ppm, respectively. The spectra also displayed the characteristic resonances of the two sp^2^ carbons involved in the exocyclic C=C bond at the C-5 position of the thiazolidin-4-one ring, with the corresponding signals observed at δ 115.7–115.9 and 138.5–141.4 ppm.

The successful synthesis of these derivatives enabled their subsequent evaluation to investigate the potential influence of ester functionality on antimycobacterial activity.

### 2.4. Antimycobacterial Activity

The antimycobacterial activity of the synthesized compounds was evaluated against *M. tuberculosis*. The biological evaluation encompassed the predominant 1,3,4-oxadiazole products **3-(Py/Ar)** obtained during the initial cyclization study, the two thiazolidin-4-one derivatives **2-(Py/Ar)** formed under the same reaction conditions, and the extended series of ester-functionalized thiazolidin-4-ones **4-(Py/Ar)**.

The compounds were tested against four *M. tuberculosis* strains representing distinct susceptibility profiles: the reference strain *M. tuberculosis* H_37_Rv, the drug-susceptible clinical isolate *M. tuberculosis* 192, the multidrug-resistant strain 36/26 (MDR; isoniazid- and rifampicin-resistant), and the isoniazid-monoresistant strain *M. tuberculosis* 3715. Isoniazid and rifampicin were included as reference drugs. Minimum inhibitory concentrations (MICs) were determined using the standardized antimycobacterial assay described in the Experimental section.

None of the compounds from the initial cyclization study demonstrated appreciable antimycobacterial activity. The oxadiazole derivatives **3-(Py/Ar)** showed MIC values of ≥512 µg/mL against all four strains. Similarly, the two thiazolidin-4-one derivatives **2-(Py/Ar)** obtained from the 2-pyridyl substrates bearing *ortho*-fluoro or *ortho*-chloro substituents showed MIC values of ≥512 µg/mL against all tested strains. The extended series of ester-functionalized thiazolidin-4-one derivatives **4-(Py/Ar)** likewise failed to demonstrate meaningful antimycobacterial activity, with MIC values of ≥512 µg/mL across the tested compounds and strains. Thus, under the experimental conditions employed, neither the predominant oxadiazole products nor the thiazolidin-4-one derivatives, including the ester-functionalized series, showed meaningful inhibition of any of the investigated *M. tuberculosis* strains.

## 3. Discussion

### 3.1. Substrate-Dependent Divergence of the Cyclization Pathways

The experimental results show that closely related thiosemicarbazide precursors can follow different cyclization pathways under otherwise identical conditions. This divergence is particularly evident for the 2- and 3-pyridyl derivatives bearing an *ortho*-chloro substituent, indicating that relatively subtle structural differences influence the competition between thiazolidin-4-one and 1,3,4-oxadiazole formation.

Previous mechanistic studies of related systems have proposed initial *S*-alkylation followed by formation of a thiazolidinone-type intermediate, with subsequent transformation through carbodiimide-type intermediates providing a possible route to 1,3,4-oxadiazoles [40]. In the present study, this proposal provides a mechanistic framework rather than proof of a unique reaction sequence. The DFT calculations were used to identify which elementary steps distinguish **1-(2-Py/*o*-Cl)** from **1-(3-Py/*o*-Cl)** and could therefore contribute to the experimentally observed product divergence.

### 3.2. Mechanistic Interpretation Based on DFT Calculations

#### 3.2.1. Acetate-Assisted Formation of Thiazolidin-4-Ones

The calculations rule out direct thiazolidin-4-one ring closure as a viable pathway: the uncatalyzed barriers are approximately 48 kcal/mol, and explicit assistance by a single ethanol molecule still leaves barriers above 40 kcal/mol. Bulk solvent stabilization, even supplemented by one explicit ethanol molecule, therefore does not account for the observed reactivity.

**Figure 7:**
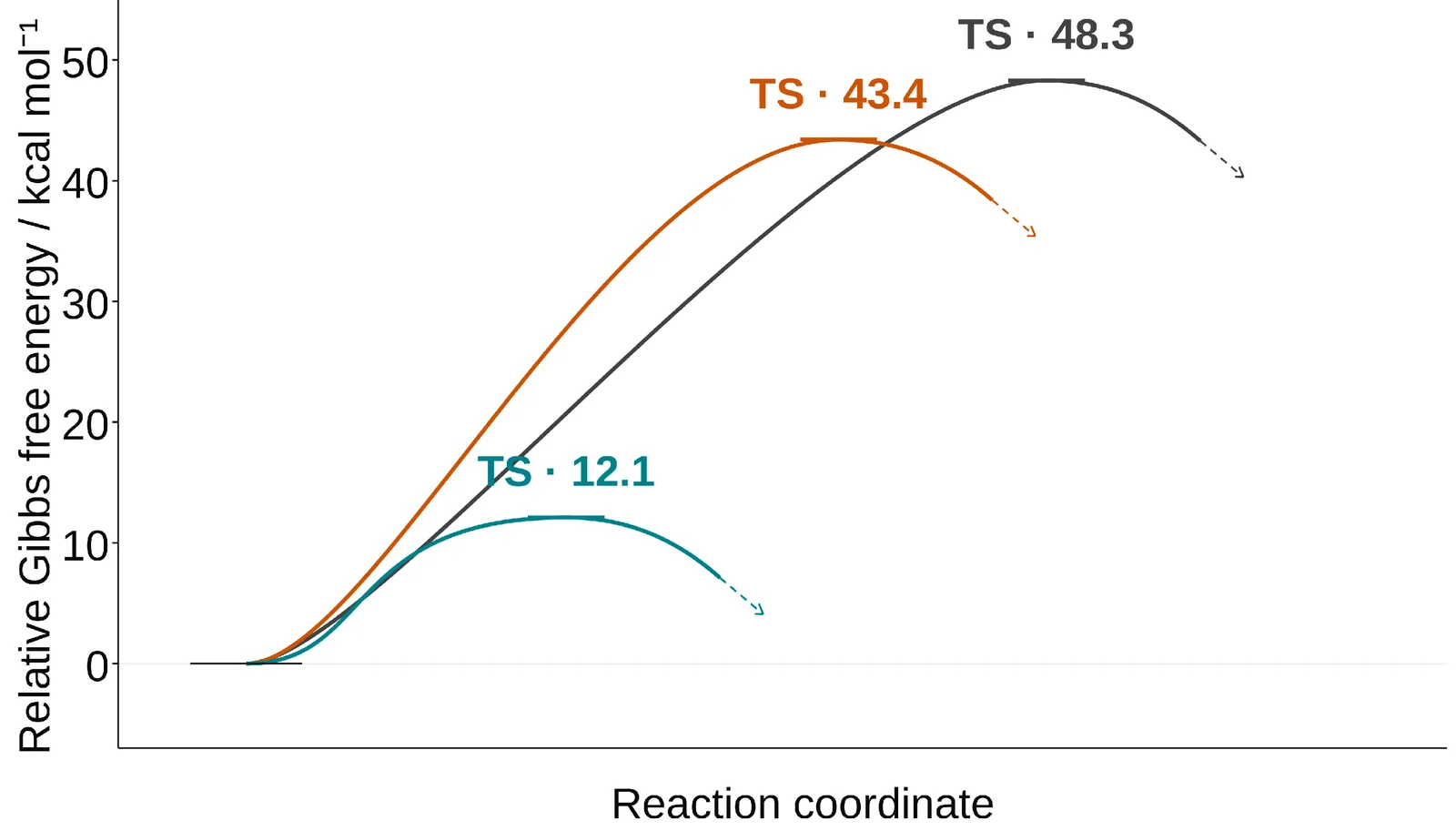
Comparison of the Gibbs free activation barriers for thiazolidin-4-one formation under uncatalyzed (blue), solvent-assisted (brown), and acetate-catalyzed (green) conditions, exemplified for ester-functionalized **1-(2-Py/*o*-Cl)**.

Acetate changes the mechanistic picture by facilitating proton transfer from N12 and converting ring closure into a stepwise process. The resulting cyclization barriers, 12.1 kcal/mol for ester-functionalized **1-(2-Py/*o*-Cl)** and 11.1 kcal/mol for ester-functionalized **1-(3-Py/*o*-Cl)**, are consistent with an accessible reaction under the experimental conditions. The comparison of the barriers for spontaneous and catalyzed cyclization is presented in Figure 7.

The key point for selectivity is that these two barriers differ by only about 1 kcal/mol. Acetate therefore appears to enable thiazolidin-4-one formation in both substrates, but the ring-closing step itself provides little energetic basis for their different product preferences.

#### 3.2.2. Energetic Requirements for 1,3,4-Oxadiazole Formation

The same conclusion applies to the final ring-closing step leading to the 1,3,4-oxadiazole: the calculated barriers for ester-functionalized **1-(2-Py/*o*-Cl)** and **1-(3-Py/*o*-Cl)** are 29.7 and 30.8 kcal/mol, respectively. Their similarity argues against the intrinsic cyclization transition state being the origin of substrate discrimination.

Oxadiazole formation differs, however, in requiring prior access to the cis arrangement of the C(=O)-N-N=C fragment, whereas the neutral precursors preferentially adopt the trans geometry. Conformational reorganization is therefore an additional prerequisite that is absent from a simple comparison of the final ring-closing transition states.

This shifts the mechanistic question from the intrinsic barrier of oxadiazole ring closure to the accessibility of the cyclization-competent conformation.

#### 3.2.3. Conformational Control of Product Selectivity

**Figure 8:**
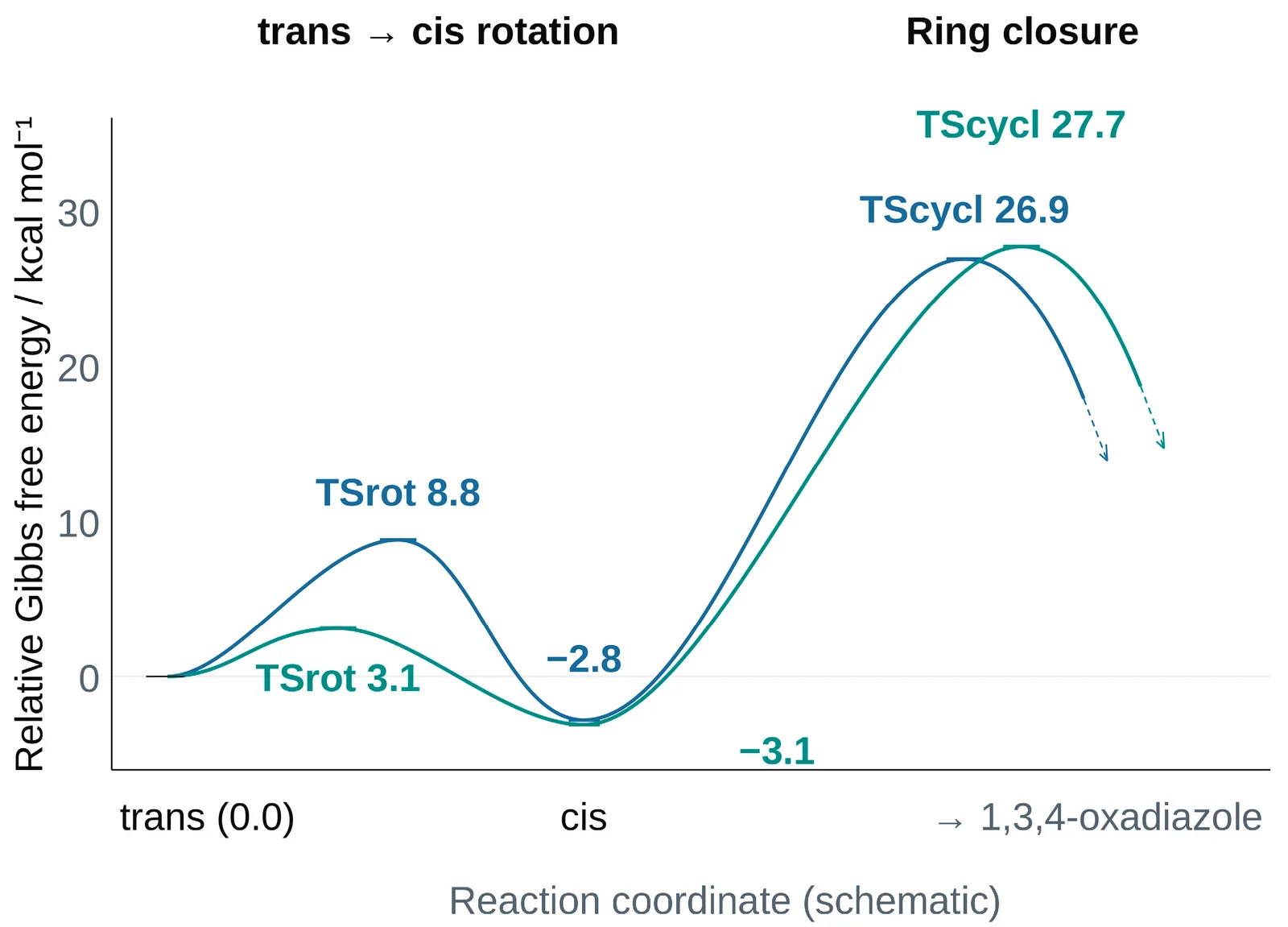
Two steps leading to cyclization to form 1,3,4-oxadiazole product for **1-(2-Py/*o*-Cl)** (blue) and **1-(3-Py/*o*-Cl)** (green).

The most pronounced substrate-dependent difference identified computationally occurs in the trans-to-cis rotation of the N9-deprotonated species. Although deprotonation makes the cis conformer thermodynamically favored in both systems, the barrier for reaching it depends strongly on the pyridine substitution pattern. These differences are illustrated in Figure 8.

For ester-functionalized **1-(2-Py/*o*-Cl)**, the calculated rotational barrier is 8.8 kcal/mol, compared with only 3.1 kcal/mol for ester-functionalized **1-(3-Py/*o*-Cl)**. The lower barrier for ester-functionalized **1-(3-Py/*o*-Cl)** is qualitatively consistent with the greater experimental propensity of the 3-pyridyl derivative to form the 1,3,4-oxadiazole product.

This conformational difference is therefore the clearest energetic feature that parallels the experimental selectivity. At the same time, it should not be interpreted as establishing the rotation as the rate-determining step: both rotational barriers are substantially lower than the subsequent cyclization barriers.

A more cautious mechanistic interpretation is that the pyridine substitution pattern modulates access to the oxadiazole-competent cis intermediate and thereby contributes to pathway selection. Once that geometry is reached, the intrinsic ring-closing energetics are similar for the two substrates.

#### 3.2.4. Limitations of the Computational Model

The calculated conformational trend provides a qualitative rationale for the observed substrate dependence. However, the continuum ethanol model may not fully capture specific solute–solvent interactions, particularly those involving proton transfer, charged intermediates, and the entropic contribution to the corresponding free-energy changes. The DFT results should therefore be regarded as mechanistic support for the proposed interpretation rather than a quantitative model of product selectivity.

### 3.3. Overall Mechanistic Picture

Taken together, the calculations suggest that acetate-assisted proton transfer enables cyclization, whereas differences in the intrinsic ring-closing barriers provide little explanation for the observed product preferences. Instead, conformational accessibility may contribute to the competition between the two pathways. This interpretation remains qualitative within the limitations of the computational model.

### 3.4. Antimycobacterial Activity in the Context of Structure–Reactivity and Structure–Activity Relationships

Previous studies have demonstrated that the antimycobacterial activity of INH-derived thiosemicarbazides and related heterocycles depends on both the precursor structure and the nature of the substituents. Pitucha et al. reported antimycobacterial activity for pyridine-containing thiosemicarbazides, with 2-pyridyl derivatives showing the highest activity (MIC 7.81–31.25 µg/mL), highlighting the influence of pyridine regioisomerism [20]. Vosátka et al. investigated INH-derived *N*-alkyl semicarbazides and their corresponding 1,3,4-oxadiazoles [18]. Semicarbazides bearing short n-alkyl substituents exhibited activity against *M. tuberculosis* comparable to that of INH (MIC 0.5–2 µM) and inhibited enoyl-ACP reductase (InhA), similarly to INH. Elongation of the alkyl chain reduced their potency. Cyclization to the corresponding 1,3,4-oxadiazoles generally did not improve activity; however, the more lipophilic *N*-dodecyl derivative retained activity against susceptible and drug-resistant *M. tuberculosis* strains (MIC 4–8 µM) through a mechanism distinct from that of INH. The relationship between alkyl-chain length and activity was not linear, indicating that antimycobacterial potency depends on the specific structural features of the compounds rather than on lipophilicity alone.

Although Vosátka et al. also investigated 4-pyridyl-substituted 1,3,4-oxadiazoles [18], their compounds differed structurally from those examined in the present study. In particular, the previously reported derivatives contained aliphatic substituents, whereas the oxadiazoles presented here bear haloaryl groups. These differences, together with variations in the biological assay conditions, limit direct comparisons but highlight the importance of substituent identity in determining the biological properties of the 1,3,4-oxadiazole scaffold.

These literature findings provided a rationale for investigating whether the biological properties of related INH-derived precursors would be retained upon cyclization. However, none of the 1,3,4-oxadiazole, thiazolidin-4-one, or ester-functionalized thiazolidin-4-one derivatives examined in the present study showed appreciable activity against the tested *M. tuberculosis* strains (MIC ≥ 512 µg/mL). Thus, neither the choice of heterocyclic scaffold nor the introduction of additional ester functionality resulted in meaningful antimycobacterial activity under the assay conditions employed.

The uniformly low activity across the investigated series also indicates that the favourable effects associated with specific structural features in previously reported compounds cannot necessarily be extrapolated to structurally distinct derivatives. For example, the enhanced activity associated with the 2-pyridyl moiety in the thiosemicarbazide series reported by Pitucha et al. [20] was not retained following conversion to the heterocyclic scaffolds examined here. Similarly, the presence of a 1,3,4-oxadiazole ring alone was insufficient to confer appreciable activity, consistent with previous findings that the biological properties of this scaffold depend on the nature of its substituents [18]. The introduction of an ester functionality into the thiazolidin-4-one scaffold likewise failed to improve activity. Notably, the 2-pyridyl/*ortho*-halogen arrangement, which favoured thiazolidin-4-one formation, was not associated with enhanced antimycobacterial potency. This finding further illustrates that structural features influencing cyclization outcomes do not necessarily confer favourable biological properties.

Overall, the results indicate that structure–reactivity and structure–activity relationships governing antimycobacterial potency are distinct in the investigated series. Whereas relatively subtle structural changes redirected the cyclization pathway, variation in pyridine regioisomerism, aryl halogen substitution, heterocyclic scaffold, and ester functionality did not yield a meaningful structure–activity relationship. These findings underscore the scaffold- and substituent-dependent nature of antimycobacterial activity and demonstrate that synthetic selectivity alone cannot predict biological potency. Considered alongside previous reports on related INH-derived compounds, the present findings, together with the DFT analysis, help rationalize the observed differences in cyclization behaviour and provide guidance for the design of future INH-derived analogues.

## 4. Materials and Methods

### 4.1. General Experimental Details

All chemicals and solvents used in the synthesis were purchased from commercial sources, namely Merck (Darmstadt, Germany), Fisher Scientific (Waltham, MA, USA), and Polish Reagents (Gliwice, Poland). Unless otherwise stated, all reagents and solvents were used as received without further purification.

Melting points were determined on an Electrothermal Standard 120 VAC melting point apparatus (Cole-Parmer, Wertheim, Germany) and are reported without correction. The ^1^H and ^13^C NMR spectra were recorded using Bruker Avance DPX 600 spectrometers (Billerica, MA, USA). DMSO-*d*_6_ was used as the NMR solvent, with tetramethylsilane (TMS) serving as the internal standard. Chemical shifts are reported in δ values in parts per million (ppm).

Elemental analyses were performed using an AMZ 851 CHX analyzer (PG, Gdańsk, Poland). The measured carbon, hydrogen, and nitrogen contents were within ±0.4% of the corresponding calculated theoretical values.

### 4.2. General Method for the Synthesis of Thiosemicarbazides 1-(Py/Ar)

Pyridinecarbohydrazide (0.0036 mol) was dissolved in anhydrous ethanol (20 mL) in a round-bottom flask. The appropriate isothiocyanate (0.0036 mol) was then added to the resulting solution. The reaction mixture was heated under reflux for approximately 3 h. After cooling to room temperature, the resulting precipitate was collected by filtration, washed with diethyl ether, and dried.

The physicochemical properties of the obtained thiosemicarbazides **1-(Py/Ar)** were consistent with the data reported in the literature and are also available from the Chemical Abstracts Service (CAS). The corresponding CAS Registry Numbers are provided below.

**1-(2-Py/*o*-Cl)**—CAS Registry Number 894234-77-6; **1-(2-Py/*m*-Cl)**—CAS Registry Number 197512-64-4; **1-(2-Py/*p*-Cl)**—CAS Registry Number 195818-67-8; **1-(2-Py/*o*-F)**—CAS Registry Number 891086-61-6; **1-(2-Py/*m*-F)**—CAS Registry Number 891086-37-6; **1-(2-Py/*p*-F)**—CAS Registry Number 195818-65-6; **1-(3-Py/*o*-Cl)**—CAS Registry Number 131090-33-0; **1-(3-Py/*m*-Cl)**—CAS Registry Number 131090-26-1; **1-(3-Py/*p*-Cl)**—CAS Registry Number 127055-57-6; **1-(3-Py/*o*-F)**—CAS Registry Number 479047-48-8; **1-(3-Py/*m*-F)**—CAS Registry Number 891086-53-6; **1-(3-Py/*p*-F)**—CAS Registry Number 195818-66-7; **1-(4-Py/*o*-Cl)**—CAS Registry Number 117080-29-2; **1-(4-Py/*m*-Cl)**—CAS Registry Number 13094-44-5; **1-(4-Py/*p*-Cl)**—CAS Registry Number 74270-72-7; **1-(4-Py/*o*-F)**—CAS Registry Number 199460-69-0; **1-(4-Py/*m*-F)**—CAS Registry Number 891086-45-6; **1-(4-Py/*p*-F)**—CAS Registry Number 636-12-4.

### 4.3. General Method for the Synthesis of Thiazolidin-4-One Derivatives 2-(2-Py/o-Hal) and 3-(Py/Ar)

A mixture of thiosemicarbazide **1-(Py/Ar)** (0.0015 mol), ethyl bromoacetate (0.0015 mol), anhydrous sodium acetate (0.0015 mol), and anhydrous ethanol (5–10 mL) was heated under reflux for 1.5 h. After completion of the reaction, the resulting precipitate was allowed to stand in the reaction flask for 24 h. The precipitate was then collected by filtration, air-dried, and recrystallized from glacial acetic acid or butanol.

*N’*-[3-(2-chlorophenyl)-4-oxo-1,3-thiazolidin-2-ylidene]pyridine-2-carbohydrazide **2-(2-Py/*o*-Cl)**

Yield 70%; m.p. = 182–184 °C. ^1^H NMR (600 MHz, DMSO-*d*_6_) δ (ppm): 4.29 s (2H, CH_2_); 6.94 dd (*J* = 1.5, 7.9 Hz), 7.14 td (*J* = 1.5, 7.8 Hz), 7.32 td (*J* = 1.3, 7.7 Hz), 7.48 dd (*J* = 1.3, 8.0 Hz) (4H, 2-Cl-C_6_H_4_); 7.70–7.74 m, 8.05–8.11 m, 8.75 dt (*J* = 1.3, 4.7 Hz) (4H, 2-Py); 11.52 s (1H, NH). ^13^C NMR (150 MHz, DMSO-*d*_6_) δ (ppm): 30.6; 122.2; 123.4; 125.6; 128.2; 128.6; 130.5; 138.6; 145.2; 149.4; 154.4; 162.9; 169.1. Anal. calc. for C_15_H_11_ClN_4_O_2_S (346.791) (%): C 51.95; H 3.20; N 16.16. Found: C 51.82; H 3.24; N 16.05.

*N’*-[3-(2-fluorophenyl)-4-oxo-1,3-thiazolidin-2-ylidene]pyridine-2-carbohydrazide **2-(2-Py/*o*-F)**

Yield 66%; m.p. = 190–191 °C. ^1^H NMR (600 MHz, DMSO-*d*_6_) δ (ppm): 4.29 d (2H, CH_2_, *J* = 4.1 Hz); 6.92–6.98 m, 7.16 ddd (*J* = 2.0; 5.6; 8.3 Hz), 7.25 ddd (*J* = 2.3; 7.5; 9.8 Hz) (4H, 2-F-C_6_H_4_); 7.72 ddd (*J* = 1.8; 4.8; 6.9 Hz), 8.03–8.12 m, 8.75 d (*J* = 4.6 Hz) (4H, 2-Py); 11.53 s (1H, NH). ^13^C NMR (150 MHz, DMSO-*d*_6_) δ (ppm): 30.6; 116.8 d (*J* = 19.3 Hz); 123.3 d (*J* = 31.8 Hz); 125.5; 126.3 d (*J* = 6.9 Hz); 128.2; 135.6 d (*J* = 12.3 Hz); 138.6; 148.6; 149.4; 152.7; 154.3; 154.8; 162.9; 169.0. Anal. calc. for C_15_H_11_CFN_4_O_2_S (330.337) (%): C 54.54; H 3.36; N 16.96. Found: C 54.39; H 3.41; N 16.82.

*N*-(3-chlorophenyl)-5-(pyridin-2-yl)-1,3,4-oxadiazol-2-amine **3-(2-Py/*m*-Cl)**

Yield 57%; m.p. = 230–232 °C. ^1^H NMR (600 MHz, DMSO-*d*_6_) δ (ppm): 7.07 ddd (*J* = 1.0; 2.0; 7.9 Hz), 7.39 t (*J* = 8.1 Hz), 7.51 ddd (*J* = 1.0; 2.2; 8.3 Hz), 7.56 ddd (*J* = 1.3; 4.8; 7.5 Hz) (4H, 3-Cl-C_6_H_4_); 7.77 t (*J* = 2.0 Hz), 8.00 td (*J* = 1.7; 7.7 Hz), 8.09 dt (*J* = 1.2; 8.0 Hz), 8.60–8.82 m (4H, 2-Py); 11.13 s (1H, NH). ^13^C NMR (150 MHz, DMSO-*d*_6_) δ (ppm): 116.2; 117.0; 122.2; 122.3; 126.0; 131.3; 134.0; 138.1; 140.4; 143.5; 150.5; 158.3; 160.6. Anal. calc. for C_13_H_9_ClN_4_O (272.69) (%): C 57.26; H 3.33; N 20.55. Found: C 57.12; H 3.37; N 20.43.

*N*-(4-chlorophenyl)-5-(pyridin-2-yl)-1,3,4-oxadiazol-2-amine **3-(2-Py/*p*-Cl)**

Yield 62%; m.p. = 261–262 °C. ^1^H NMR (600 MHz, DMSO-*d*_6_) δ (ppm): 7.44 d, 7.64 d (4H, 4-Cl-C_6_H_4_, *J* = 9.0 Hz); 7.51–7.60 m, 8.01 td (*J* = 1.7; 7.7 Hz), 8.09 d (*J* = 7.9 Hz), 8.73 d (*J* = 4.8 Hz) (4H, 2-Py); 11.04 s (1H, NH). ^13^C NMR (150 MHz, DMSO-*d*_6_) δ (ppm): 119.2; 122.3; 126.0; 126.2; 129.5; 138.0; 138.2; 143.6; 150.5; 158.2; 160.8. Anal. calc. for C_13_H_9_ClN_4_O (272.69) (%): C 57.26; H 3.33; N 20.55. Found: C 57.21; H 3.31; N 20.48.

*N*-(3-fluorophenyl)-5-(pyridin-2-yl)-1,3,4-oxadiazol-2-amine **3-(2-Py/*m*-F)**

Yield 47%; m.p. = 217–218 °C. ^1^H NMR (600 MHz, DMSO-*d*_6_) δ (ppm): 6.86 td (*J* = 2.5; 8.5 Hz), 7.36 dd (*J* = 2.0; 8.1 Hz), 7.39–7.43 m, 7.54–7.59 m, 8.02 td (*J* = 1.6; 7.7 Hz), 8.10 d (*J* = 7.9 Hz), 8.73 d (*J* = 4.8 Hz) (8H, 3-F-C_6_H_4_ and 2-Py), 11.13 s (1H, NH). ^13^C NMR (150 MHz, DMSO-*d*_6_) δ (ppm): 104.5 d (*J* = 26.4 Hz); 108.9 d (*J* = 20.7 Hz); 113.7; 122.3; 126.1; 131.3 d (*J* = 9.8 Hz); 138.2; 140.8 d (*J* = 10.9 Hz); 143.5; 149.1; 150.6; 158.2; 160.7. Anal. calc. for C_13_H_9_FN_4_O (256.235) (%): C 60.94; H 3.54; N 21.87. Found: C 60.79; H 3.58; N 21.74.

*N*-(4-fluorophenyl)-5-(pyridin-2-yl)-1,3,4-oxadiazol-2-amine **3-(2-Py/*p*-F)**

Yield 64%; m.p. = 230–232 °C. ^1^H NMR (600 MHz, DMSO-*d*_6_) δ (ppm): 7.23 t (*J* = 8.9 Hz), 7.57 dd (*J* = 5.4; 6.9 Hz), 7.63 dd (*J* = 4.7; 8.9 Hz), 8.00–8.02 m, 8.08 d (*J* = 7.9 Hz), 8.72 d (*J* = 4.8 Hz) (8H, 4-F-C_6_H_4_ and 2-Py), 10.85 s (1H, NH). ^13^C NMR (150 MHz, DMSO-*d*_6_) δ (ppm): 116.2 d (*J* = 22.4 Hz); 119.3 d (*J* = 8.1 Hz); 122.2; 125.9; 135.5; 138.1; 143.6; 149.1; 150.5; 158.1; 161.0. Anal. calc. for C_13_H_9_FN_4_O (256.235) (%): C 60.94; H 3.54; N 21.87. Found: C 60.87; H 3.49; N 21.79.

*N*-(2-chlorophenyl)-5-(pyridin-3-yl)-1,3,4-oxadiazol-2-amine **3-(3-Py/*o*-Cl)** (CAS Registry Number 905783-93-9)

Yield 48%; m.p. = 184–186 °C. ^1^H NMR (600 MHz, DMSO-*d*_6_) δ (ppm): 7.17 td (*J* = 1.2; 7.8 Hz); 7.38–7.44 m, 7.53 dd (*J* = 1.2; 8.1 Hz); 7.62 dd (*J* = 4.8; 8.1 Hz) (4H, 2-Cl-C_6_H_4_); 8.05 d (*J* = 8.1 Hz); 8.25 dt (*J* = 1.8; 8.1 Hz); 8.75 dd (*J* = 1.5; 4.8 Hz); 9.06 d (*J* = 1.5 Hz) (4H, 2-Py); 10.18 s (1H, NH). ^13^C NMR (150 MHz, DMSO-*d*_6_) δ (ppm): 120.8; 122.8; 124.6; 124.8; 125.5; 128.5; 130.3; 133.6; 135.6; 146.8; 152.1; 157.1; 161.3. Anal. calc. for C_13_H_9_ClN_4_O (272.69) (%): C 57.26; H 3.33; N 20.55. Found: C 57.23; H 3.28; N 20.54.

*N*-(3-chlorophenyl)-5-(pyridin-3-yl)-1,3,4-oxadiazol-2-amine **3-(3-Py/*m*-Cl)** (CAS Registry Number 2944473-73-6)

Yield 73%; m.p. = 232–234 °C. ^1^H NMR (600 MHz, DMSO-*d*_6_) δ (ppm): 7.07 dd (*J* = 1.2, 8.1 Hz); 7.38 t (*J* = 8.1 Hz); 7.51 dd (*J* = 1.2, 8.1 Hz); 7.61 dd (*J* = 4.8, 8.1 Hz) (4H, 3-Cl-C_6_H_4_); 7.77 t (*J* = 1.8 Hz); 8.24 dt (*J* = 1.8, 8.1 Hz); 8.75 dd (*J* = 1.8, 4.8 Hz); 9.05 d (*J* = 1.8 Hz) (4H, 3-Py); 11.05 s (1H, NH). ^13^C NMR (150 MHz, DMSO-*d*_6_) δ (ppm): 116.2; 117.0; 120.7; 122.2; 124.8; 131.3; 133.6; 134.0; 140.4; 146.8; 152.1; 156.6; 160.3. Anal. calc. for C_13_H_9_ClN_4_O (272.69) (%): C 57.26; H 3.33; N 20.55. Found: C 57.20; H 3.21; N 20.51.

*N*-(4-chlorophenyl)-5-(pyridin-3-yl)-1,3,4-oxadiazol-2-amine **3-(3-Py/*p*-Cl)** (CAS Registry Number 2944473-75-8)

Yield 54%; m.p. = 254–256 °C. ^1^H NMR (600 MHz, DMSO-*d*_6_) δ (ppm): 7.44 d (*J* = 9.0 Hz); 7.60–7.66 m, 8.26 t (*J* = 1.8; 8.1 Hz); 8.75 dd (*J* = 1.8; 8.1 Hz); 9.07 d (*J* = 1.5 Hz) (8H, 4-Cl-C_6_H_4_ and 3-Py); 10.98 s (1H, NH). ^13^C NMR (150 MHz, DMSO-*d*_6_) δ (ppm): 119.2; 120.8; 124.8; 126.2; 129.5; 133.6; 138.0; 146.8; 152.1; 152.9; 156.5; 160.5. Anal. calc. for C_13_H_9_ClN_4_O (272.69) (%): C 57.26; H 3.33; N 20.55. Found: C; 57.18; H 3.29; N 20.48.

*N*-(2-fluorophenyl)-5-(pyridin-3-yl)-1,3,4-oxadiazol-2-amine **3-(3-Py/*o*-F)**

Yield 48%; m.p. = 206–208 °C. ^1^H NMR (600 MHz, DMSO-*d*_6_) δ (ppm): 7.08–7.15 m, 7.22–7.34 m, 8.10 td (4H, 2-F-C_6_H_4_, *J* = 1.5; 8.1 Hz); 7.62 ddd (*J* = 0.9; 5.1 Hz), 8.24 dt (*J* = 1.8; 8.1 Hz), 8.75 dd (*J* = 1.8; 7.8 Hz), 9.06 dd (*J* = 0.9; 2.1 Hz) (4H, 3-Py); 10.61 s (1H, NH). ^13^C NMR (150 MHz, DMSO-*d*_6_) δ (ppm): 116.1 d (*J* = 18.5 Hz); 120.8; 121.3; 124.4 d (*J* = 6.9 Hz); 124.8; 125.3 d (*J* = 3.0 Hz); 126.8 d (*J* = 11.2 Hz); 133.6; 146.8; 152.1; 153.7; 156.9; 161.0. Anal. calc. for C_13_H_9_FN_4_O (256.235) (%): C 60.94; H 3.54; N 21.87. Found: C 60.82; H 3.49; N 21.76.

*N*-(3-fluorophenyl)-5-(pyridin-3-yl)-1,3,4-oxadiazol-2-amine **3-(3-Py/*m*-F)**

Yield 70%; m.p. = 244–245 °C. ^1^H NMR (600 MHz, DMSO-*d*_6_) δ (ppm): 6.84–6.87 m, 7.37–7.43 m, 7.56 dt (*J* = 2.2; 11.5 Hz) (4H, 3-F-C_6_H_4_); 7.63 dd (*J* = 4.8; 8.0 Hz), 8.26 dt (*J* = 1.9; 8.0 Hz), 8.76 dd (*J* = 1.6; 4.8 Hz), 9.07 d (*J* = 2.2 Hz) (4H, 3-Py); 11.08 s (1H, NH). ^13^C NMR (150 MHz, DMSO-*d*_6_) δ (ppm): 104.5 d (*J* = 26.4 Hz); 108.9 d (*J* = 20.7 Hz); 113.7; 120.8; 124.8; 131.3 (*J* = 9.8 Hz); 133.7; 140.8 d (*J* = 10.9 Hz); 146.8; 152.1; 156.6; 160.4; 162.2; 163.8. Anal. calc. for C_13_H_9_FN_4_O (256.235) (%): C 60.94; H 3.54; N 21.87. Found: C 60.76; H 3.52; N 21.83.

*N*-(4-fluorophenyl)-5-(pyridin-3-yl)-1,3,4-oxadiazol-2-amine **3-(3-Py/*p*-F)** (CAS Registry Number 1392007-16-7)

Yield 73%; m.p. = 244–246 °C. ^1^H NMR (600 MHz, DMSO-*d*_6_) δ (ppm): 7.23 t (*J* = 8.8 Hz), 7.63 ddd (*J* = 5.0; 8.6; 13.3 Hz), 8.25 dt (*J* = 1.8; 8.0 Hz), 8.75 dd (*J* = 1.5; 4.8 Hz), 9.06 d (*J* = 1.6 Hz) (8H, 4-F-C_6_H_4_ and 3-Py); 10.84 s (1H, NH). ^13^C NMR (150 MHz, DMSO-*d*_6_) δ (ppm): 124.1; 127.3; 128.5; 133.4; 135.8; 148.9; 153.0; 162.8; 163.6; 164.4; 167.5. Anal. calc. for C_13_H_9_FN_4_O (256.235) (%): C 60.94; H 3.54; N 21.87. Found: C 60.89; H 3.47; N 21.91.

*N*-(2-chlorophenyl)-5-(pyridin-4-yl)-1,3,4-oxadiazol-2-amine **3-(4-Py/*o*-Cl)** (CAS Registry Number 117080-46-3)

Yield 51%; m.p. = 160–162 °C. ^1^H NMR (600 MHz, DMSO-*d*_6_) δ (ppm): 7.19 td (*J* = 1.5; 7.7 Hz), 7.33–7.48 m, 7.55 dd (*J* = 1.4; 8.1 Hz), 8.01 d (*J* = 8.5 Hz) (4H, 2-Cl-C_6_H_4_); 7.84 d, 8.81 d (4H, 4-Py, *J* = 6.0 Hz); 10.31 s (1H, NH). ^13^C NMR (150 MHz, DMSO-*d*_6_) δ (ppm): 119.9; 123.4; 125.1; 125.9; 128.5; 130.4; 131.6; 135.4; 150.9; 157.3; 161.8. Anal. calc. for C_13_H_9_ClN_4_O (272.69) (%): C 57.26; H 3.33; N 20.55. Found: C 57.31; H 3.37; N 20.61.

*N*-(3-chlorophenyl)-5-(pyridin-4-yl)-1,3,4-oxadiazol-2-amine **3-(4-Py/*m*-Cl)**

Yield 49%; m.p. = 268–270 °C. ^1^H NMR (600 MHz, DMSO-*d*_6_) δ (ppm): 7.07–7.11 m, 7.40 t (*J* = 8.1 Hz), 7.51–7.55 m, 7.78 t (*J* = 1.8 Hz) (4H, 3-Cl-C_6_H_4_); 7.81 d, 8.79 d (4H, 4-Py, *J* = 6.0 Hz); 11.13 s (1H, NH). ^13^C NMR (150 MHz, DMSO-*d*_6_) δ (ppm): 116.3; 117.2; 119.8; 122.4; 131.2; 131.3; 134.0; 140.3; 151.3; 157.0; 160.6. Anal. calc. for C_13_H_9_ClN_4_O (272.69) (%): C 57.26; H 3.33; N 20.55. Found: C 57.22; H 3.25; N 20.58.

*N*-(4-chlorophenyl)-5-(pyridin-4-yl)-1,3,4-oxadiazol-2-amine **3-(4-Py/*p*-Cl)** (CAS Registry Number 117080-47-4)

Yield 47%; m.p. = 282–284 °C. ^1^H NMR (600 MHz, DMSO-*d*_6_) δ (ppm): 7.44 d, 7.65 d (4H, 4-Cl-C_6_H_4_, *J* = 9.0 Hz); 7.81 d, 8.79 d (4H, 4-Py, *J* = 6.0 Hz); 11.05 s (1H, NH). ^13^C NMR (150 MHz, DMSO-*d*_6_) δ (ppm): 119.3; 119.7; 126.3; 129.5; 131.2; 137.8; 151.3; 156.9; 160.8. Anal. calc. for C_13_H_9_ClN_4_O (272.69) (%): C 57.26; H 3.33; N 20.55. Found: C 57.39; H 3.35; N 20.52.

*N*-(2-fluorophenyl)-5-(pyridin-4-yl)-1,3,4-oxadiazol-2-amine **3-(4-Py/*o*-F)** (CAS Registry Number 1787578-42-0)

Yield 79%; m.p. = 210–212 °C. ^1^H NMR (600 MHz, DMSO-*d*_6_) δ (ppm): 7.09–7.16 m, 7.23–7.34 m, 8.08 td (4H, 2-F-C_6_H_4_, *J* = 1.8; 8.4 Hz); 7.80 d, 8.79 d (4H, 4-Py, *J* = 6.0 Hz); 10.69 s (1H, NH). ^13^C NMR (150 MHz, DMSO-*d*_6_) δ (ppm): 116.1 d (*J* = 18.5 Hz); 119.7; 121.6; 124.6 d (*J* = 7.0 Hz); 125.3 (*J* = 3.0 Hz); 126.6 d (*J* = 11.2 Hz); 131.2; 151.3; 152.2; 153.8; 157.2; 161.4. Anal. calc. for C_13_H_9_FN_4_O (256.235) (%): C 60.94; H 3.54; N 21.87. Found: C 60.96; H 3.61; N 21.99.

*N*-(3-fluorophenyl)-5-(pyridin-4-yl)-1,3,4-oxadiazol-2-amine **3-(4-Py/*m*-F)**

Yield 71%; m.p. = 233–235 °C. ^1^H NMR (600 MHz, DMSO-*d*_6_) δ (ppm): 6.87 td (*J* = 2.1; 8.3 Hz), 7.37–7.43 m, 7.55 dt (*J* = 2.3; 11.5 Hz) (4H, 3-F-C_6_H_4_); 7.81 d, 8.80 d (4H, 4-Py, *J* = 6.0 Hz); 11.16 s (1H, NH). ^13^C NMR (150 MHz, DMSO-*d*_6_) δ (ppm): 104.6 d (*J* = 26.7 Hz); 109.1 d (*J* = 21.0 Hz); 113.8; 119.8; 131.2; 131.3 (*J* = 9.5 Hz); 140.6 d (*J* = 11.1 Hz); 151.3; 156.9; 160.7; 162.2; 163.8. Anal. calc. for C_13_H_9_FN_4_O (256.235) (%): C 60.94; H 3.54; N 21.87. Found: C 60.91; H 3.59; N 21.96.

*N*-(4-fluorophenyl)-5-(pyridin-4-yl)-1,3,4-oxadiazol-2-amine **3-(4-Py/*p*-F)**

Yield 68%; m.p. = 257–259 °C. ^1^H NMR (600 MHz, DMSO-*d*_6_) δ (ppm): 7.23 t (*J* = 8.9 Hz), 7.56–7.70 m, 8.25 dt (*J* = 1.8; 8.0 Hz), 8.75 dd (*J* = 1.6; 4.8 Hz), 9.06 d (*J* = 1.5 Hz) (8H, 4-F-C_6_H_4_ and 4-Py); 10.83 s (1H, NH). ^13^C NMR (150 MHz, DMSO-*d*_6_) δ (ppm): 116.2 d (*J* = 22.4 Hz); 119.3 d (*J* = 7.7 Hz); 120.9; 124.8; 133.6; 135.4; 152.0; 156.4; 157.1; 158.7; 160.7. Anal. calc. for C_13_H_9_FN_4_O (256.235) (%): C 60.94; H 3.54; N 21.87. Found: C 60.85; H 3.51; N 21.72.

### 4.4. General Synthetic Procedure for the (4-Oxothiazolidin-5-Ylidene)Acetic Acid Derivatives 4-(Py/Ar)

Thiosemicarbazide **1-(Py/Ar)** (0.0016 mol), dimethyl acetylenedicarboxylate (0.0016 mol), and methanol (15 mL) were heated under reflux for 2 h. After completion of the heating, the reaction mixture was allowed to stand for 24 h. The resulting precipitate was collected by filtration and left to dry. The obtained compounds were recrystallized from butanol or isopropanol.

Methyl {3-(2-chlorophenyl)-4-oxo-2-[2-(pyridine-2-carbonyl)hydrazinylidene]-1,3-thiazolidin-5-ylidene}acetate **4-(2-Py/*o*-Cl)**

Yield 85%; m.p. = 184–186 °C. ^1^H NMR (600 MHz, DMSO-*d*_6_) δ (ppm): 3.77 s (3H, OCH_3_); 7.03 s (1H, CH=); 7.04 dd (*J* = 1.5; 7.8 Hz), 7.24 td (*J* = 1.5; 7.8 Hz), 7.40 td (*J* = 1.2; 7.7 Hz), 7.56 dd (*J* = 1.1; 8.0 Hz) (4H, 2-Cl-C_6_H_4_); 7.73–7.77 m, 8.07–8.12 m, 8.78 d (4H, 2-Py, *J* = 4.7 Hz); 11.82 s (1H, NHCO). ^13^C NMR (150 MHz, DMSO-*d*_6_) δ (ppm): 53.3; 118.7; 122.3; 123.5; 125.4; 127.2; 128.4; 128.8; 130.7; 137.3; 138.7; 144.3; 148.2; 149.5; 150.4; 161.7; 163.0; 166.0. Anal. calc. for C_18_H_13_ClN_4_O_4_S (416.838) (%): C 51.87; H 3.14; N 13.44. Found: C 51.79; H 3.18; N 13.38.

Methyl {3-(3-chlorophenyl)-4-oxo-2-[2-(pyridine-2-carbonyl)hydrazinylidene]-1,3-thiazolidin-5-ylidene}acetate **4-(2-Py/*m*-Cl)**

Yield 81%; m.p. = 238–240 °C. ^1^H NMR (600 MHz, DMSO-*d*_6_) δ (ppm): 3.81 s (3H, OCH_3_); 6.89 s (1H, CH=); 7.49–7.52 m, 7.60–7.67 m, 8.03–8.05 m, 8.71–8.73 m (8H, 3-Cl-C_6_H_4_ and 2-Py); 11.35 s (1H, NHCO). ^13^C NMR (150 MHz, DMSO-*d*_6_) δ (ppm): 53.2; 115.8; 122.8; 127.4; 128.8; 129.7; 131.3; 136.1; 138.5; 142.9; 143.7; 144.6; 146.9; 149.2; 160.1; 162.3; 163.7; 165.3. Anal. calc. for C_18_H_13_ClN_4_O_4_S (416.838) (%): C 51.87; H 3.14; N 13.44. Found: C 51.73; H 3.13; N 13.55.

Methyl {3-(4-chlorophenyl)-4-oxo-2-[2-(pyridine-2-carbonyl)hydrazinylidene]-1,3-thiazolidin-5-ylidene}acetate **4-(2-Py/*p*-Cl)**

Yield 73%; m.p. = 246–248 °C. ^1^H NMR (600 MHz, DMSO-*d*_6_) δ (ppm): 3.81 s (3H, OCH_3_); 6.89 s (1H, CH=); 7.55 d, 7.65–7.69 m, 8.02–8.05 m, 8.71–8.73 m (8H, 4-Cl-C_6_H_4_ and 2-Py, *J* = 9,0 Hz); 11.34 s (1H, NHCO). ^13^C NMR (150 MHz, DMSO-*d*_6_) δ (ppm): 53.2; 115.9; 122.8; 127.5; 129.7; 130.6; 133.3; 134.2; 138.5; 140.9; 145.3; 149.2; 157.6; 161.5; 163.8; 166.5. Anal. calc. for C_18_H_13_ClN_4_O_4_S (416.838) (%): C 51.87; H 3.14; N 13.44. Found: C 51.94; H 3.11; N 13.51.

Methyl {3-(3-chlorophenyl)-4-oxo-2-[2-(pyridine-3-carbonyl)hydrazinylidene]-1,3-thiazolidin-5-ylidene}acetate **4-(3-Py/*m*-Cl)**

Yield 80%; m.p. = 208–210 °C. ^1^H NMR (600 MHz, DMSO-*d*_6_) δ (ppm): 3.81 s (3H, OCH_3_); 6.87 s (1H, CH=); 7.51–7.68 m, 8.17 d (*J* = 7.8 Hz), 8.75 d (*J* = 3.6 Hz), 8.97 s (8H, 3-Cl-C_6_H_4_ and 3-Py); 11.53 s (1H, NHCO). ^13^C NMR (150 MHz, DMSO-*d*_6_) δ (ppm): 53.2; 115.8; 124.1; 127.8; 128.9; 129.2; 129.7; 131.3; 133.6; 135.79; 135.84; 141.2; 148.9; 152.8; 157.1; 162.7; 163.8; 166.4. Anal. calc. for C_18_H_13_ClN_4_O_4_S (416.838) (%): C 51.87; H 3.14; N 13.44. Found: C 51.83; H 3.09; N 13.35.

Methyl {3-(4-chlorophenyl)-4-oxo-2-[2-(pyridine-3-carbonyl)hydrazinylidene]-1,3-thiazolidin-5-ylidene}acetate **4-(3-Py/*p*-Cl)**

Yield 83%; m.p. = 212–214 °C. ^1^H NMR (600 MHz, DMSO-*d*_6_) δ (ppm): 3.81 s (3H, OCH_3_); 6.87 s (1H, CH=); 7.44 d (*J* = 9.0 Hz), 7.60–7.67 m, 8.17 d (*J* = 7.8 Hz), 8.74–8.76 m, 8.97 s (8H, 4-Cl-C_6_H_4_ and 3-Py); 11.51 s (1H, NHCO). ^13^C NMR (150 MHz, DMSO-*d*_6_) δ (ppm): 53.2; 119.2; 124.1; 124.8; 129.5; 129.7; 130.7; 133.7; 135.8; 141.4; 146.8; 148.9; 152.7; 160.5; 163.8; 166.4. Anal. calc. for C_18_H_13_ClN_4_O_4_S (416.838) (%): C 51.87; H 3.14; N 13.44. Found: C 51.88; H 3.21; N 13.47.

Methyl {3-(4-fluorophenyl)-4-oxo-2-[2-(pyridine-3-carbonyl)hydrazinylidene]-1,3-thiazolidin-5-ylidene}acetate **4-(3-Py/*p*-F)**

Yield 72%; m.p. = 222–224 °C. ^1^H NMR (600 MHz, DMSO-*d*_6_) δ (ppm): 3.81 s (3H, OCH_3_); 6.87 s (1H, CH=); 7.41 t (*J* = 8.7 Hz), 7.53–7.59 m, 8.17 d (*J* = 7.8 Hz), 8.75 dd (*J* = 1.5; 7.8 Hz), 8.97 s (8H, 4-F-C_6_H_4_ and 3-Py); 11.51 s (1H, NHCO). ^13^C NMR (150 MHz, DMSO-*d*_6_) δ (ppm): 53.2; 115.7; 116.6 d (*J* = 22.9 Hz); 124.1; 130.7; 131.0 d (*J* = 9.1 Hz); 135.8; 136.8; 139.1; 141.3; 148.9; 152.7; 161.6; 162.0; 164.0; 166.4. Anal. calc. for C_18_H_13_FN_4_O_4_S (400.384) (%): C 54.00; H 3.27; N 13.99. Found: C 53.92; H 3.31; N 13.91.

Methyl {3-(3-chlorophenyl)-4-oxo-2-[2-(pyridine-4-carbonyl)hydrazinylidene]-1,3-thiazolidin-5-ylidene}acetate **4-(4-Py/*m*-Cl)**

Yield 74%; m.p. = 178–180 °C. ^1^H NMR (600 MHz, DMSO-*d*_6_) δ (ppm): 3.81 s (3H, OCH_3_); 6.88 s (1H, CH=); 7.21–7.24 m, 7.36 t, 7.44–7.46 m (4H, 3-Cl-C_6_H_4_, *J* = 8.1 Hz); 7.73 d, 8.78 d (4H, 4-Py, *J* = 5.7 Hz); 11.61 s (1H, NHCO). ^13^C NMR (150 MHz, DMSO-*d*_6_) δ (ppm): 53.2; 115.9; 116.4; 119.8; 121.9; 127.8; 128.9; 129.7; 133.6; 134.0; 135.8; 150.8; 151.4; 160.7; 163.8; 166.4. Anal. calc. for C_18_H_13_ClN_4_O_4_S (416.838) (%): C 51.87; H 3.14; N 13.44. Found: C 51.73; H 3.13; N 13.55.

Methyl {3-(4-fluorophenyl)-4-oxo-2-[2-(pyridine-4-carbonyl)hydrazinylidene]-1,3-thiazolidin-5-ylidene}acetate **4-(4-Py/*p*-F)**

Yield 76%; m.p. = 242–244 °C. ^1^H NMR (600 MHz, DMSO-*d*_6_) δ (ppm): 3.81 s (3H, OCH_3_); 6.87 s (1H, CH=); 7.41 t (*J* = 8.8 Hz), 7.56 dd (*J* = 5.0; 8.8 Hz) (4H, 4-F-C_6_H_4_); 7.73 d, 8.76 d (4H, 4-Py, *J* = 5.9 Hz); 11.59 s (1H, NHCO). ^13^C NMR (150 MHz, DMSO-*d*_6_) δ (ppm): 53.2; 115.8; 116.6 d (*J* = 23.3 Hz); 121.9; 130.7 d (*J* = 3.0 Hz); 131.0 d (*J* = 8.9 Hz); 131.1; 140.5; 141.3; 150.8; 158.1; 160.8; 162.5; 164.0; 166.5. Anal. calc. for C_18_H_13_FN_4_O_4_S (400.384) (%): C 54.00; H 3.27; N 13.99. Found: C 53.95; H 3.35; N 14.02.

### 4.5. Antimycobacterial Activity

The antimycobacterial potential of the newly synthesized derivatives was investigated at the Department of Microbiology, National Tuberculosis and Lung Diseases Research Institute (Warsaw, Poland). Biological activity was determined against the reference strain *Mycobacterium tuberculosis* H_37_Rv (ATCC 25618) and three clinical isolates obtained from tuberculosis patients. Among the tested isolates, strain 36/26 displayed resistance to both isoniazid and rifampicin, whereas strain 3715 was resistant only to isoniazid. In contrast, strain 192 remained susceptible to all first-line antitubercular drugs routinely used in clinical practice.

The clinical isolates originated from the strain collection of the National Reference Laboratory of Mycobacteria and had previously been characterized using standard microbiological identification procedures and drug susceptibility testing. The panel of strains was selected to represent different resistance phenotypes, including both drug-susceptible and multidrug-resistant isolates. Collection and use of the clinical material for research purposes were approved by the appropriate Bioethics Committee (approval No. KB-55/2014).

Antimycobacterial activity was evaluated by the broth microdilution method following the recommendations of CLSI document M24 (3rd edition) [46]. Experiments were performed in sterile 96-well microplates containing Middlebrook 7H9 medium supplemented with OADC enrichment and Tween 80. The assay protocol was based on a previously reported procedure [47]. Bacterial suspensions were prepared from freshly grown cultures on Löwenstein–Jensen medium and adjusted to the turbidity of a 0.5 McFarland standard. Prior to inoculation, the suspensions were diluted 100-fold.

Test compounds were initially dissolved in dimethyl sulfoxide (DMSO) and subsequently subjected to serial two-fold dilutions to yield final concentrations between 0.0625 and 512 μg/mL. Aliquots of the bacterial inoculum (100 μL) were added to each well. Positive growth controls and sterility controls were included in every experiment. Following incubation at 37 °C for 21 days, Alamar Blue reagent was introduced into each well and the plates were incubated for a further 24 h. The minimum inhibitory concentration (MIC) was recorded as the lowest concentration of the tested compound that prevented visible bacterial growth, as evidenced by the absence of a color change in the indicator system [48,49]. Isoniazid and rifampicin were included as reference drugs.

### 4.6. Computational Methodology

Initially ester-functionalized **1-(2-Py/*o*-Cl)** and **1-(3-Py/*o*-Cl)** were subjected to conformational analysis as implemented in the HyperChem program [50], using the OPLS force field [51,52]. The structures of the most stable conformers of both compounds were subsequently optimized at the DFT level using the ωB97x-D functional [53,54] expressed in the def2-SVP basis set [55] combined with a continuum solvent model [56] of ethanol as implemented in the Gaussian program [57]. All subsequent calculations were performed at this theory level. Vibrational analysis was used to identify stationary points (3n-6 real normal modes for stable structures and 3n-7 for transition states). Relaxed scans were carried out with a 0.1 Å step for distances and 1 deg for angles. The graphic was obtained with the GaussView 6.0 program [58], which was also used in the preparation of Gaussian input files and analysis of output files.

## 5. Conclusions

The present study demonstrates a pronounced substrate-dependent divergence in the cyclization of isoniazid-derived thiosemicarbazides. Under identical reaction conditions, relatively small changes in the pyridine regioisomerism and aryl substitution pattern were sufficient to determine whether the reaction afforded a thiazolidin-4-one or a 1,3,4-oxadiazole derivative. In particular, the combination of a 2-pyridyl moiety with an *ortho*-fluoro or *ortho*-chloro substituent consistently favoured formation of the targeted thiazolidin-4-one scaffold, whereas most other substitution patterns resulted predominantly in oxadiazole formation. The experimental findings therefore establish a clear and reproducible structure–reactivity relationship within this series.

DFT calculations show that acetate-assisted proton transfer is required to render the cyclization pathways energetically accessible and that the intrinsic ring-closing barriers differ only slightly between **1-(2-Py/*o*-Cl)** and **1-(3-Py/*o*-Cl)**. The principal substrate-dependent feature is the markedly lower trans-to-cis barrier of the N9-deprotonated **1-(3-Py/*o*-Cl)** intermediate, which is qualitatively consistent with its greater tendency to form 1,3,4-oxadiazole. The present continuum solvent model therefore provides a plausible mechanistic explanation for the experimental trend, but not a quantitative prediction of product ratios.

The biological evaluation, in contrast, revealed no corresponding structure–activity relationship. Neither the predominant oxadiazole series nor the thiazolidin-4-one derivatives obtained in the initial cyclization study showed appreciable activity against the investigated *M. tuberculosis* strains. Likewise, incorporation of an additional ester substituent into a second, independently prepared series of thiazolidin-4-ones did not improve antimycobacterial potency. These findings are particularly relevant in light of previous reports of antimycobacterial activity among related pyridyl thiosemicarbazides, indicating that the biological potential associated with the precursor framework was not retained following conversion to the heterocyclic scaffolds investigated here.

The study therefore highlights an important distinction between structure–reactivity and structure–activity relationships, which may be strongly scaffold-dependent. Structural features that substantially alter the energetic accessibility of competing chemical pathways do not necessarily confer a corresponding biological advantage, while structural determinants of biological activity in one chemotype may lose their significance following a transformation that changes the molecular scaffold.

Although the investigated compounds did not emerge as potent antimycobacterial candidates, the negative biological results are nevertheless informative from a medicinal chemistry perspective. They define the limitations of the present chemical space and indicate that further optimization of these particular thiazolidin-4-one and oxadiazole frameworks, within the substitution patterns explored here, is unlikely to provide a straightforward route to improved antimycobacterial activity. At the same time, the pronounced and reproducible substrate control observed in the cyclization reactions provides a useful basis for directing future synthetic efforts toward alternative structural motifs and for exploiting substrate-controlled reactivity in the preparation of new isoniazid-derived chemical entities.

Overall, the principal contribution of this work is the identification and mechanistic interpretation of a clear substrate-controlled divergence between two competing heterocyclization pathways, together with the demonstration that this strong chemical structure–reactivity relationship is not paralleled by a corresponding structure–activity relationship against *M. tuberculosis*. The results therefore provide both a mechanistic understanding of the underlying synthetic chemistry and a defined biological framework for guiding the future development of related isoniazid-derived compounds.

## Figures and Tables

**Table 2:** Gibbs free energetics associated with conformational rotation in the N9-deprotonated reactants.

Reactant	G_reactant_	G_TS_	G_product_	ΔG_activation_	ΔG_reaction_
**1-(2-Py/*o*-Cl)**	−1958.940927	−1958.92692	−1958.945452	8.8	−2.8
**1-(3-Py/*o*-Cl)**	−1958.944719	−1958.93974	−1958.949620	3.1	−3.1

## Data Availability

The data are contained within the article and Supplementary Materials.

## Supplementary Materials

The following supporting information can be downloaded at: https://www.mdpi.com/article/10.3390/ijms27219477/s1.
