# Supplementary material for: Pyridine Regioisomerism and Aryl Substitution Govern the Divergent Cyclization of Isoniazid-Derived Thiosemicarbazides: Mechanistic DFT Analysis and Antimycobacterial Evaluation
Source: Int J Mol Sci. 2026 Oct 23;27(21):9477. doi: 10.3390/ijms27219477 (PMC13763554; doi:10.3390/ijms27219477)
Supplement: Supplementary file 1 [file ijms-27-09477-s001.zip › ijms-4623521-supplementary.pdf]

Supplementary materials for:

Pyridine regioisomerism and aryl substitution govern the divergent cyclization of isoniazid-derived thiosemicarbazides: mechanistic DFT analysis and antimycobacterial evaluation

Agata Paneth [1], Nazar Trotsko [1], Agnieszka Głogowska [2], Ewa Augustynowicz-Kopeć [2], and Piotr Paneth [3,*]

[1] Department of Organic Chemistry, Medical University of Lublin, Chodzki 4A, 20-093 Lublin, Poland; agata.paneth@umlub.edu.pl (A.P.); nazar.trotsko@umlub.edu.pl (N.T.)
[2] Department of Microbiology, National Tuberculosis and Lung Diseases Research Institute, Plocka 26, 01-138 Warsaw, Poland; a.glogowska@igichp.edu.pl (A.G.); e.kopec@igichp.edu.pl (E.A.-K.)
[3] Institute of Applied Radiation Chemistry, Faculty of Chemistry, Lodz University of Technology, Zeromskiego 116, 90-924 Lodz, Poland; piotr.paneth@p.lodz.pl (P.P.)
* Correspondence: piotr.paneth@p.lodz.pl

Table of contents

1. The ^{1}H and ^{13}C NMR spectra of compound **2-(2-Py/*o*-Cl)**……………………………………….…..S2
2. The ^{1}H and ^{13}C NMR spectra of compound **2-(2-Py/*o*-F)**……………………………..………...S3
3. The ^{1}H and ^{13}C NMR spectra of compounds **3-(Py/Ar)**……………………………………………S4
4. The ^{1}H and ^{13}C NMR spectra of compounds **4-(Py/Ar)**…………………………..…………....S20
5. Optimization of reaction condition and isolated yields of the target compounds…………….S28

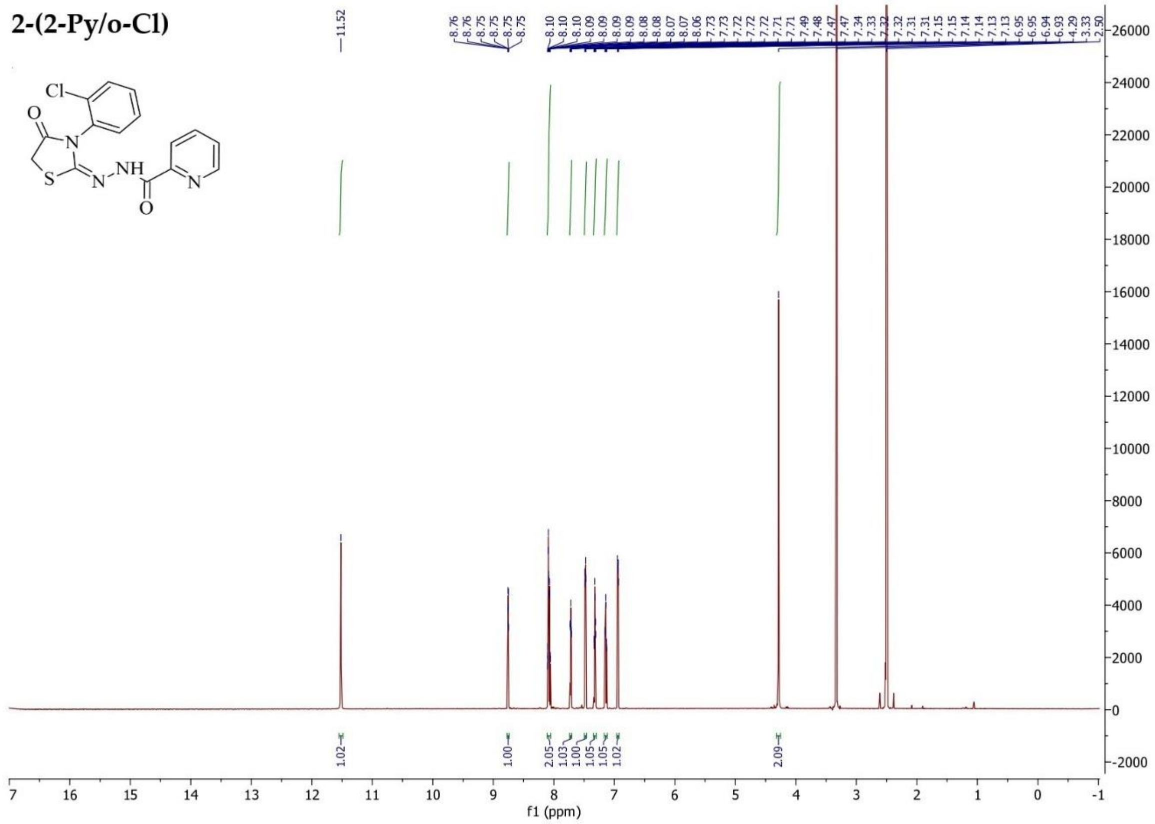

Figure S1. The ^{1}H NMR spectrum of compound **2-(2-Py/*o*-Cl)**

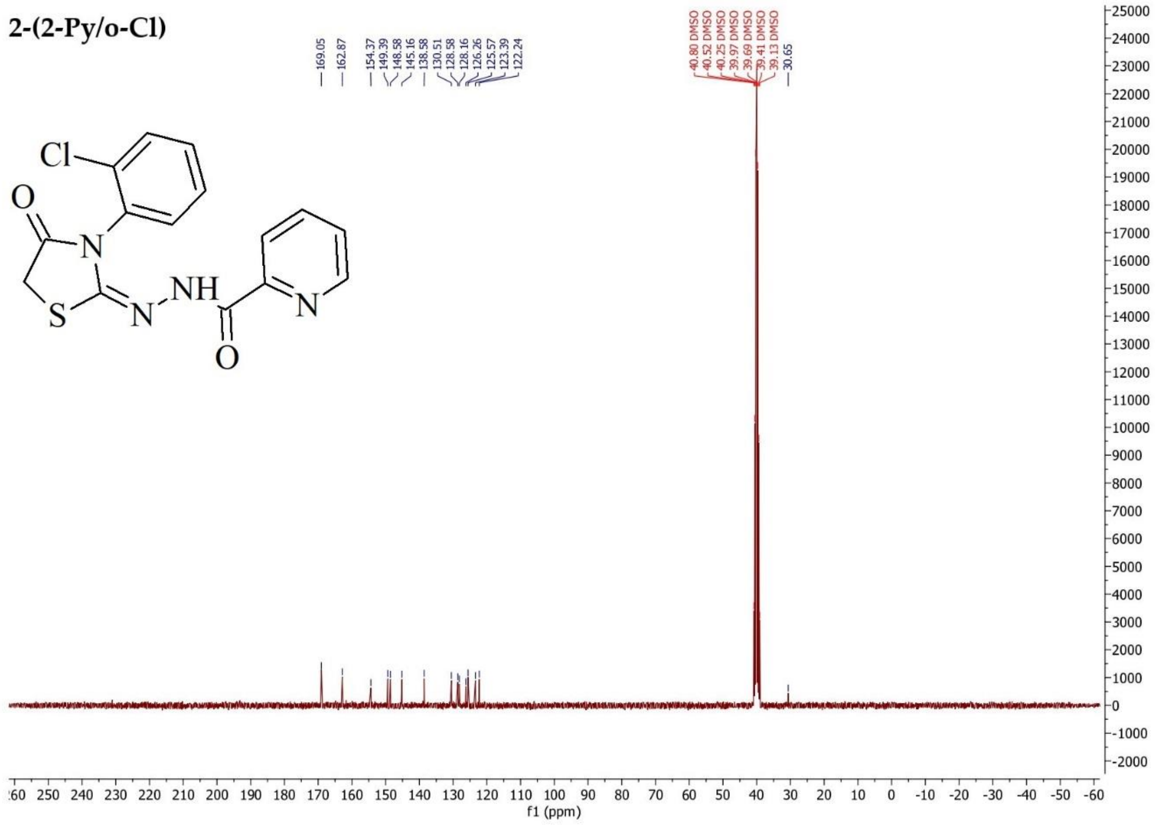

Figure S2. The ^{13}C NMR spectrum of compound **2-(2-Py/*o*-Cl)**

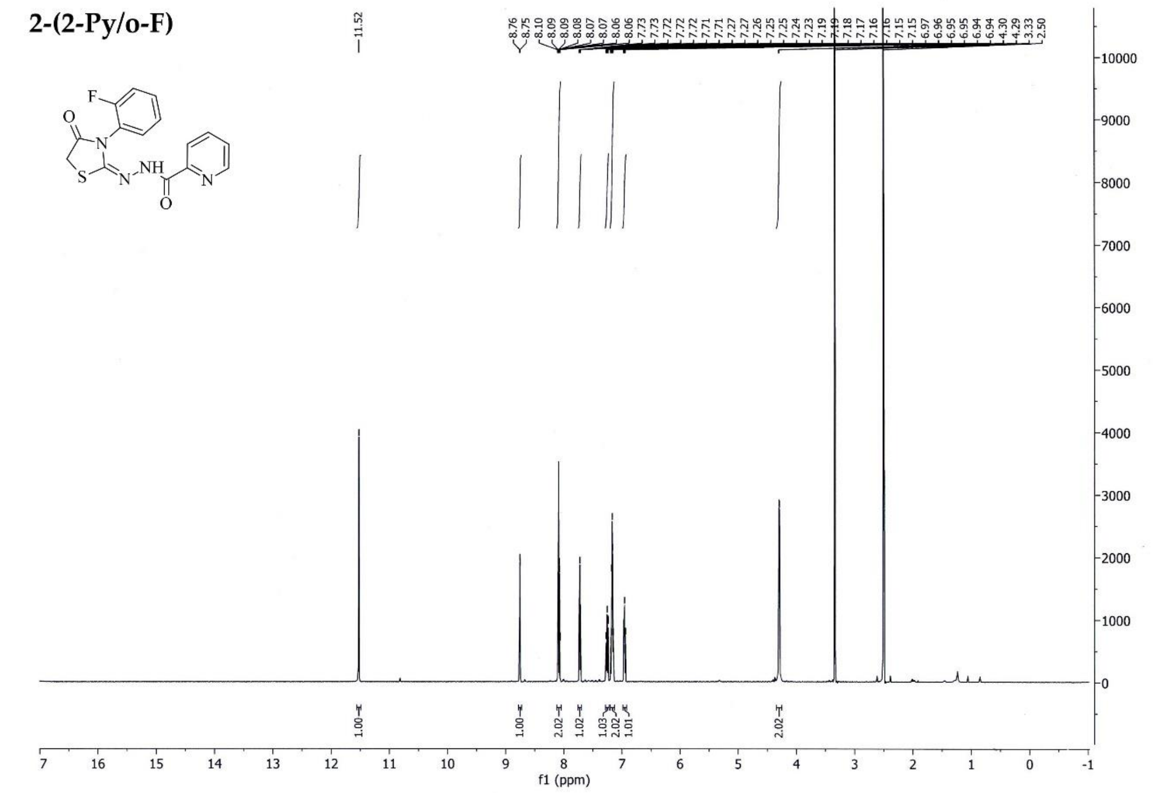

Figure S3. The ^{1}H NMR spectrum of compound **2-(2-Py/*o*-F)**

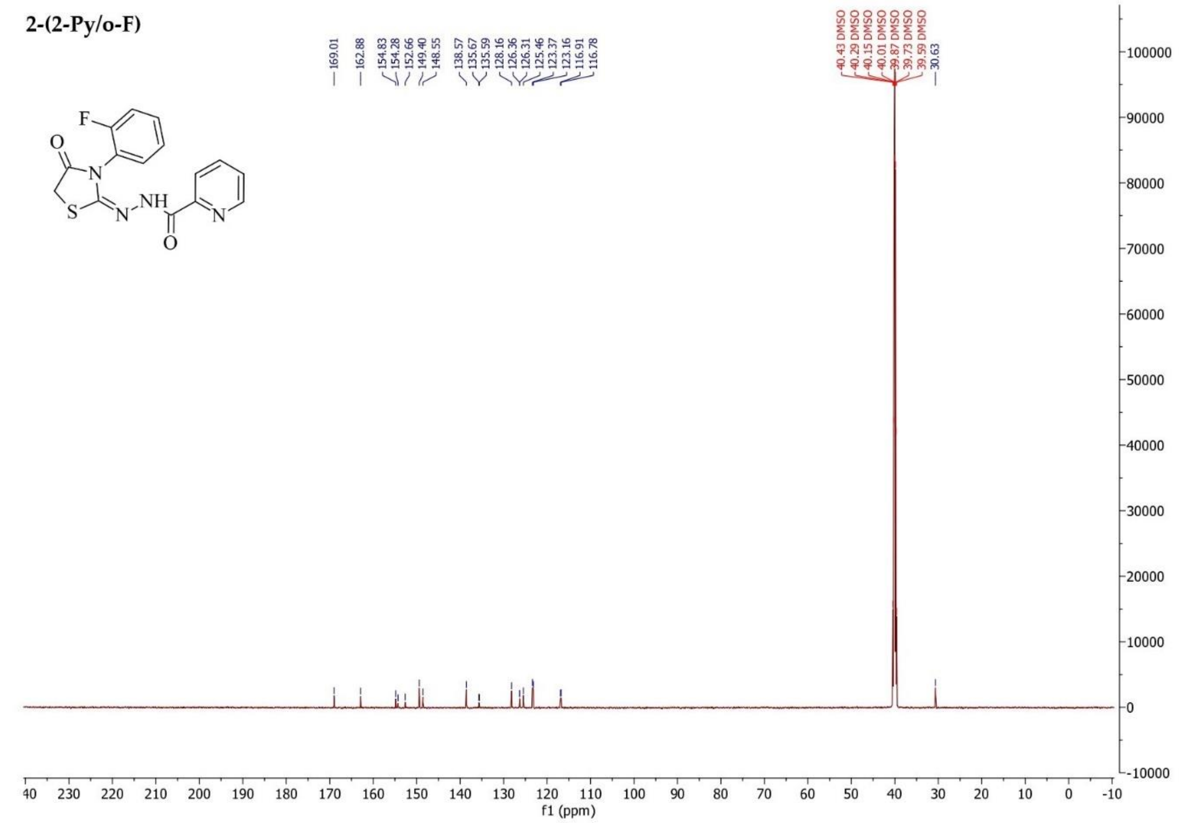

Figure S4. The ^{13}C NMR spectrum of compound **2-(2-Py/*o*-F)**

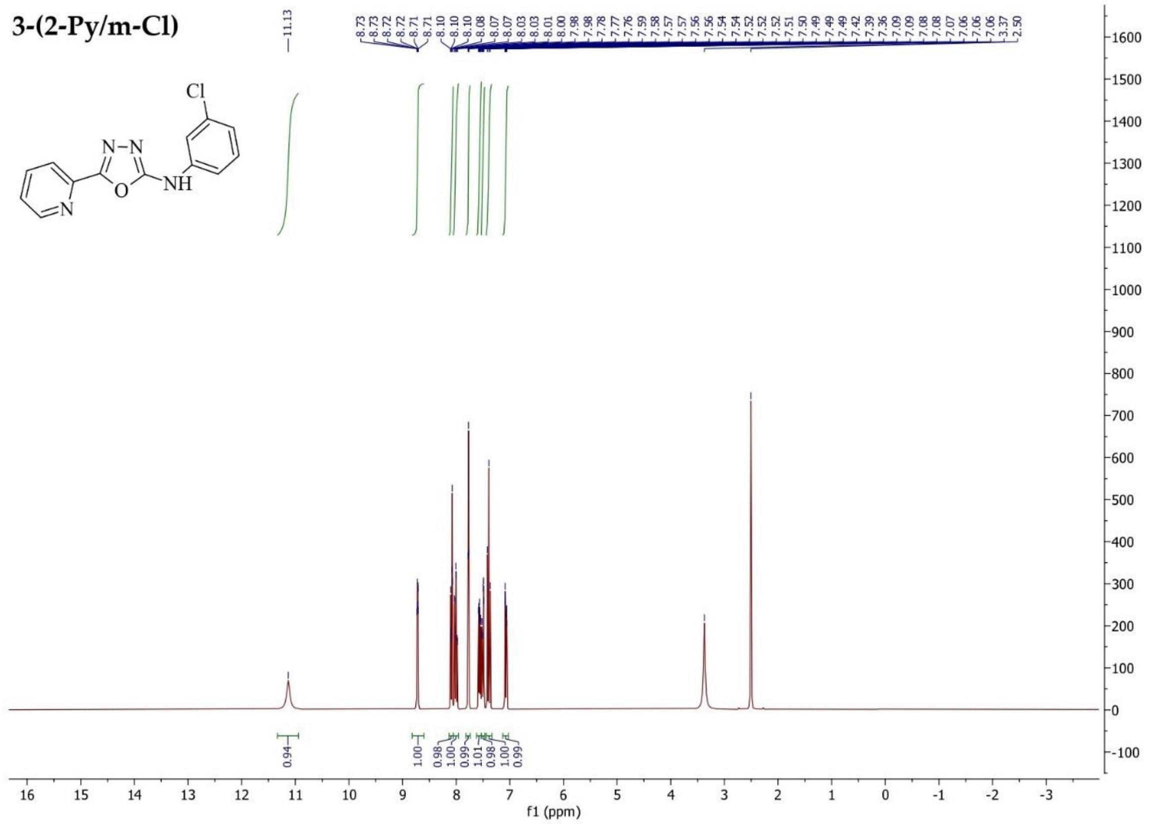

Figure S5. The ^{1}H NMR spectrum of compound **3-(2-Py/*m*-Cl)**

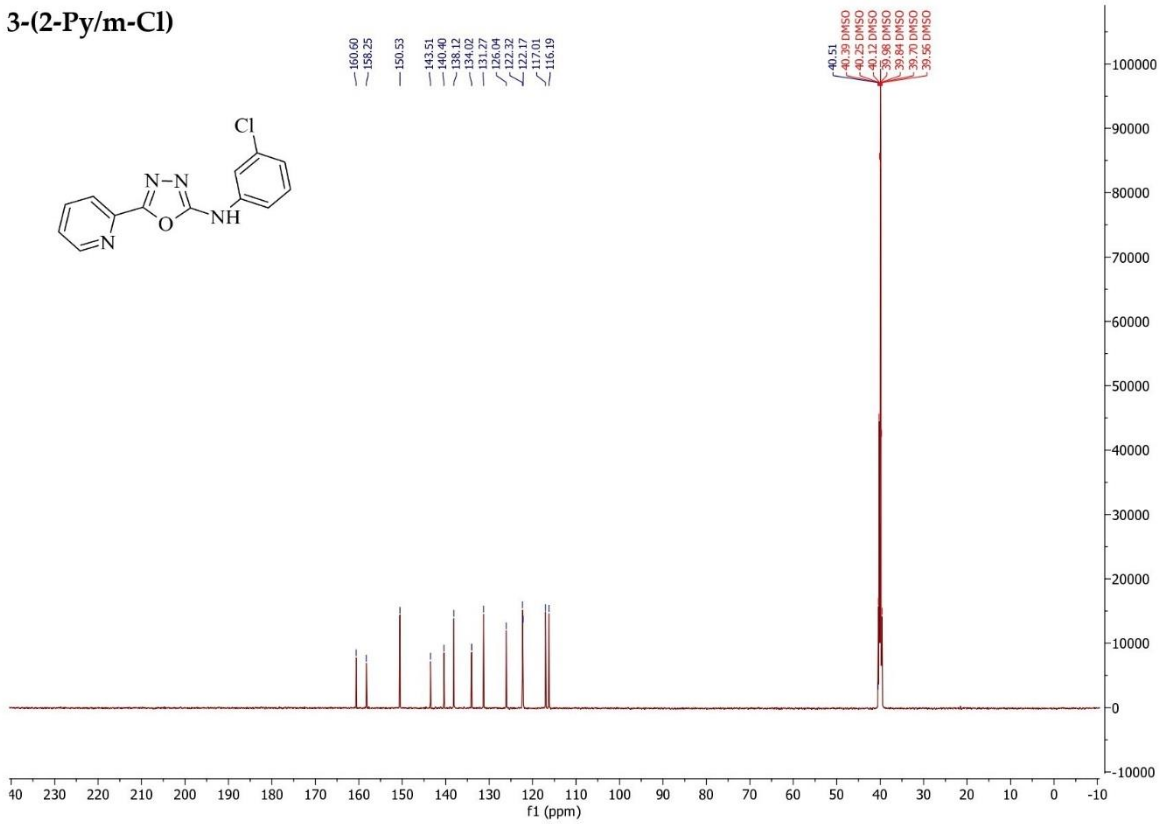

Figure S6. The ^{13}C NMR spectrum of compound **3-(2-Py/*m*-Cl)**

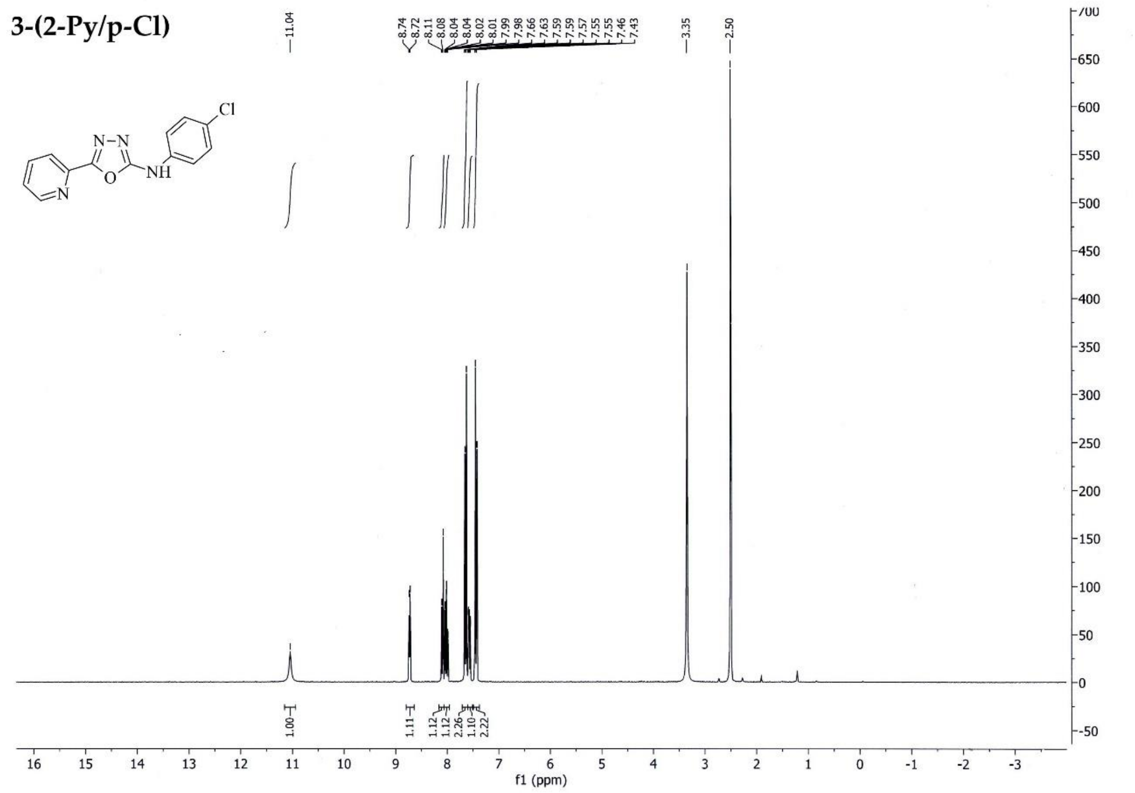

Figure S7. The ^{1}H NMR spectrum of compound **3-(2-Py/*p*-Cl)**

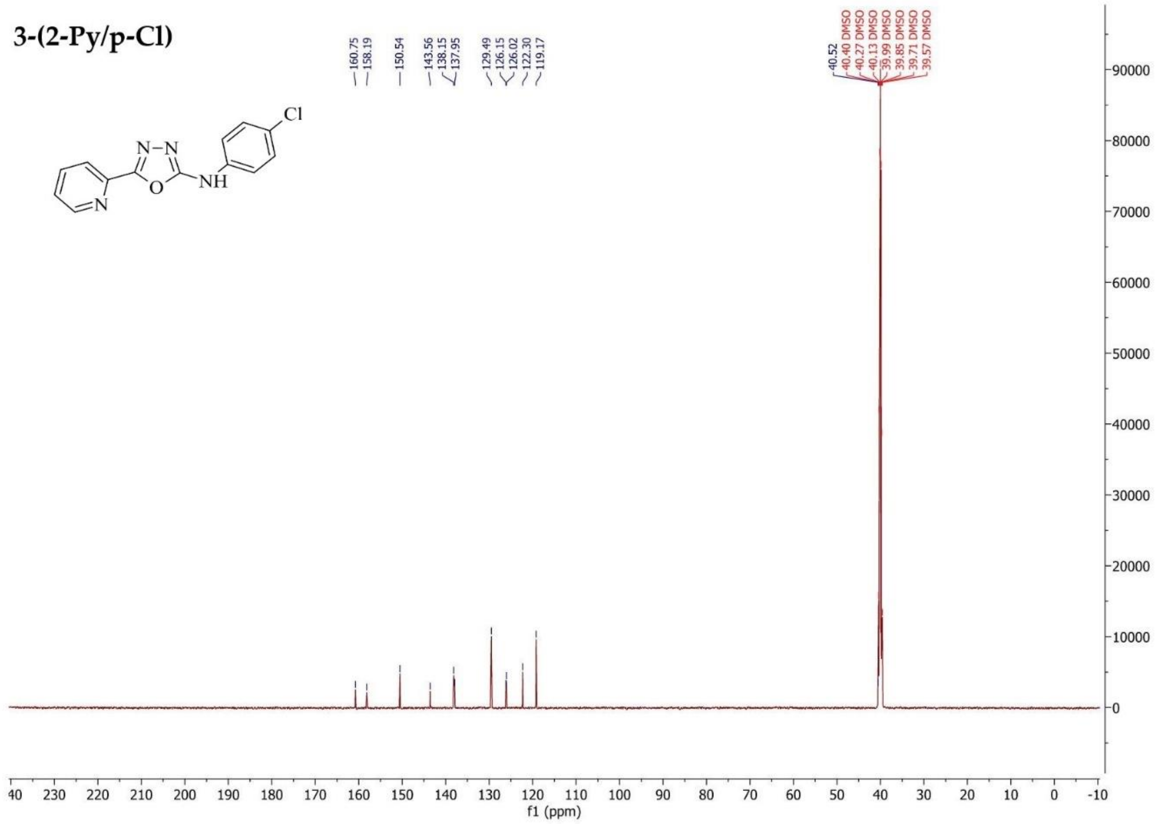

Figure S8. The ^{13}C NMR spectrum of compound **3-(2-Py/*p*-Cl)**

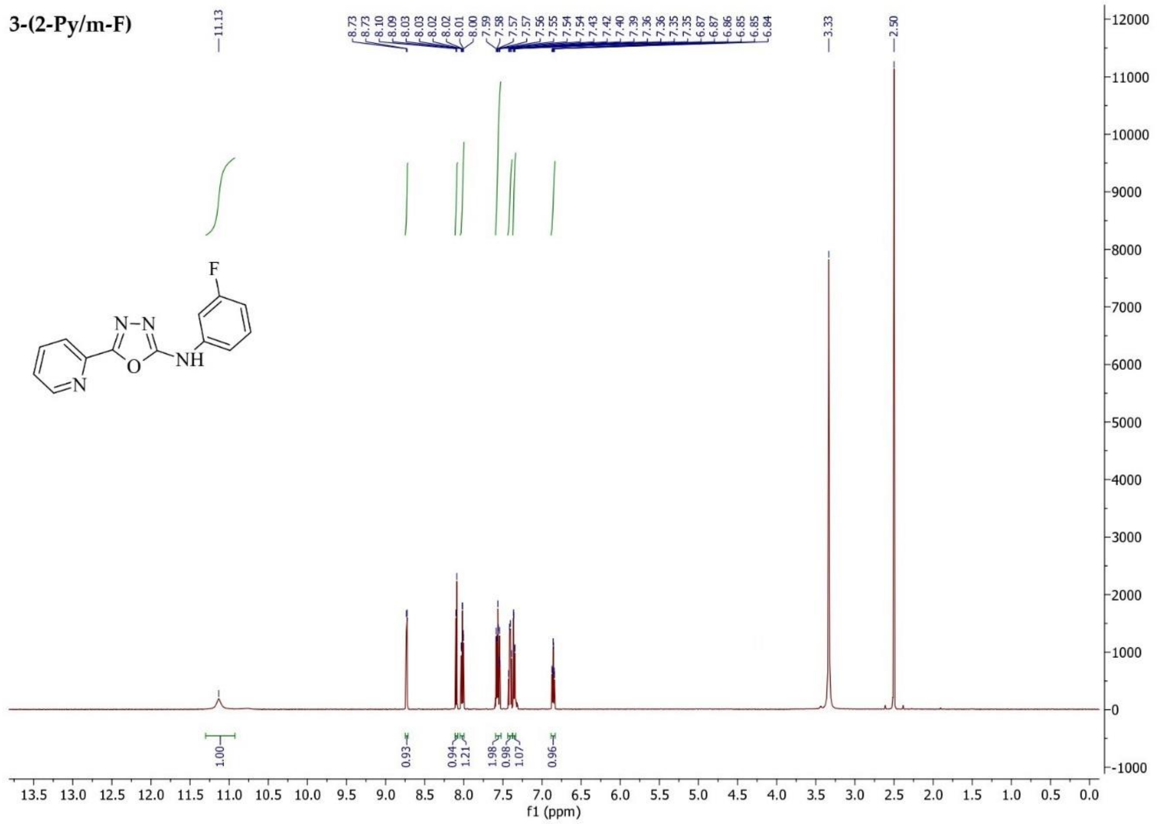

Figure S9. The ^{1}H NMR spectrum of compound **3-(2-Py/*m*-F)**

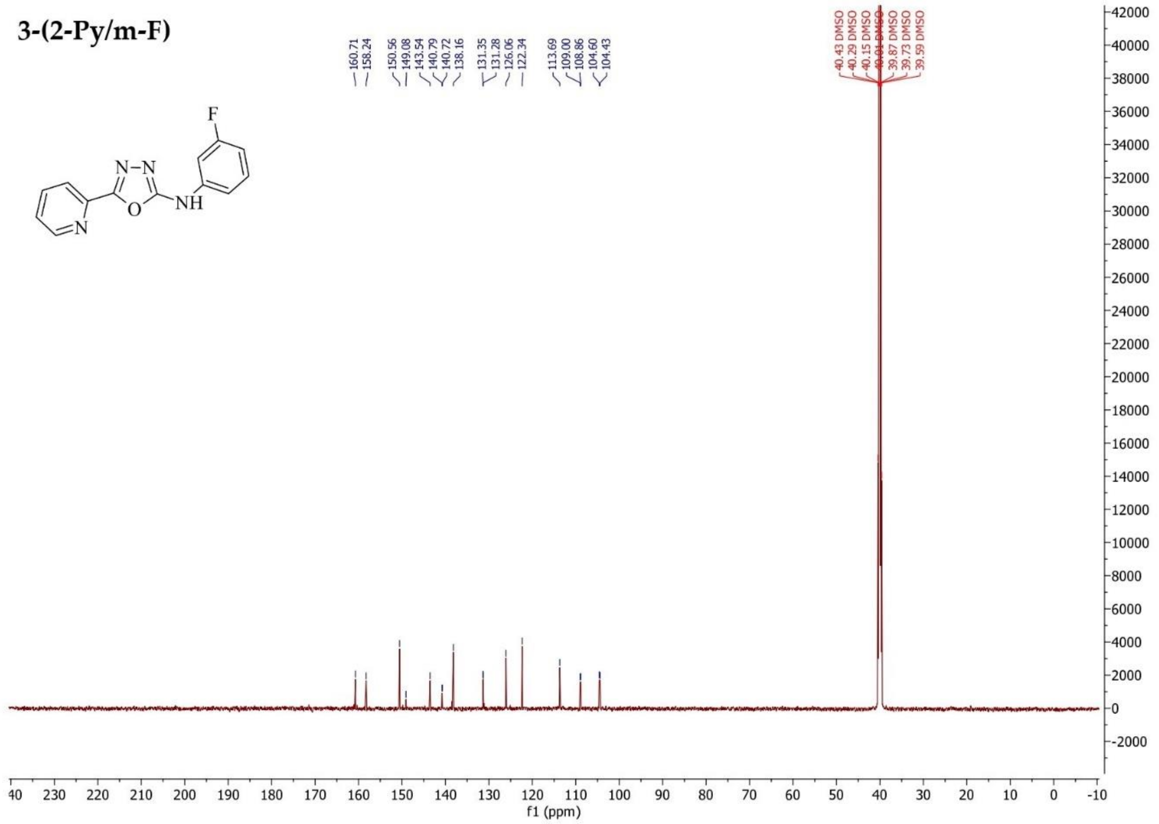

Figure S10. The ^{13}C NMR spectrum of compound **3-(2-Py/*m*-F)**

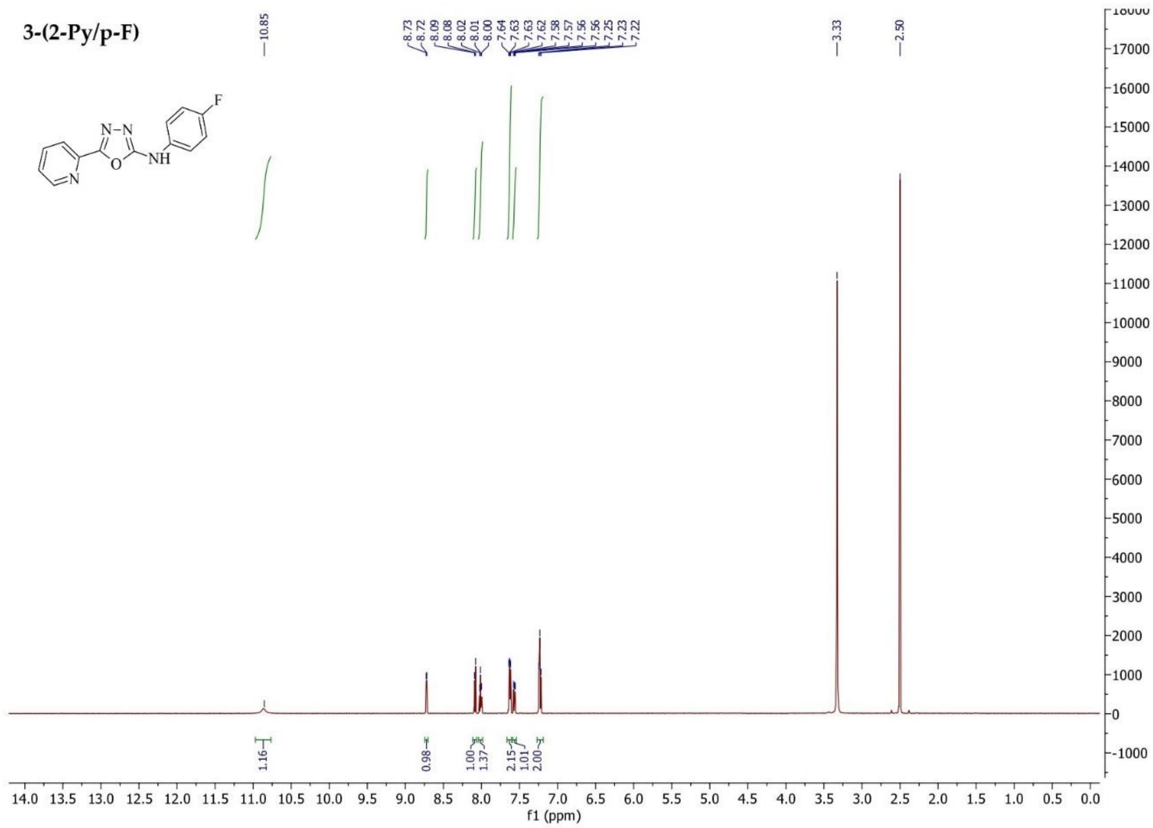

Figure S11. The ^{1}H NMR spectrum of compound **3-(2-Py/*p*-F)**

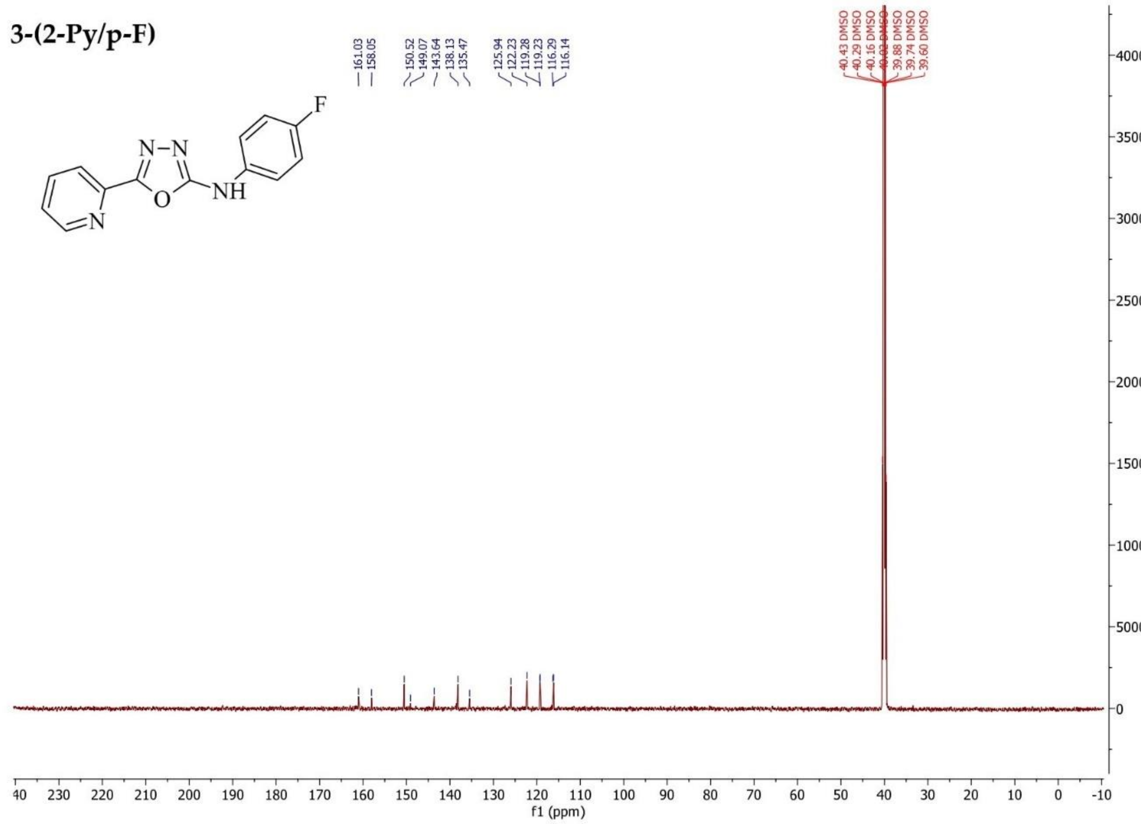

Figure S12. The ^{13}C NMR spectrum of compound **3-(2-Py/*p*-F)**

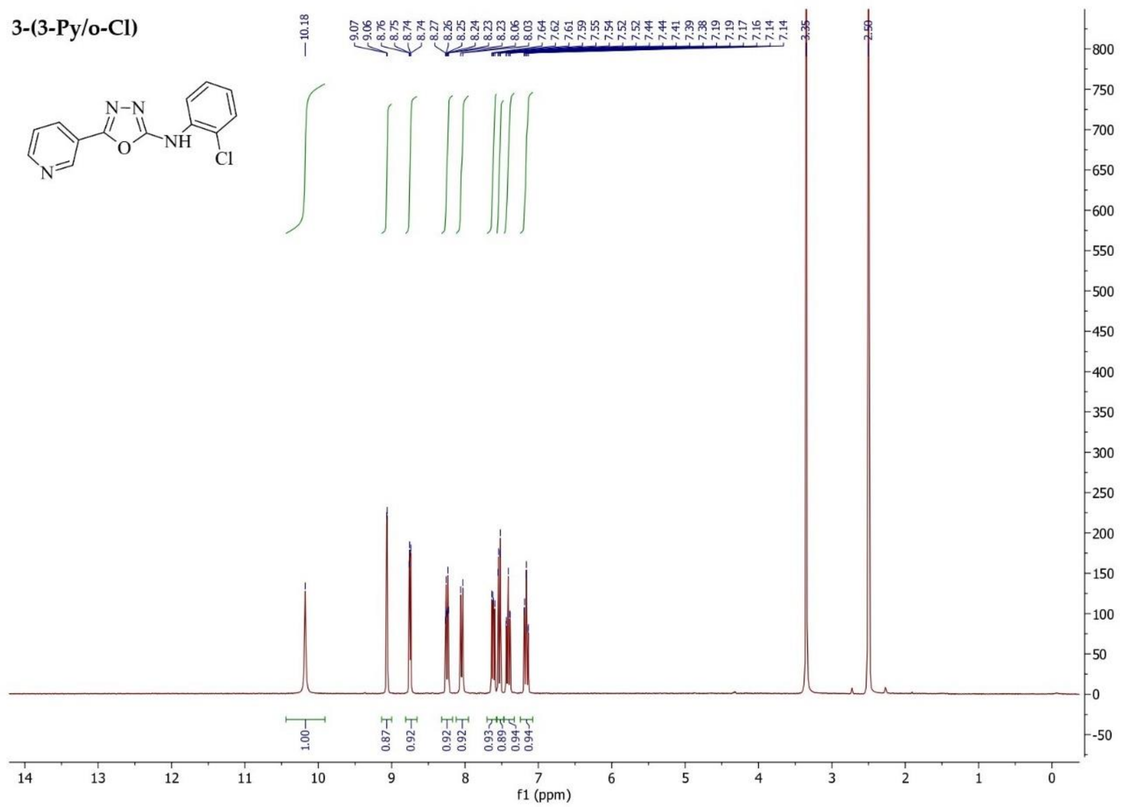

Figure S13. The ^{1}H NMR spectrum of compound **3-(3-Py/*o*-Cl)**

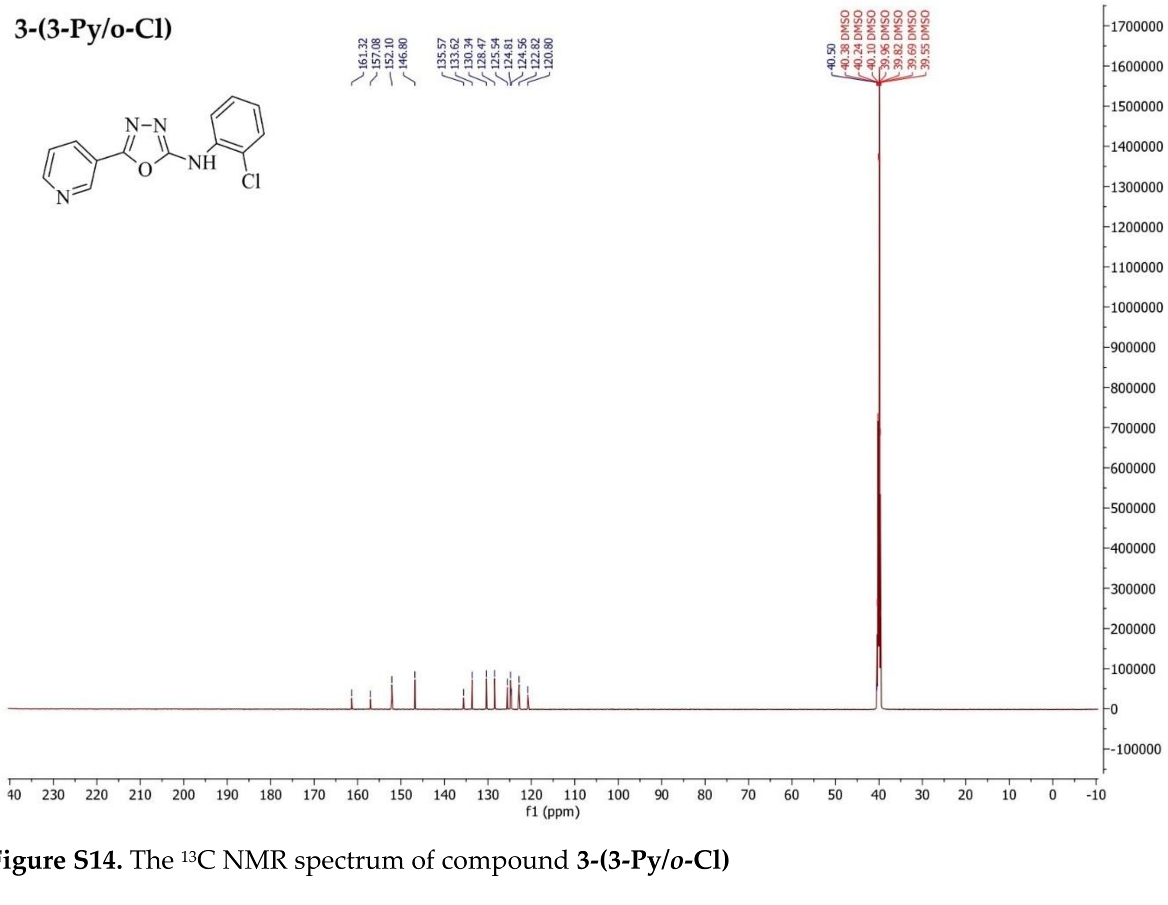

Figure S14. The ^{13}C NMR spectrum of compound **3-(3-Py/*o*-Cl)**

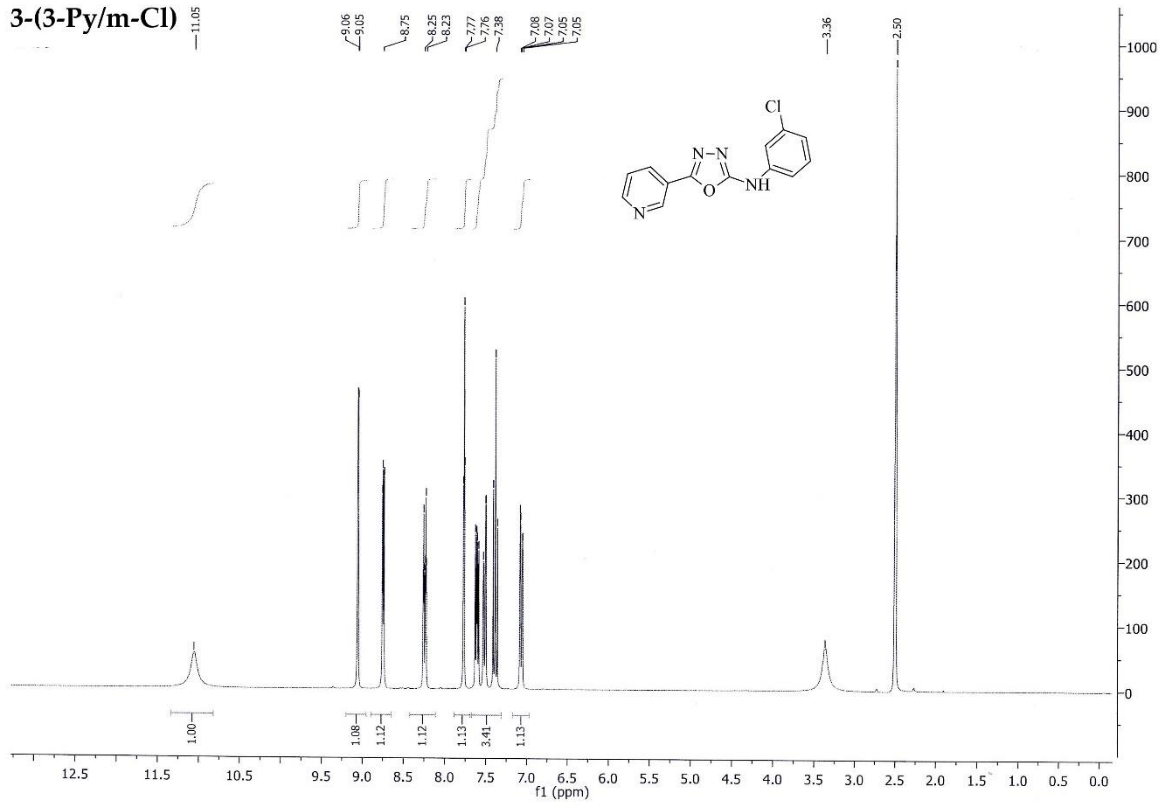

Figure S15. The ^{1}H NMR spectrum of compound **3-(3-Py/*m*-Cl)**

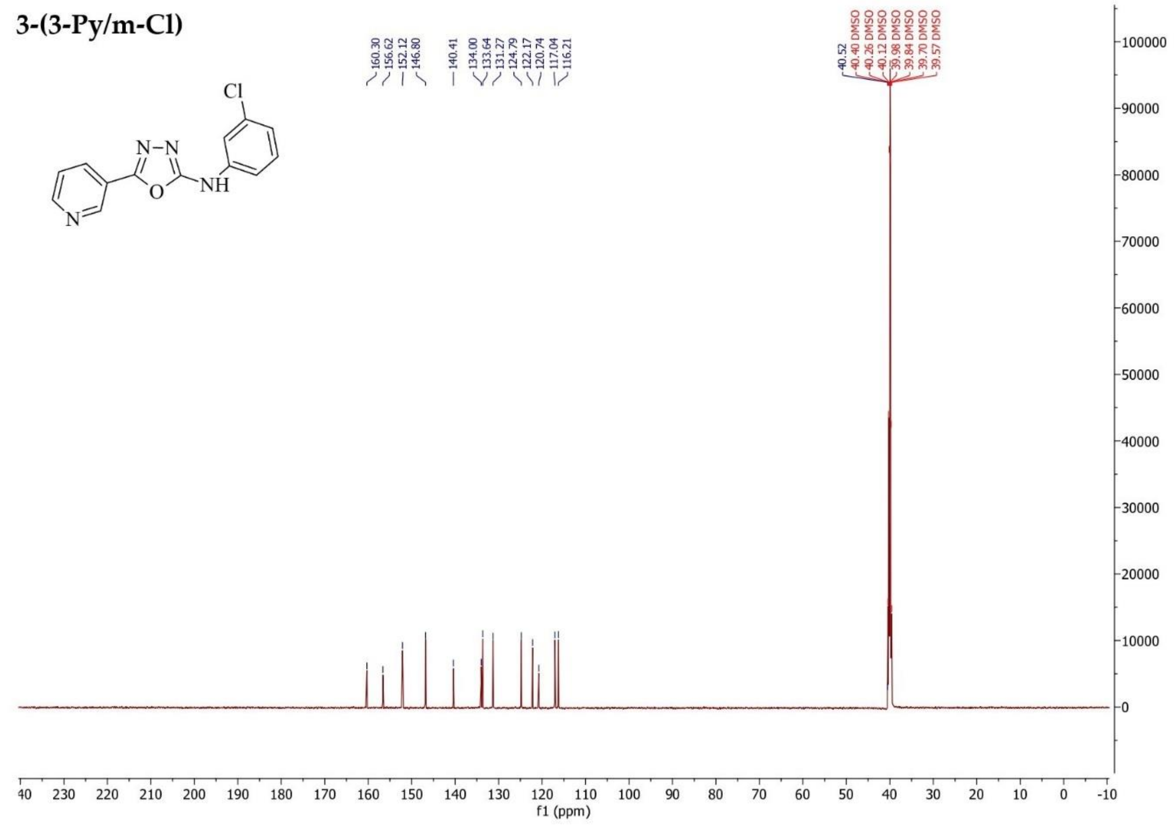

Figure S16. The ^{13}C NMR spectrum of compound **3-(3-Py/*m*-Cl)**

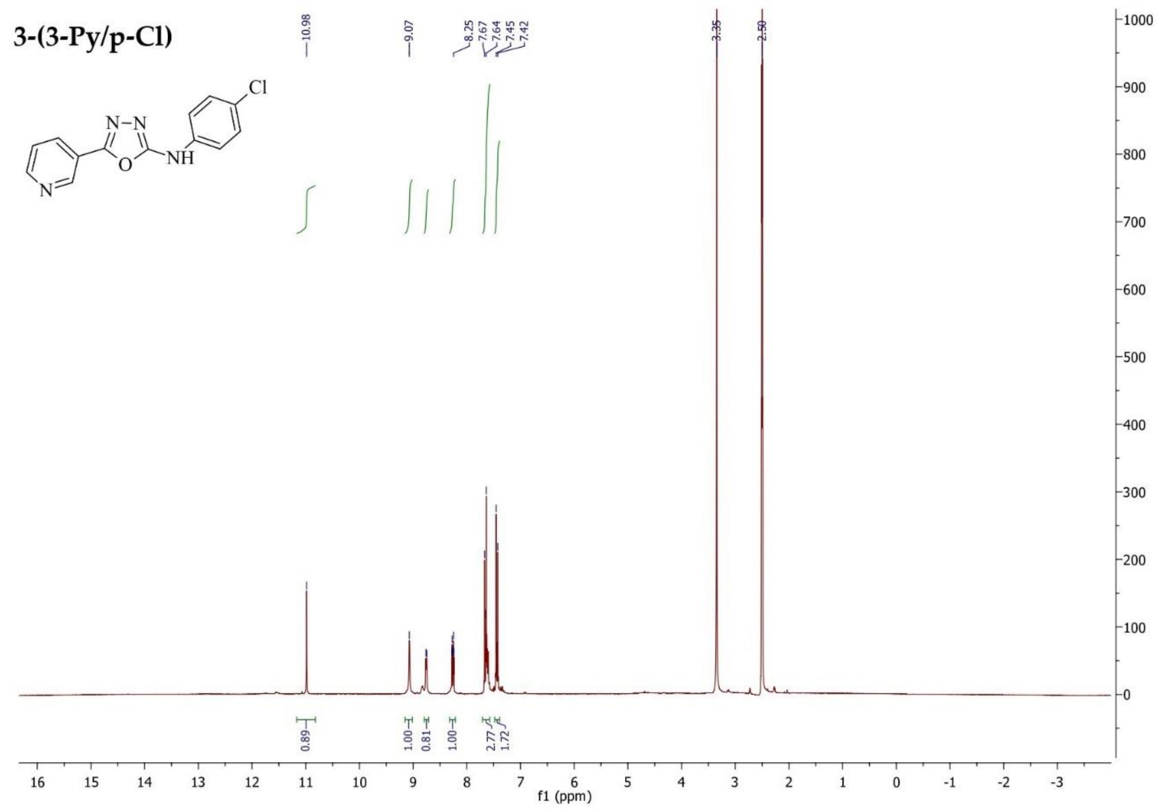

Figure S17. The ^{1}H NMR spectrum of compound **3-(3-Py/*p*-Cl)**

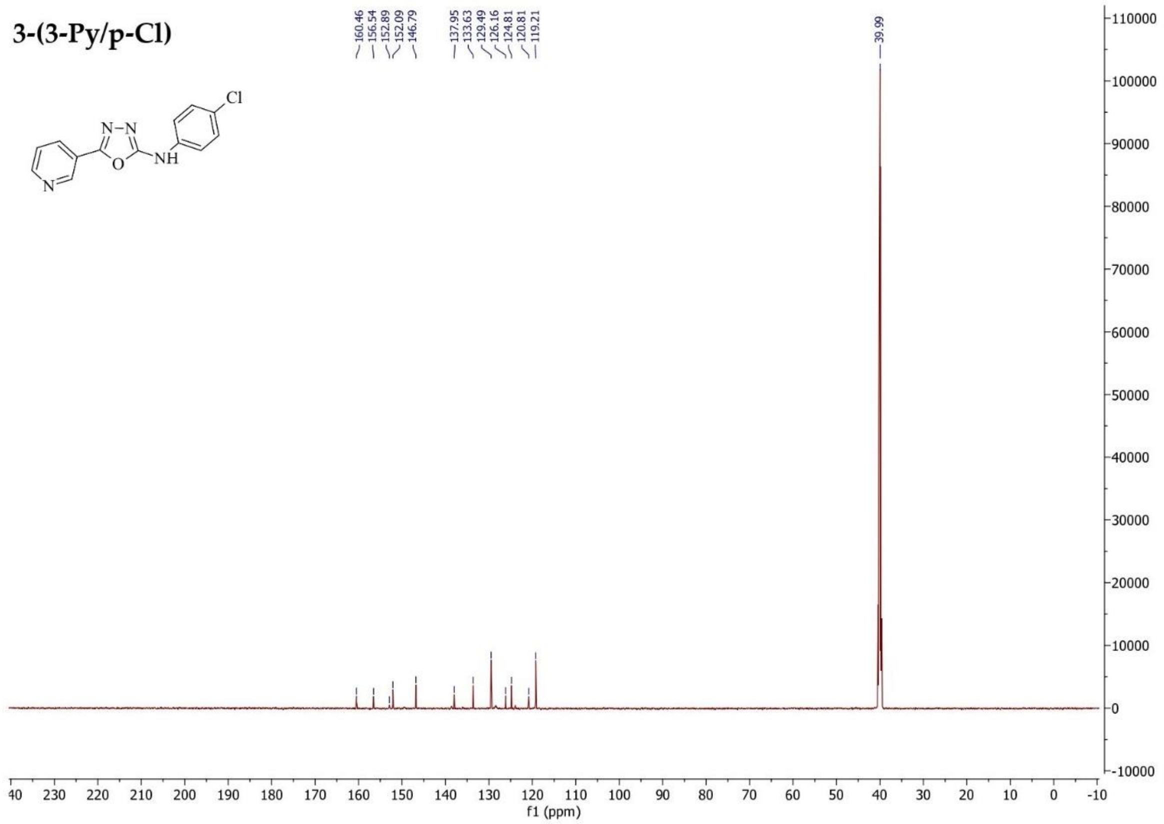

Figure S18. The ^{13}C NMR spectrum of compound **3-(3-Py/*p*-Cl)**

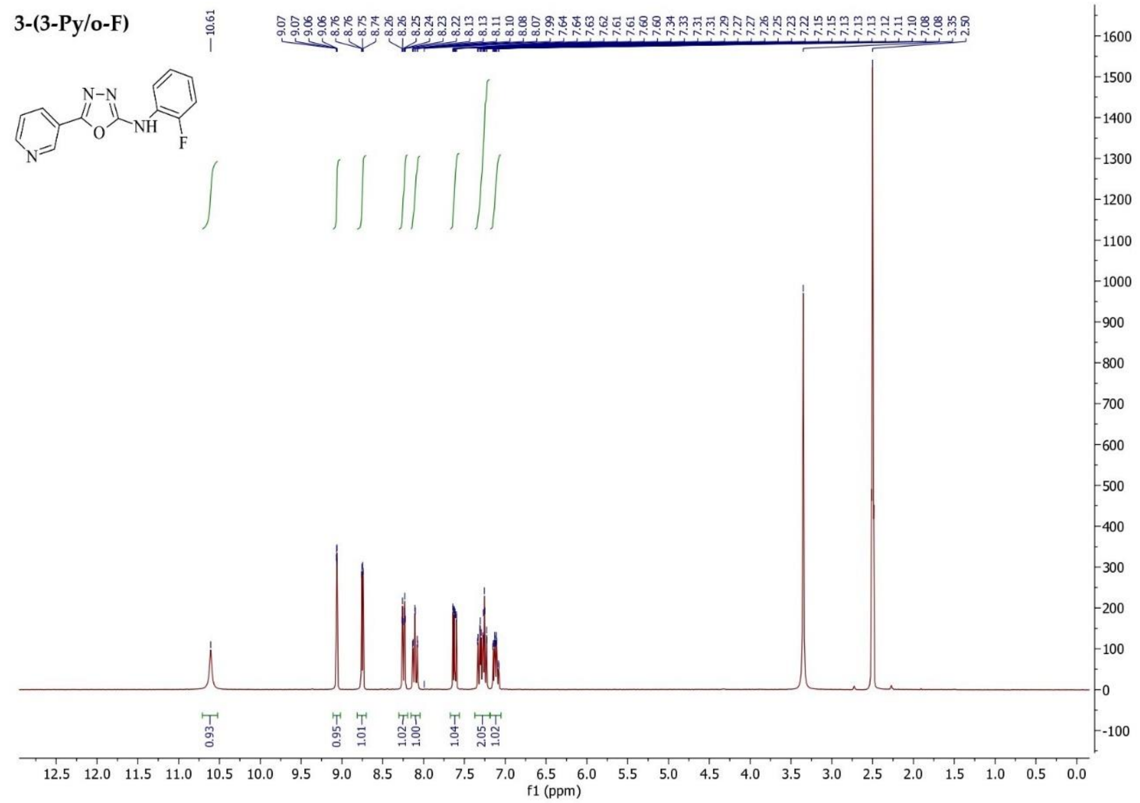

Figure S19. The ^{1}H NMR spectrum of compound **3-(3-Py/*o*-F)**

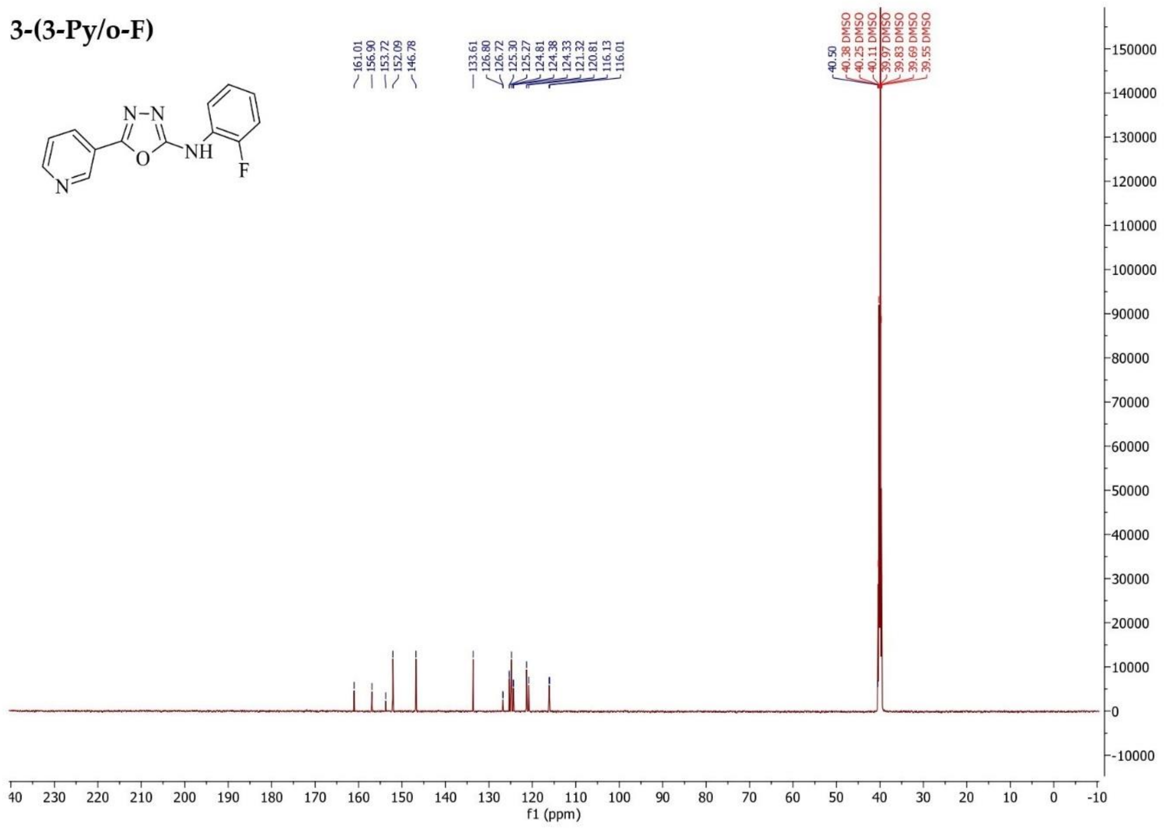

Figure S20. The ^{13}C NMR spectrum of compound **3-(3-Py/*o*-F)**

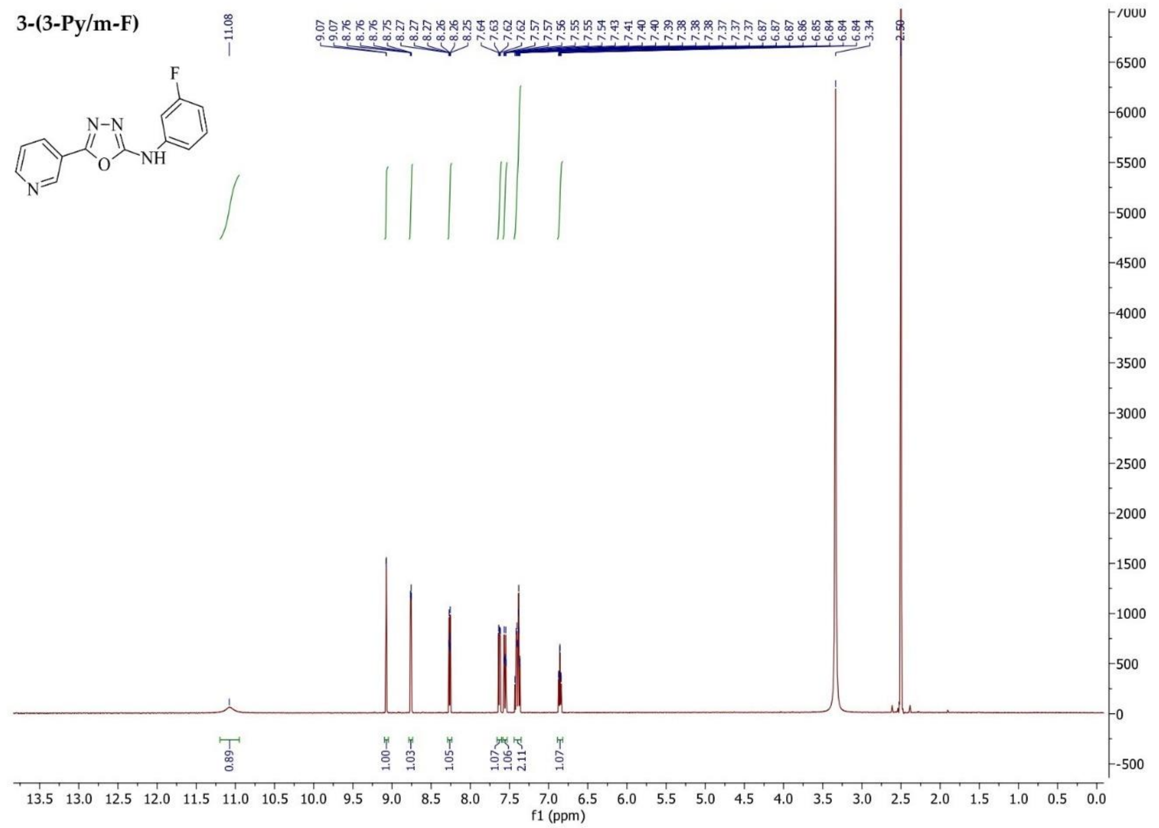

Figure S21. The ^{1}H NMR spectrum of compound **3-(3-Py/*m*-F)**

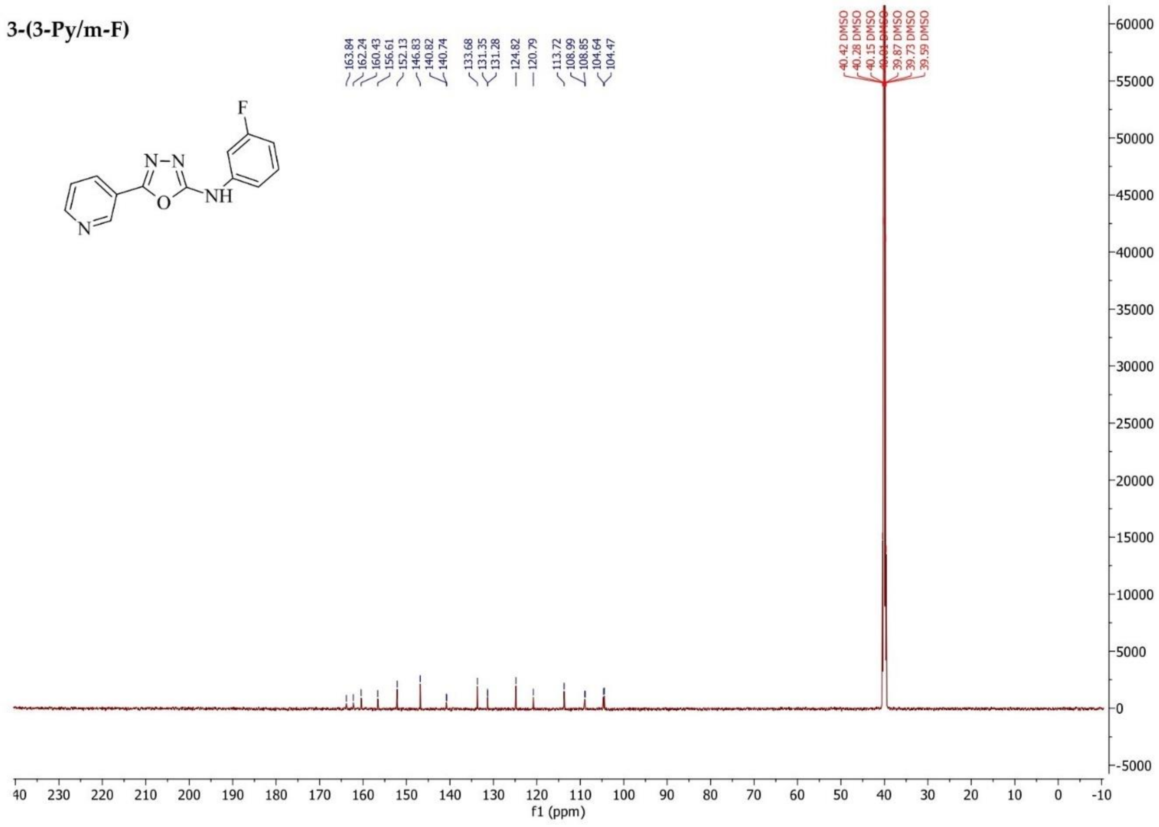

Figure S22. The ^{13}C NMR spectrum of compound **3-(3-Py/*m*-F)**

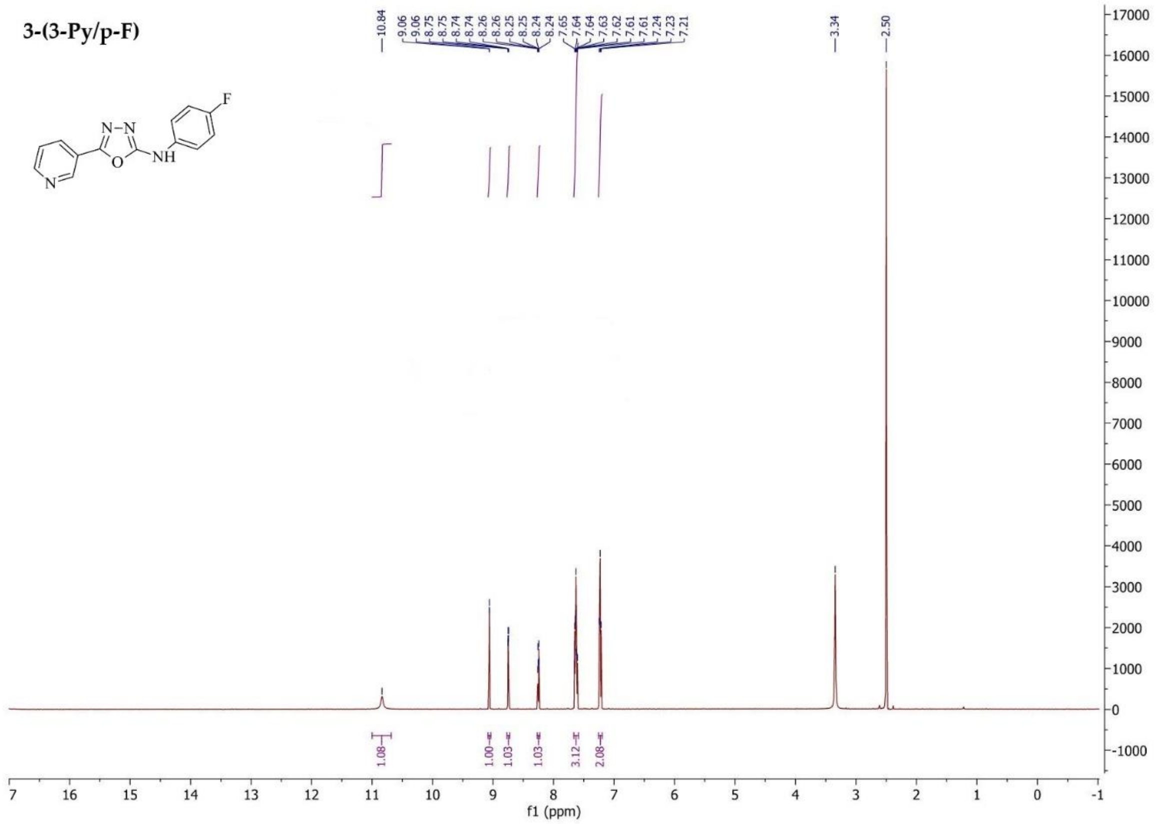

Figure S23. The ^{1}H NMR spectrum of compound **3-(3-Py/*p*-F)**

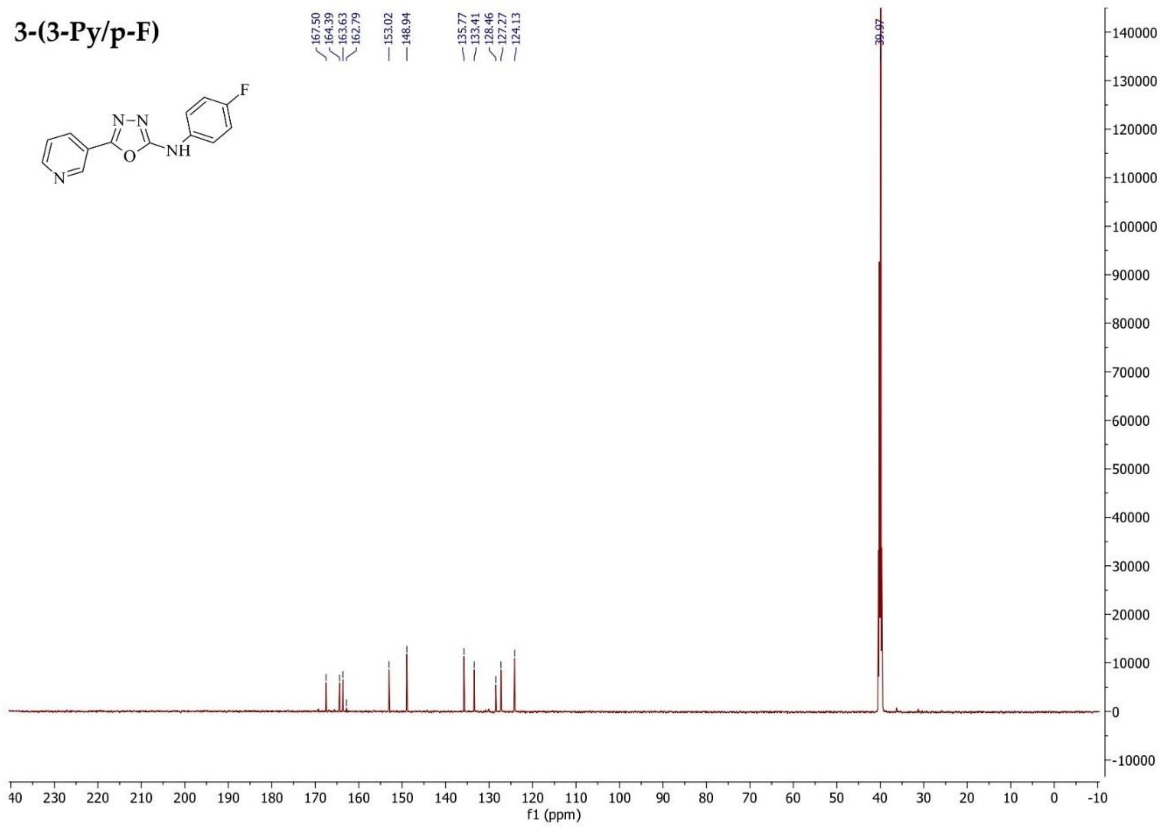

Figure S24. The ^{13}C NMR spectrum of compound **3-(3-Py/*p*-F)**

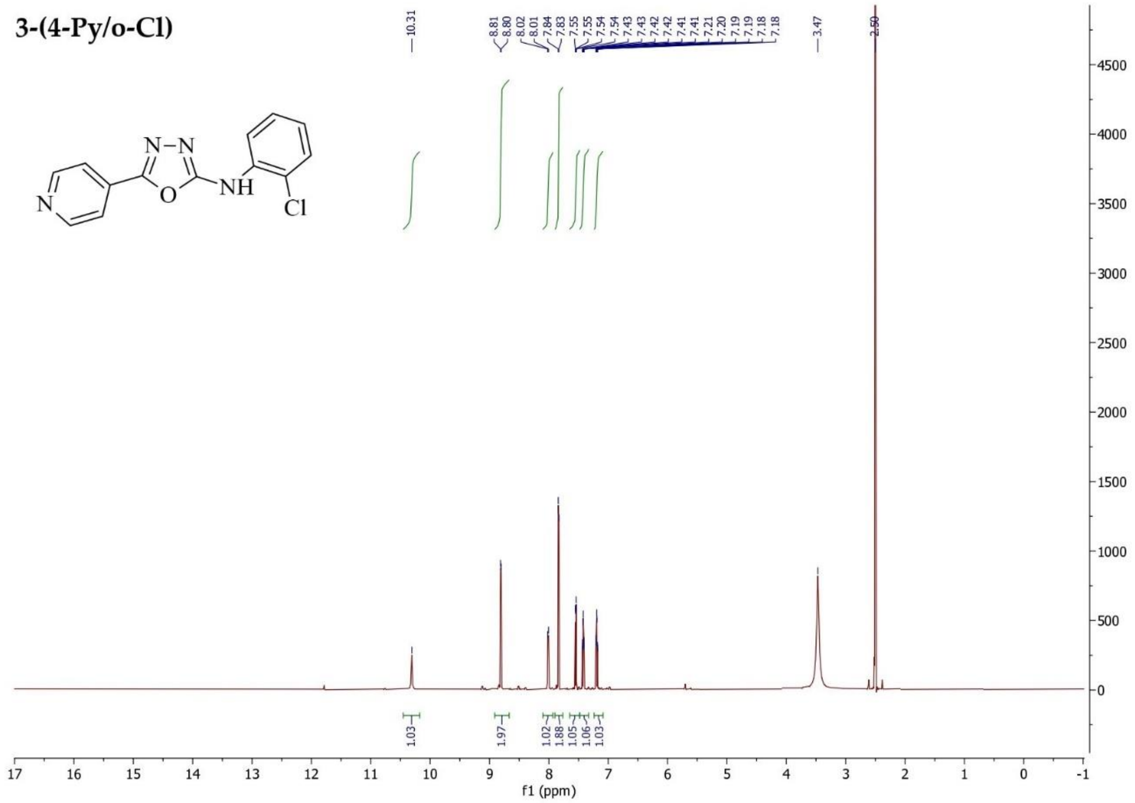

Figure S25. The ^{1}H NMR spectrum of compound **3-(4-Py/*o*-Cl)**

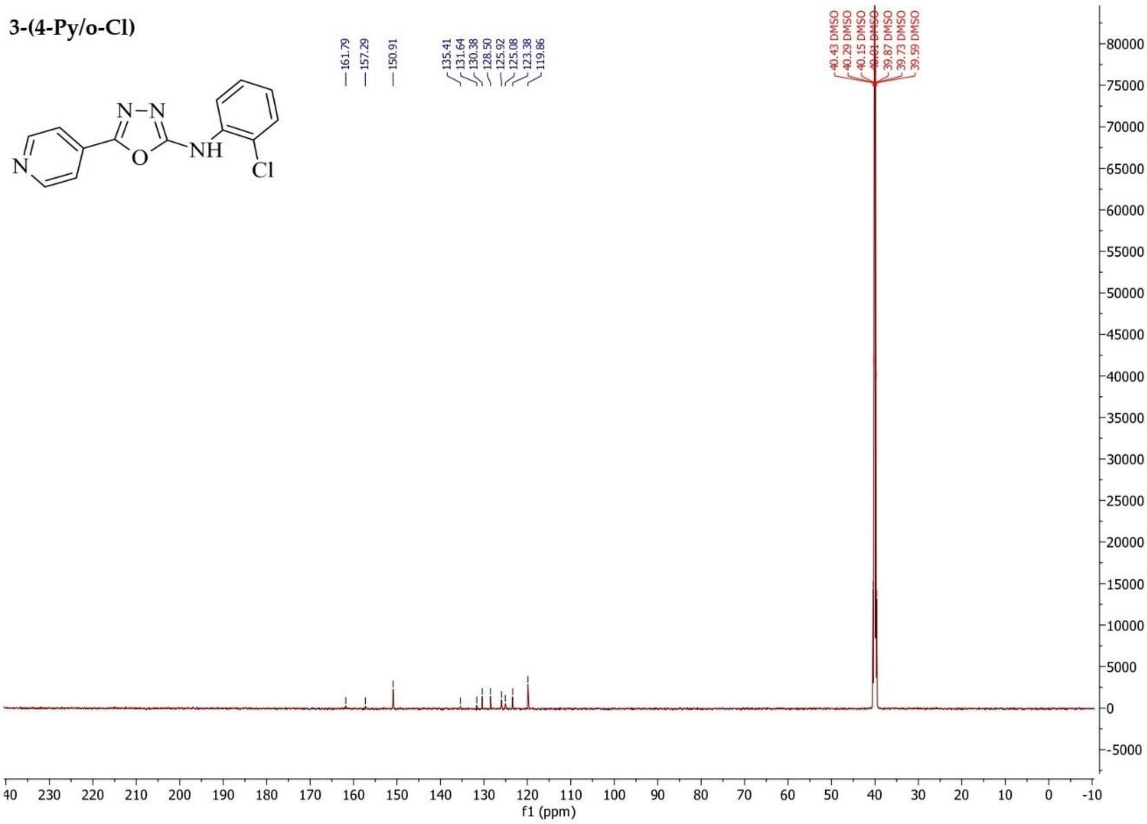

Figure S26. The ^{13}C NMR spectrum of compound **3-(4-Py/*o*-Cl)**

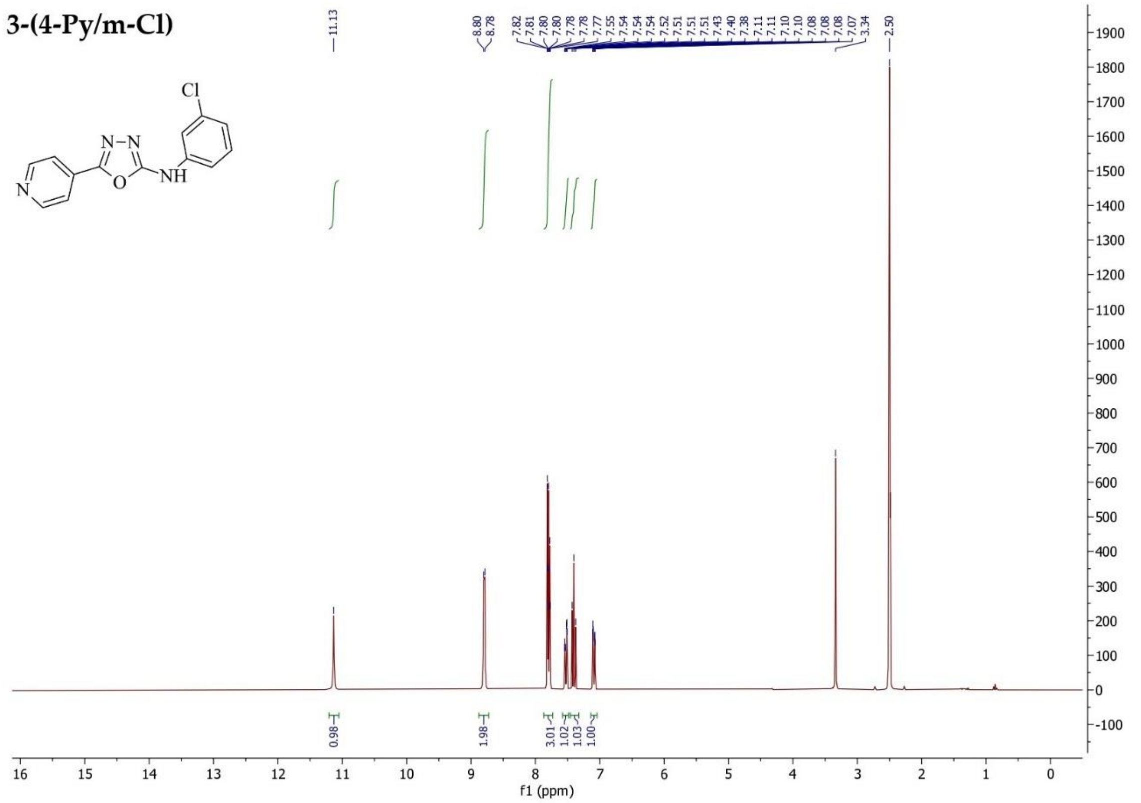

Figure S27. The ^{1}H NMR spectrum of compound **3-(4-Py/*m*-Cl)**

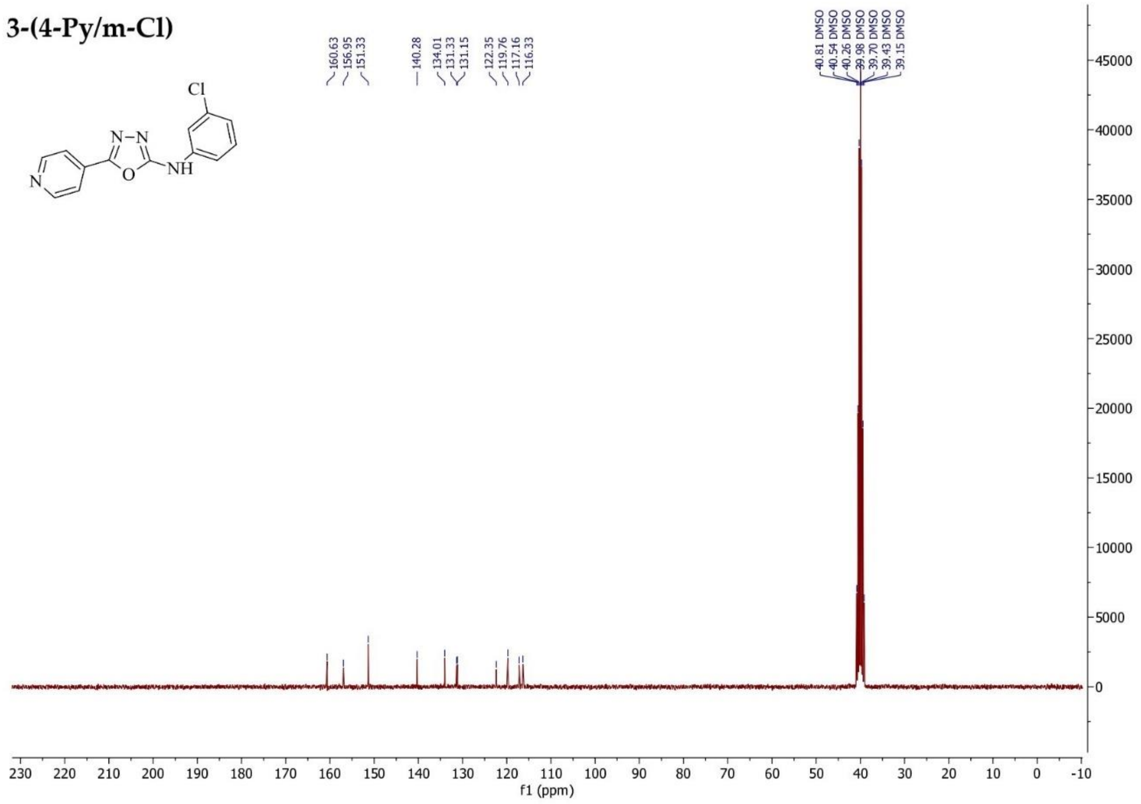

Figure S28. The ^{13}C NMR spectrum of compound **3-(4-Py/*m*-Cl)**

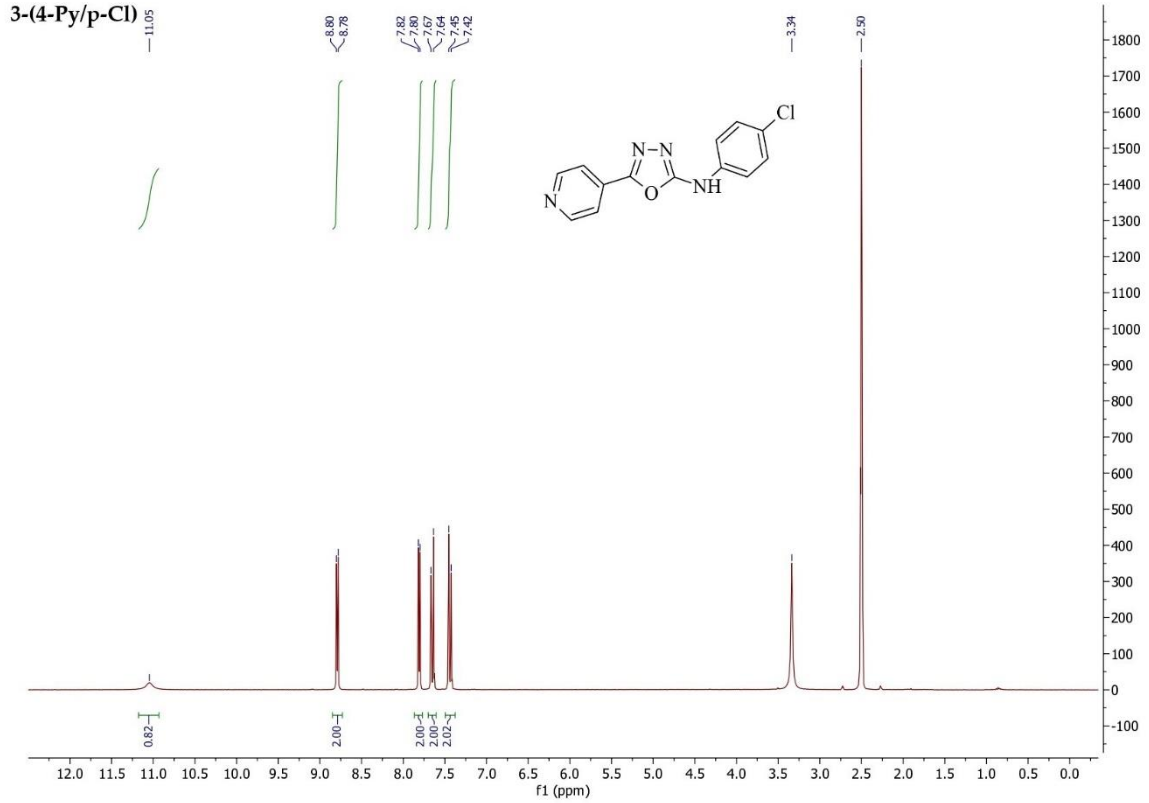

Figure S29. The ^{1}H NMR spectrum of compound **3-(4-Py/*p*-Cl)**

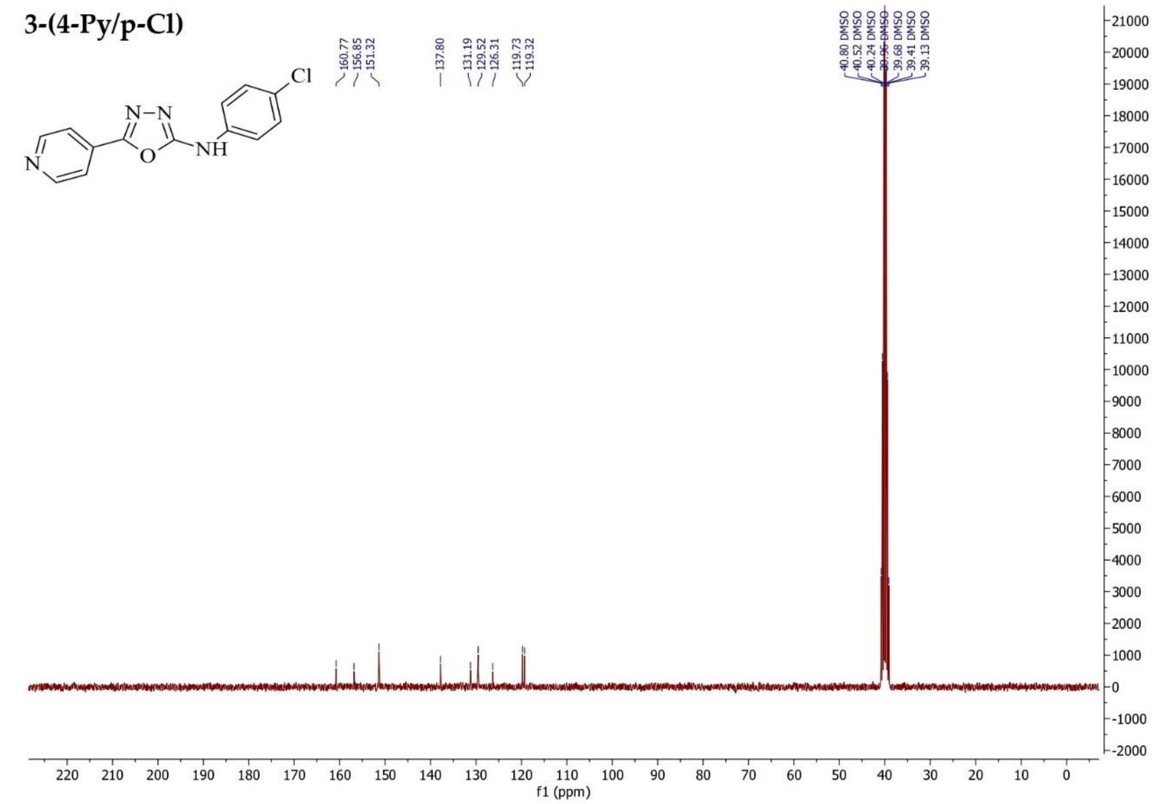

Figure S30. The ^{13}C NMR spectrum of compound **3-(4-Py/*p*-Cl)**

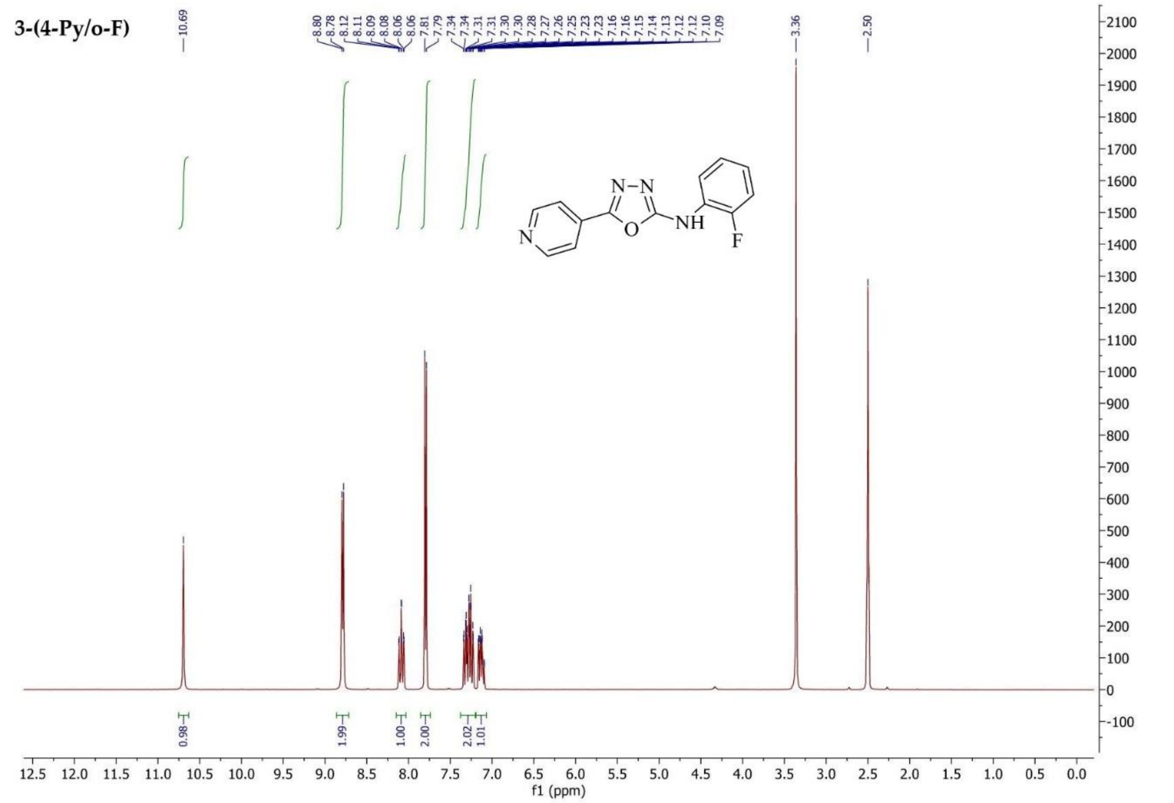

Figure S31. The ^{1}H NMR spectrum of compound **3-(4-Py/*o*-F)**

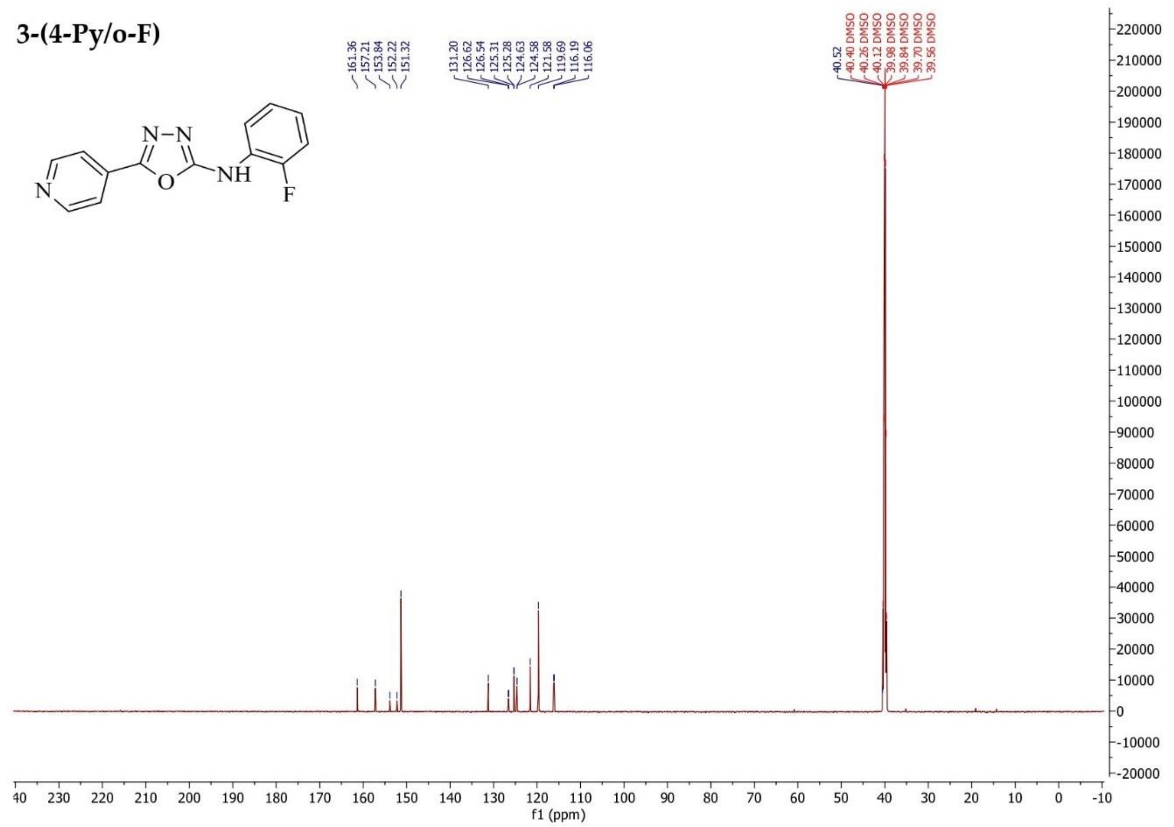

Figure S32. The ^{13}C NMR spectrum of compound **3-(4-Py/*o*-F)**

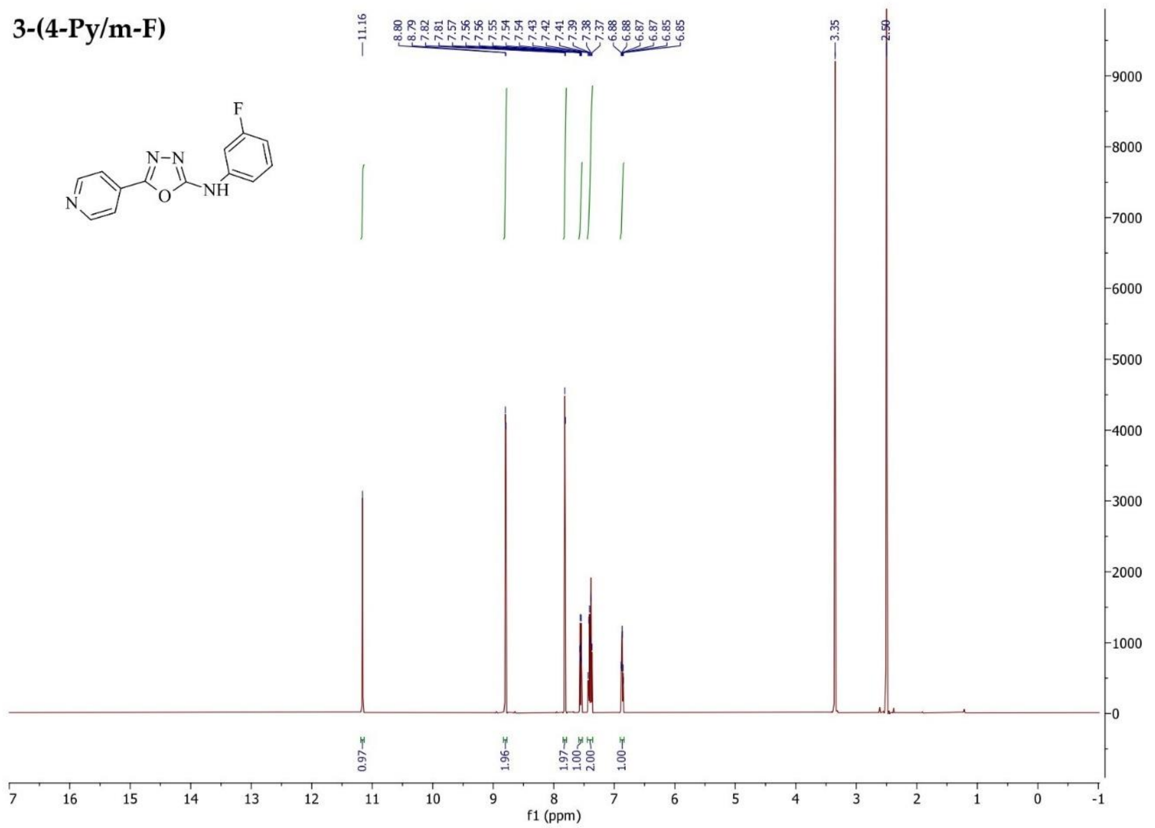

Figure S33. The ^{1}H NMR spectrum of compound **3-(4-Py/*m*-F)**

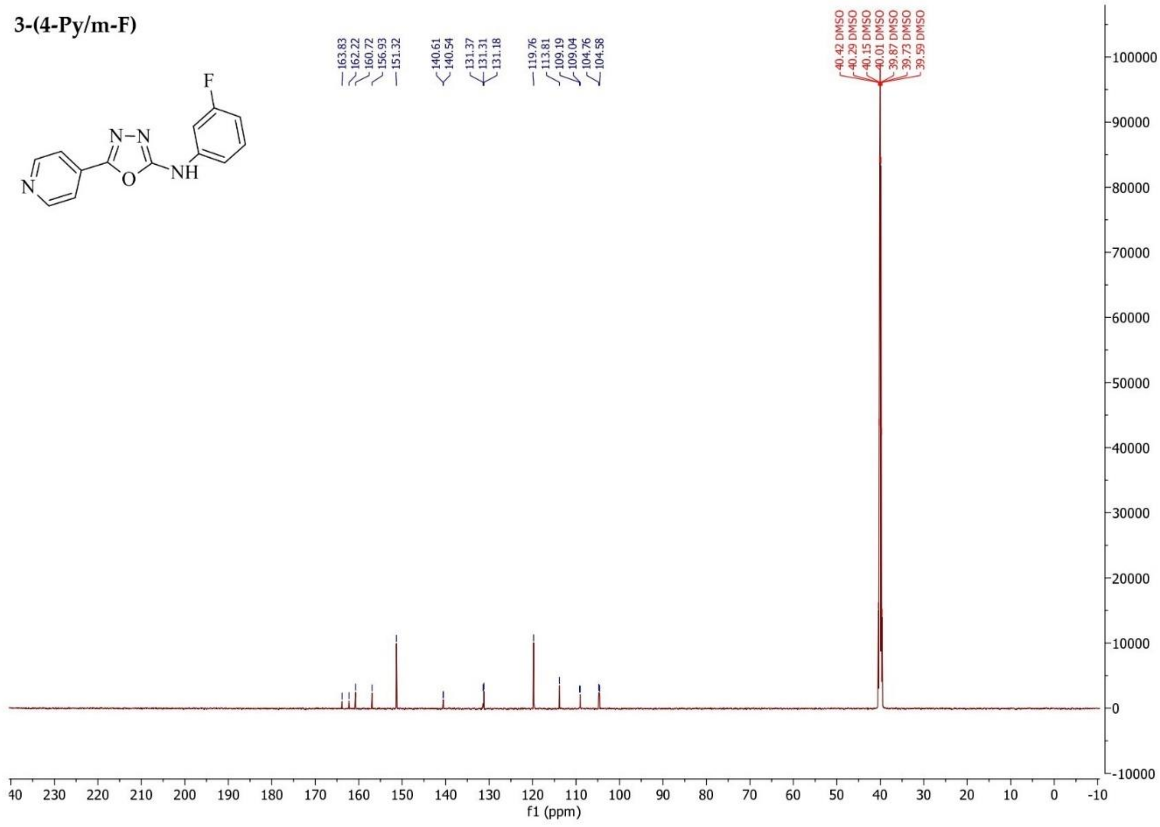

Figure S34. The ^{13}C NMR spectrum of compound **3-(4-Py/*m*-F)**

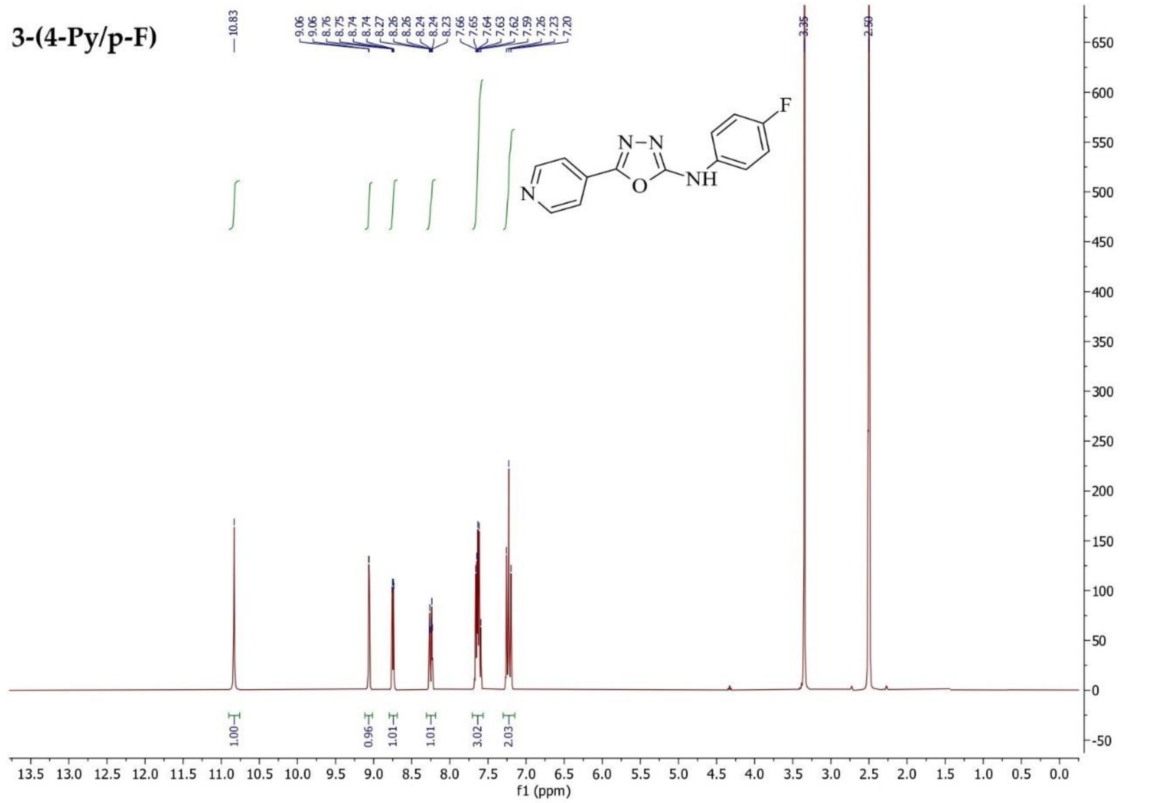

Figure S35. The ^{1}H NMR spectrum of compound **3-(4-Py/*p*-F)**

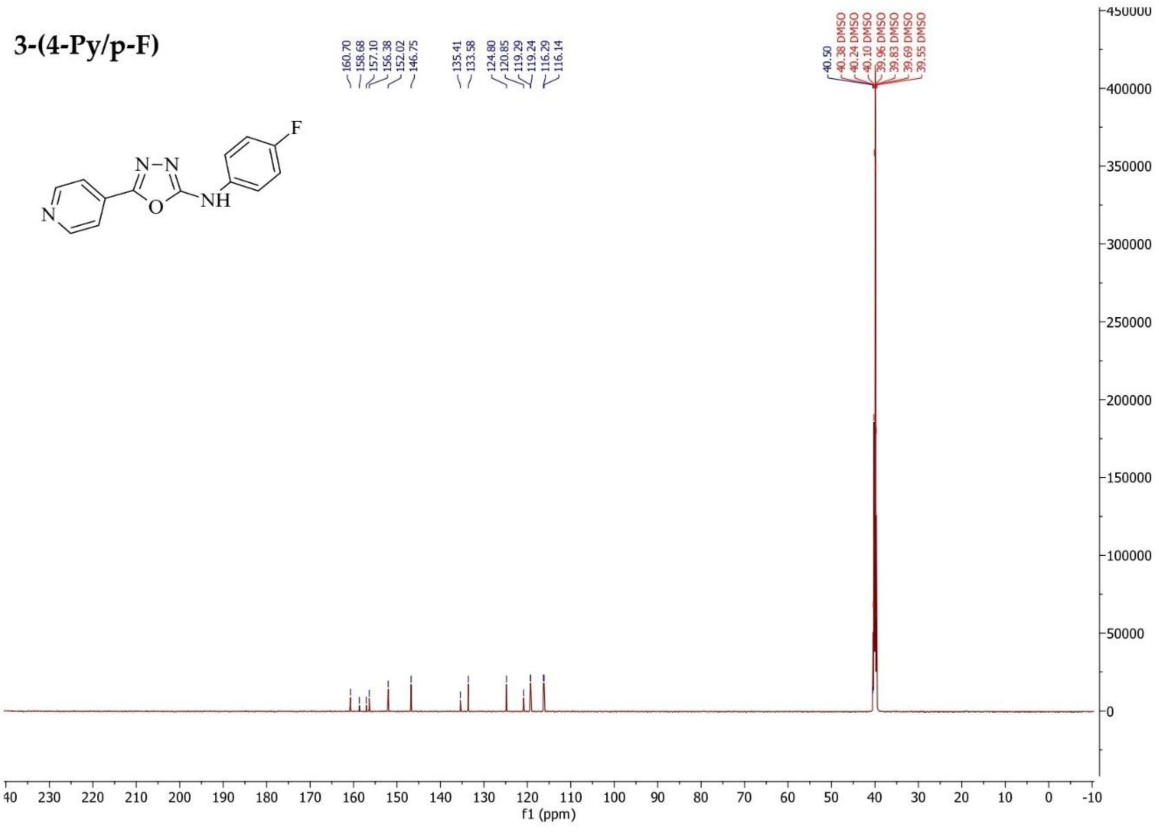

Figure S36. The ^{13}C NMR spectrum of compound **3-(4-Py/*p*-F)**

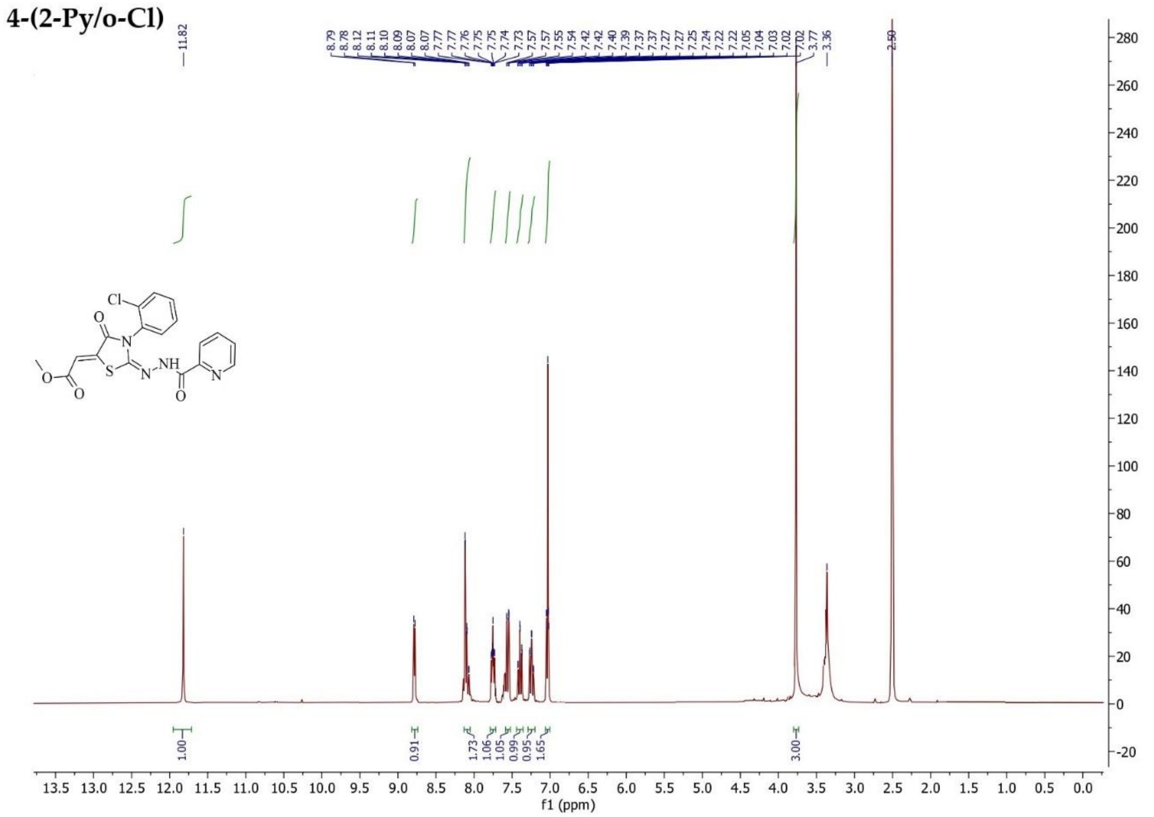

Figure S37. The ^{1}H NMR spectrum of compound **4-(2-Py/*o*-Cl)**

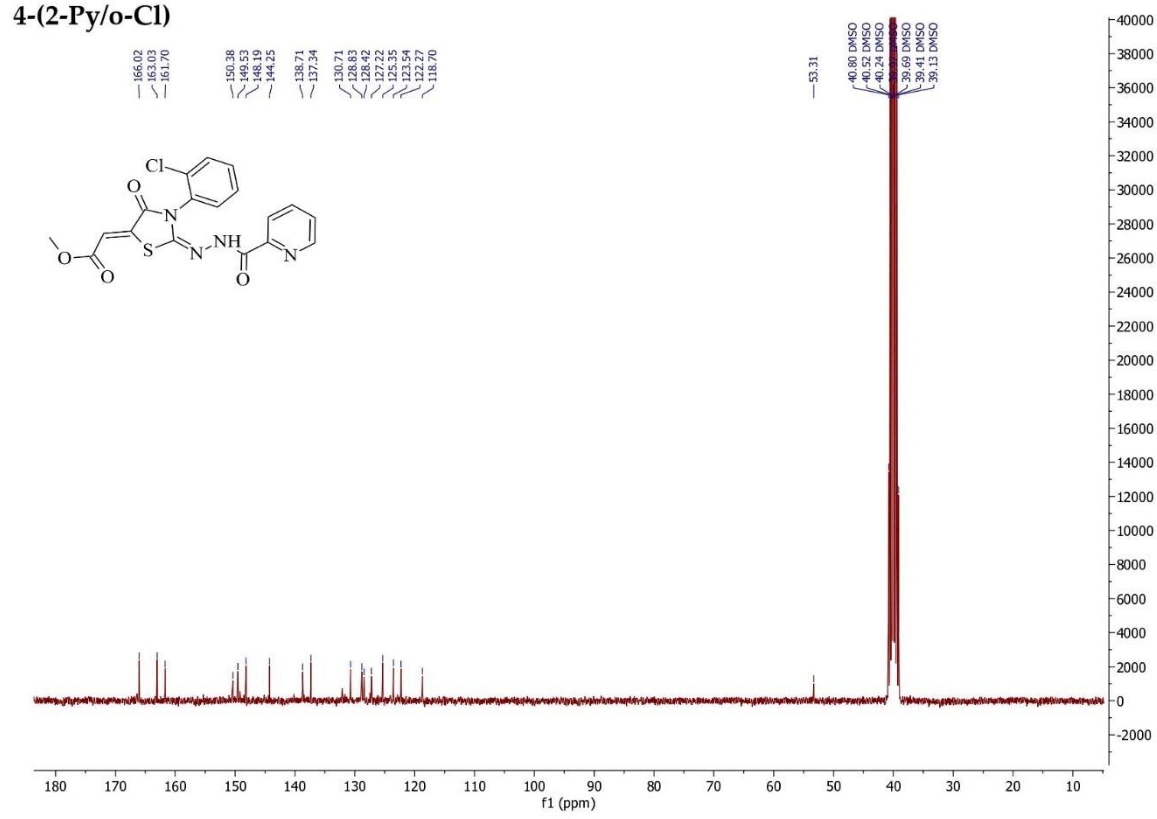

Figure S38. The ^{13}C NMR spectrum of compound **4-(2-Py/*o*-Cl)**

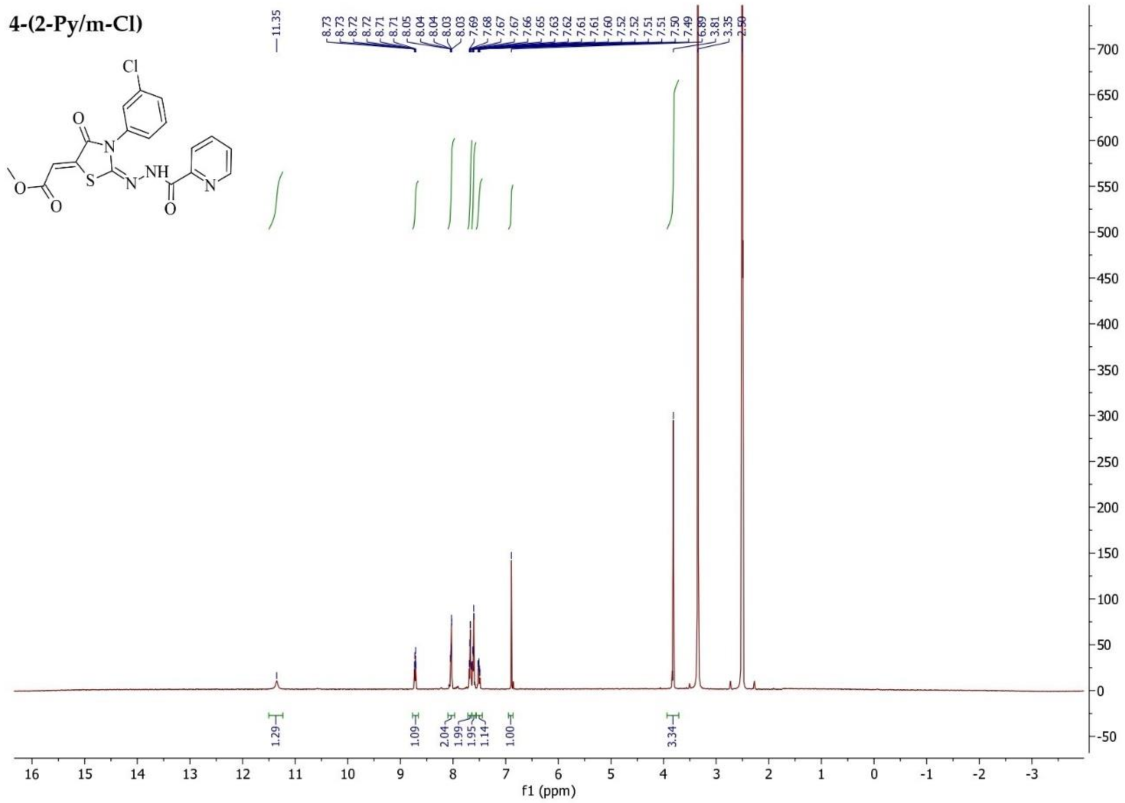

Figure S39. The ^{1}H NMR spectrum of compound **4-(2-Py/*m*-Cl)**

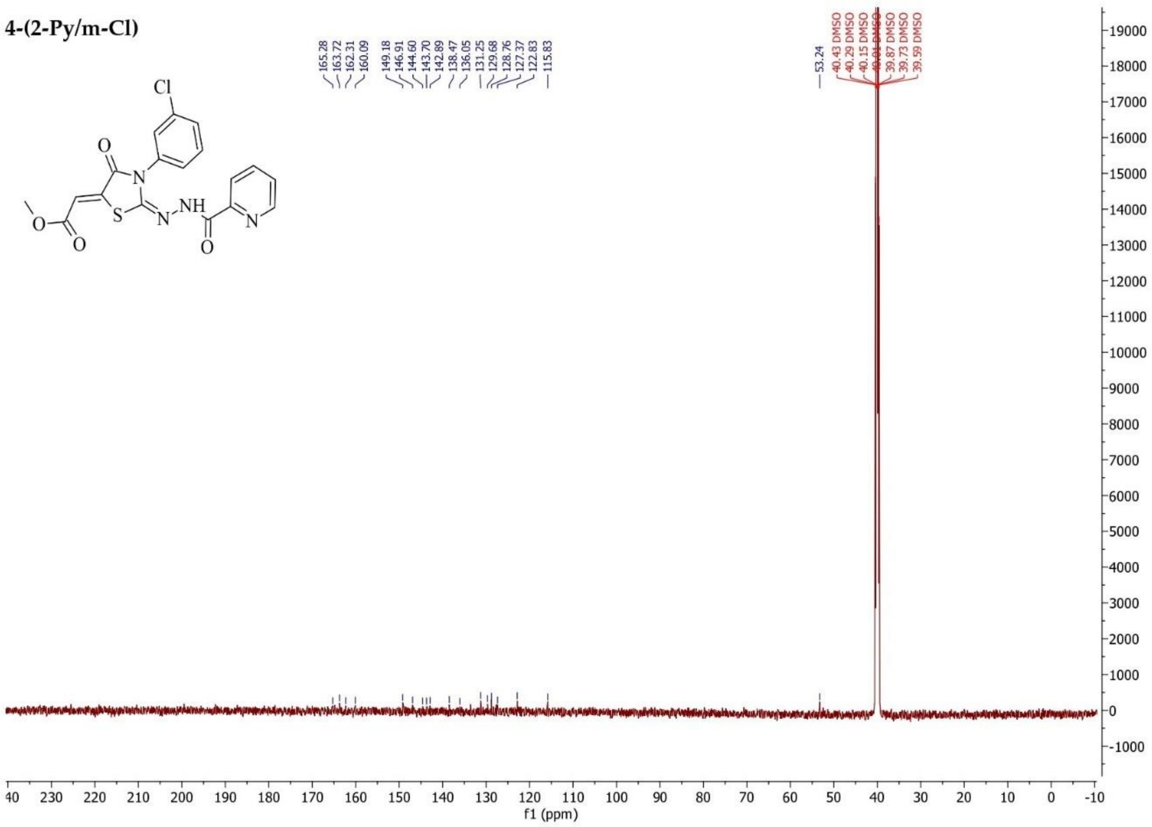

Figure S40. The ^{13}C NMR spectrum of compound **4-(2-Py/*m*-Cl)**

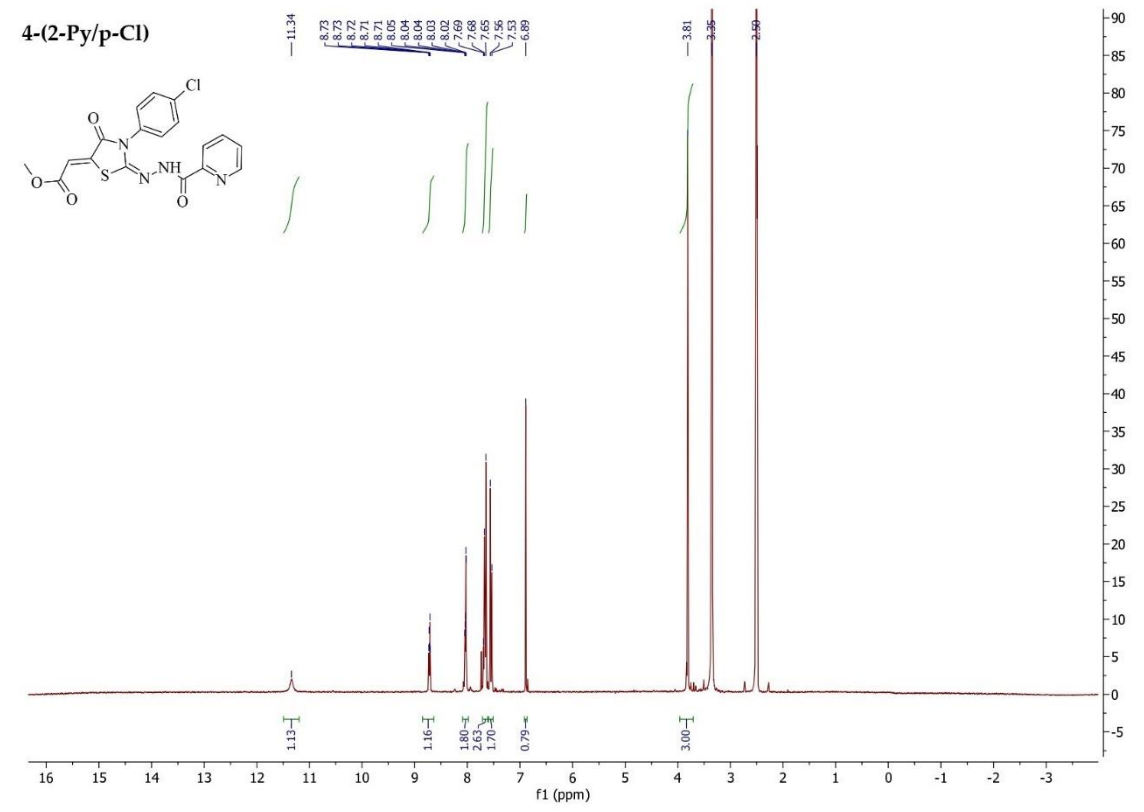

Figure S41. The ^{1}H NMR spectrum of compound **4-(2-Py/*p*-Cl)**

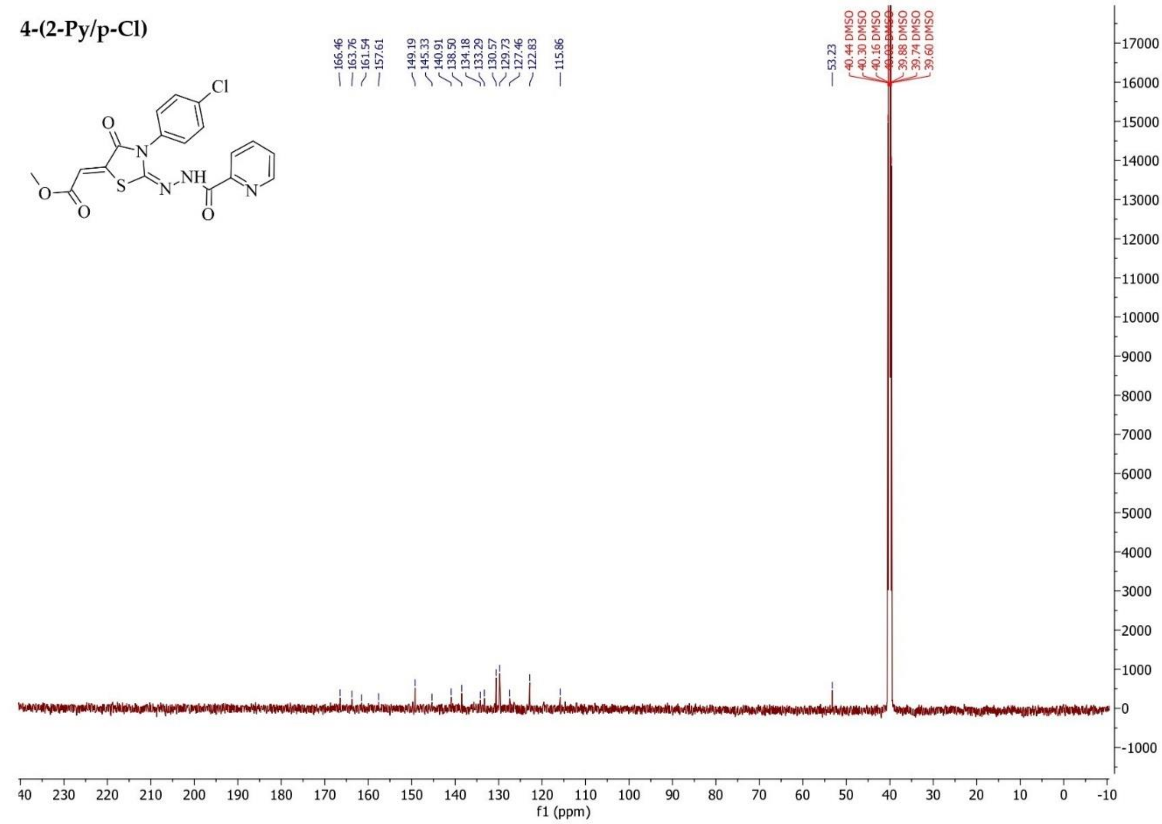

Figure S42. The ^{13}C NMR spectrum of compound **4-(2-Py/*p*-Cl)**

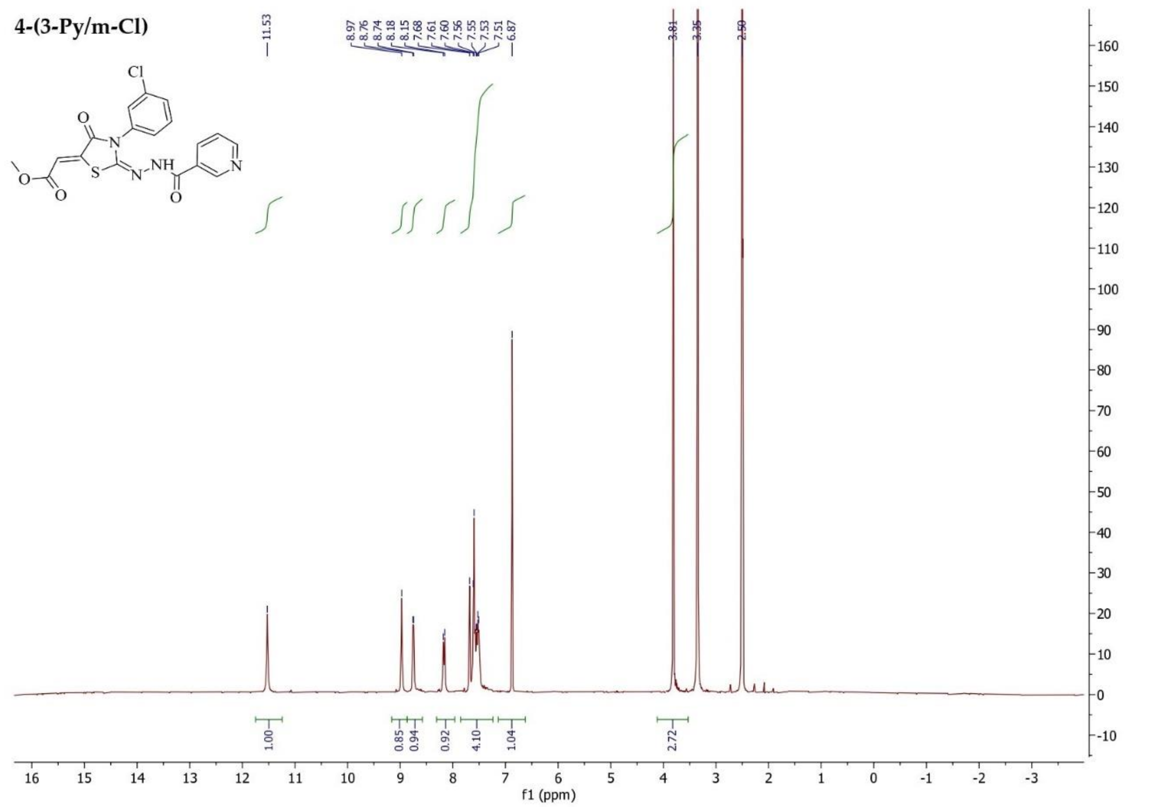

Figure S43. The ^{1}H NMR spectrum of compound **4-(3-Py/*m*-Cl)**

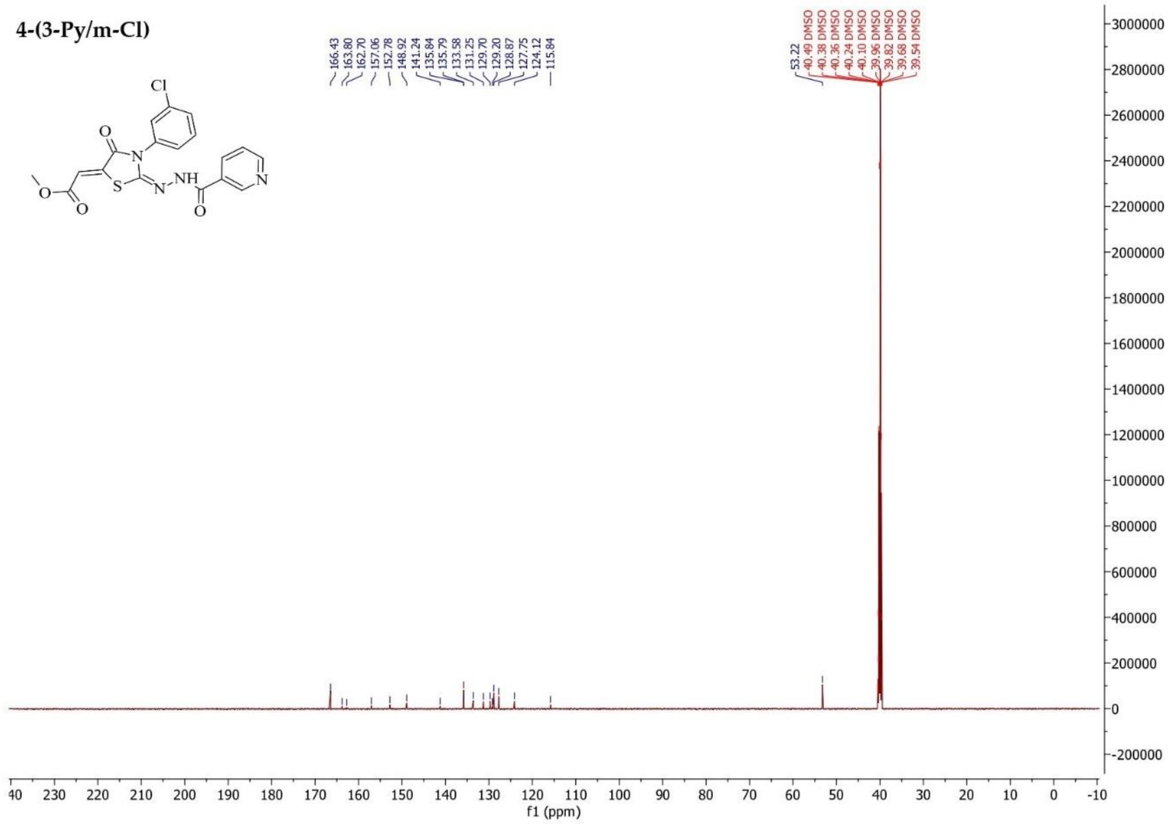

Figure S44. The ^{13}C NMR spectrum of compound **4-(3-Py/*m*-Cl)**

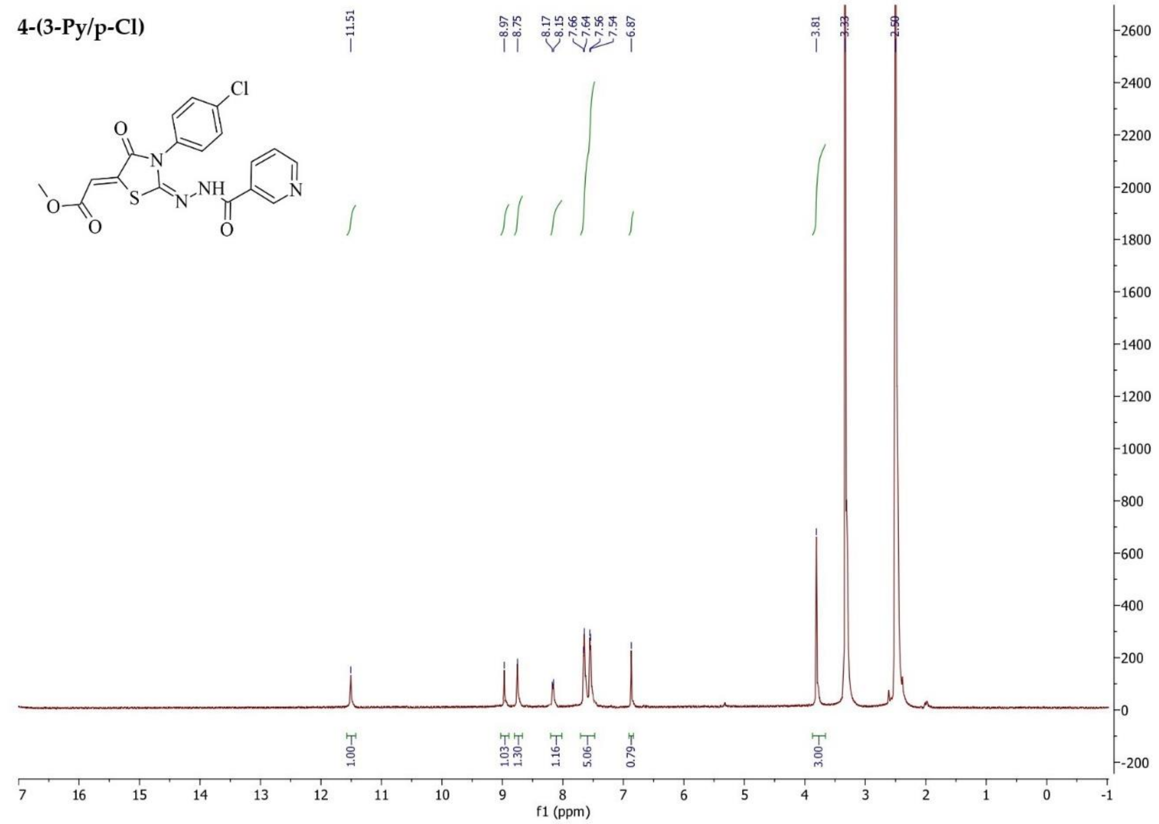

Figure S45. The ^{1}H NMR spectrum of compound **4-(3-Py/*p*-Cl)**

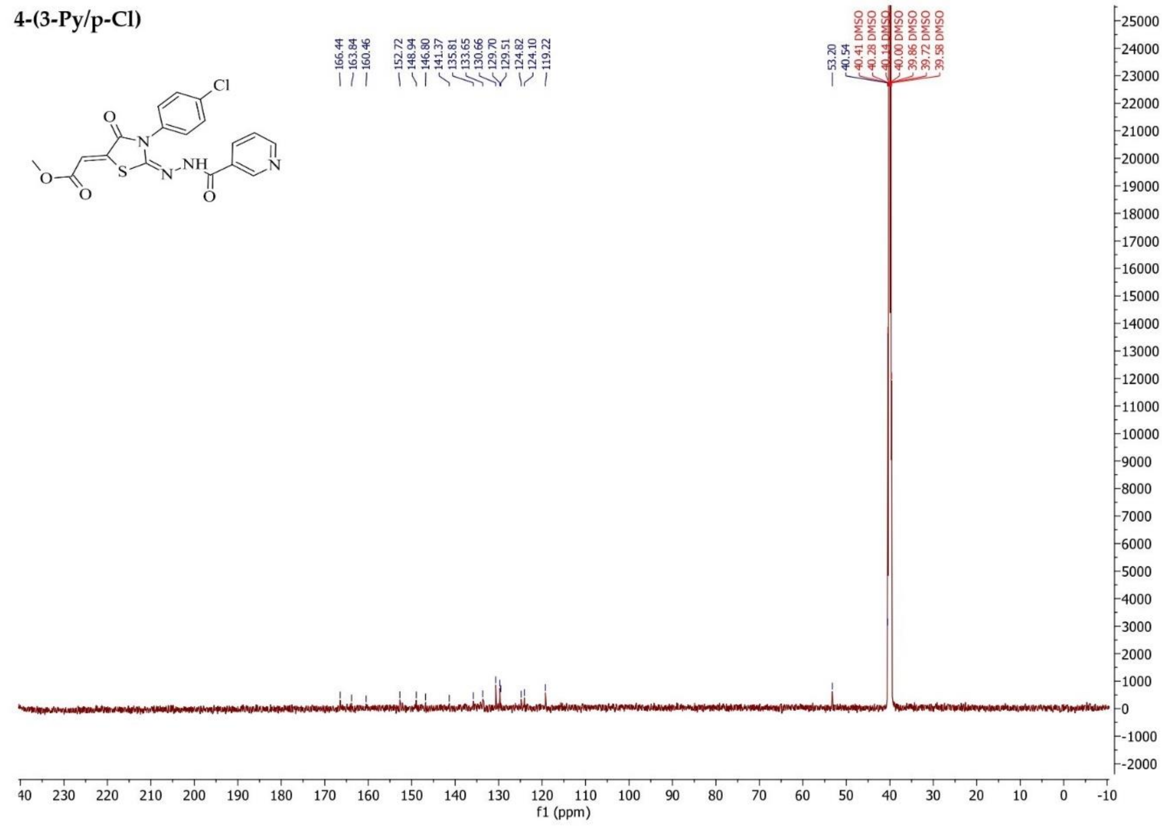

Figure S46. The ^{13}C NMR spectrum of compound **4-(3-Py/*p*-Cl)**

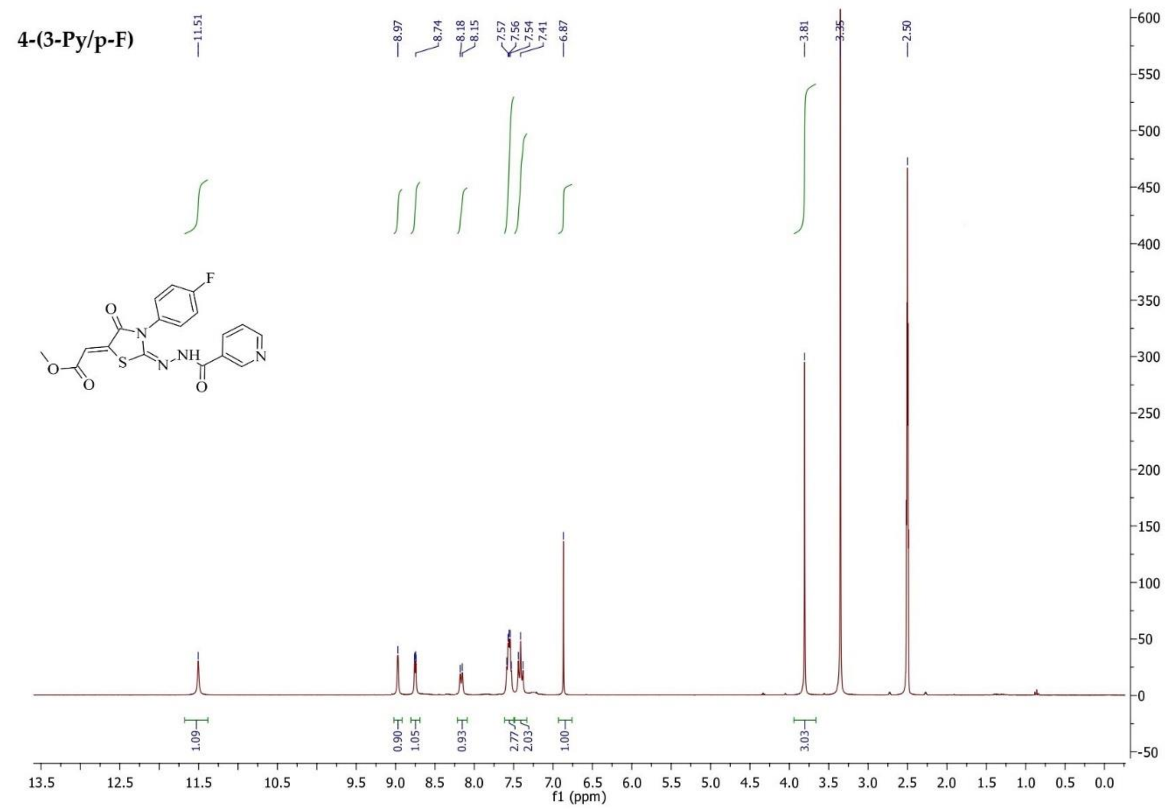

Figure S47. The ^{1}H NMR spectrum of compound **4-(3-Py/*p*-F)**

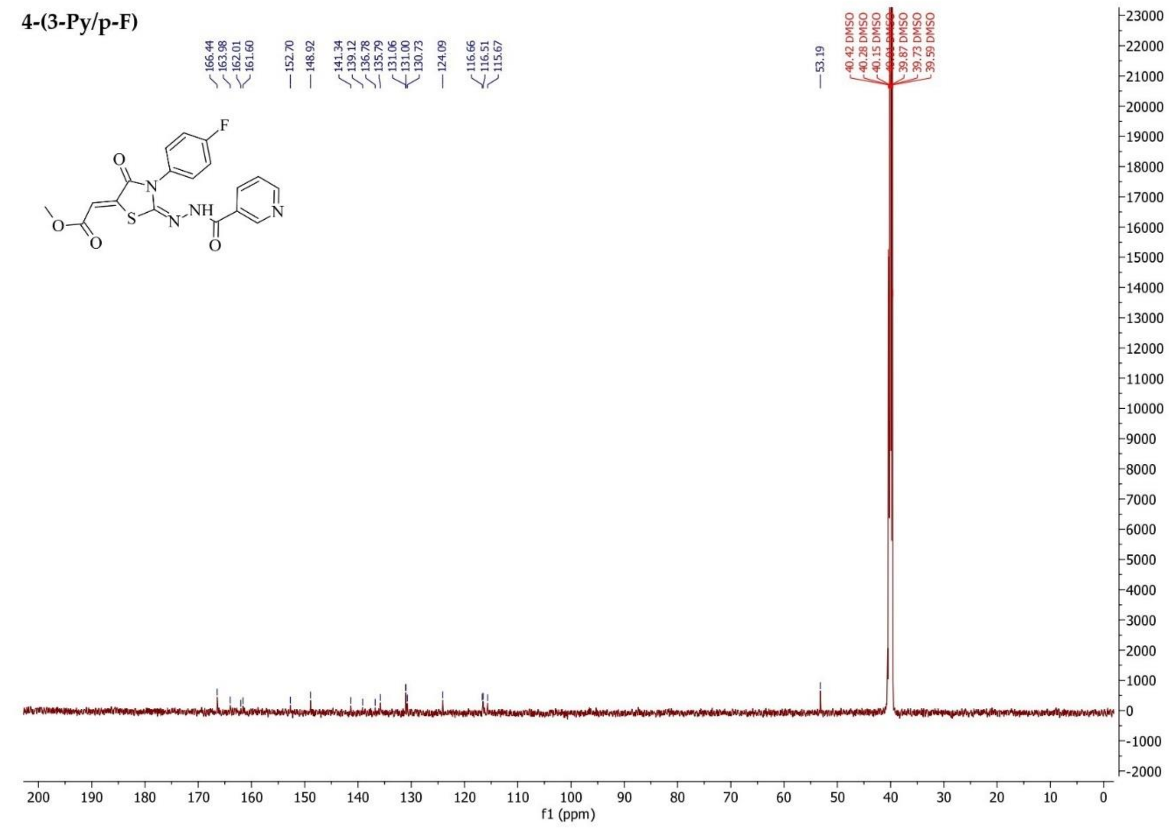

Figure S48. The ^{13}C NMR spectrum of compound **4-(3-Py/*p*-F)**

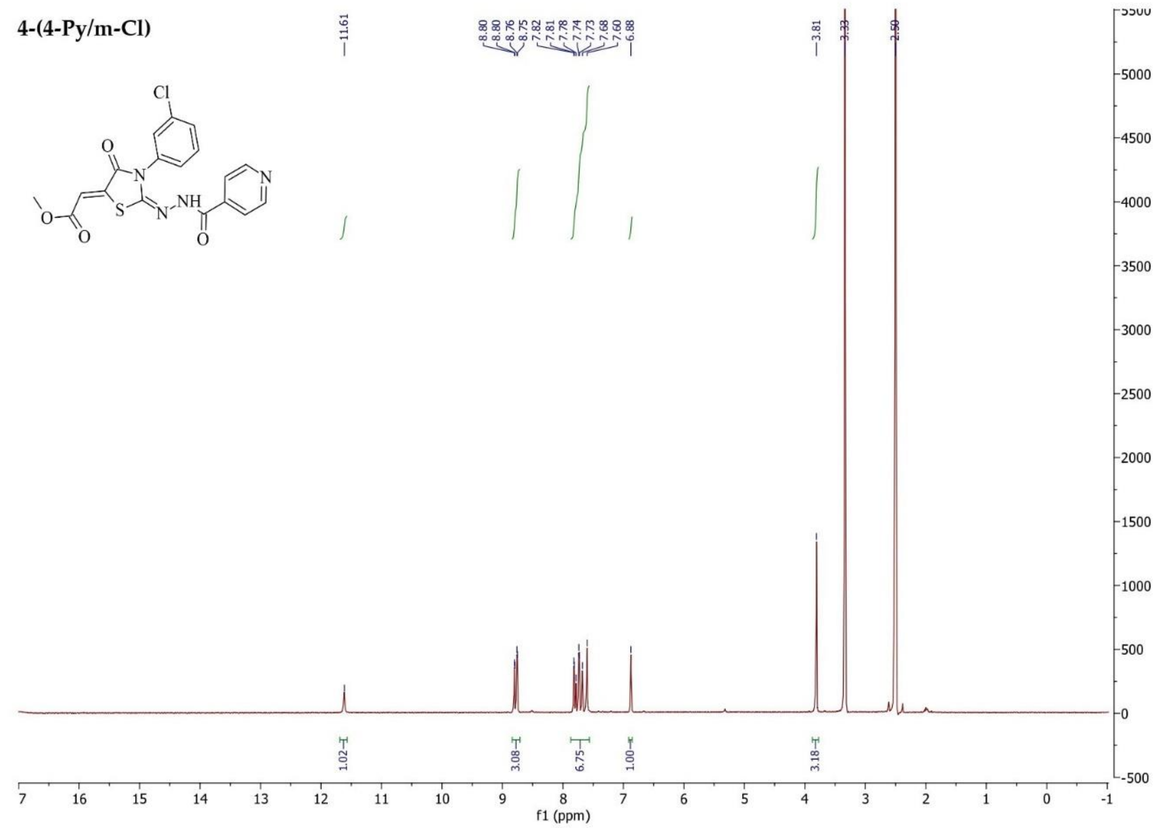

Figure S49. The ^{1}H NMR spectrum of compound **4-(4-Py/*m*-Cl)**

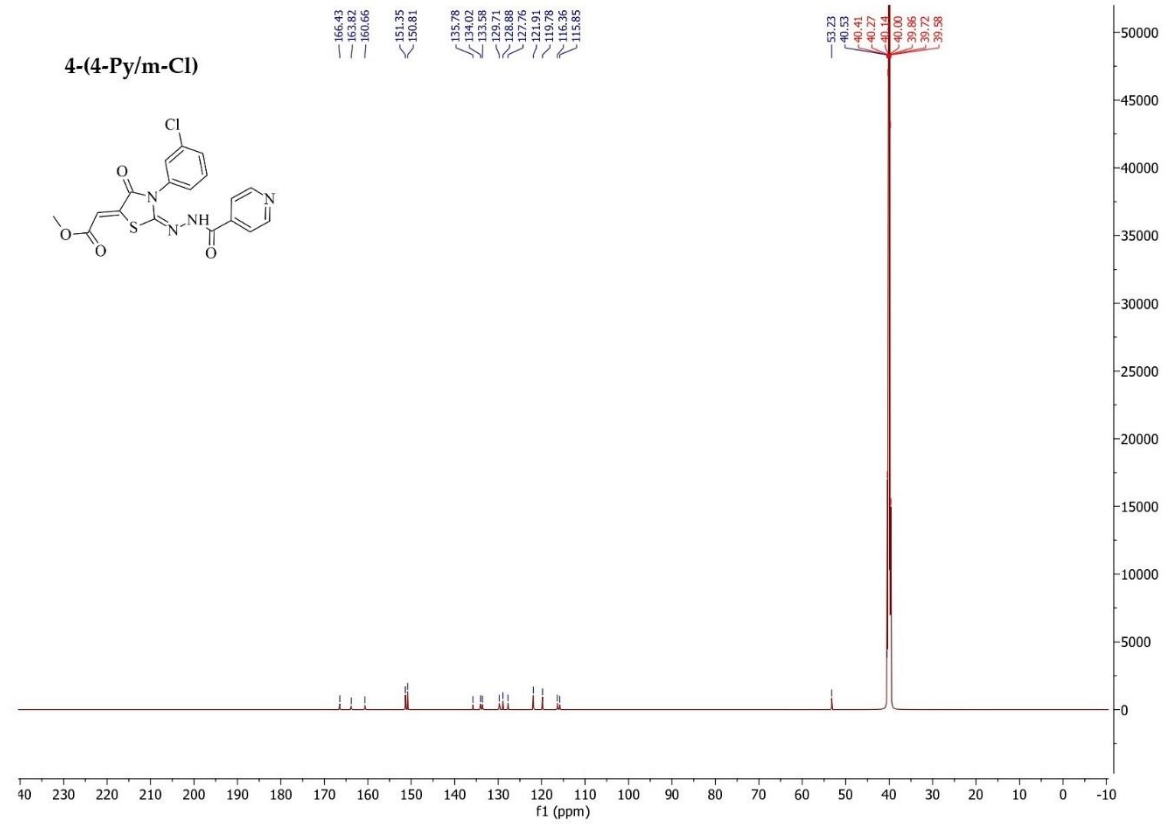

Figure S50. The ^{13}C NMR spectrum of compound **4-(4-Py/*m*-Cl)**

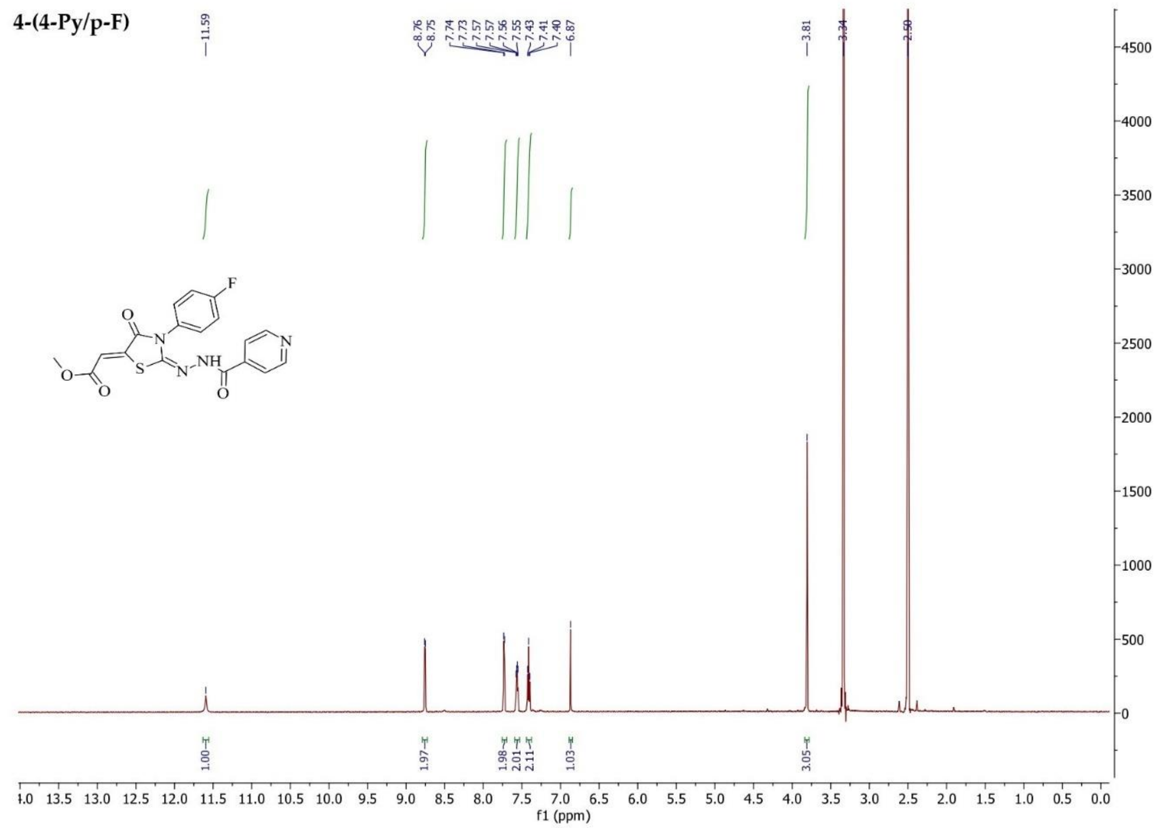

Figure S51. The ^{1}H NMR spectrum of compound **4-(4-Py/*p*-F)**

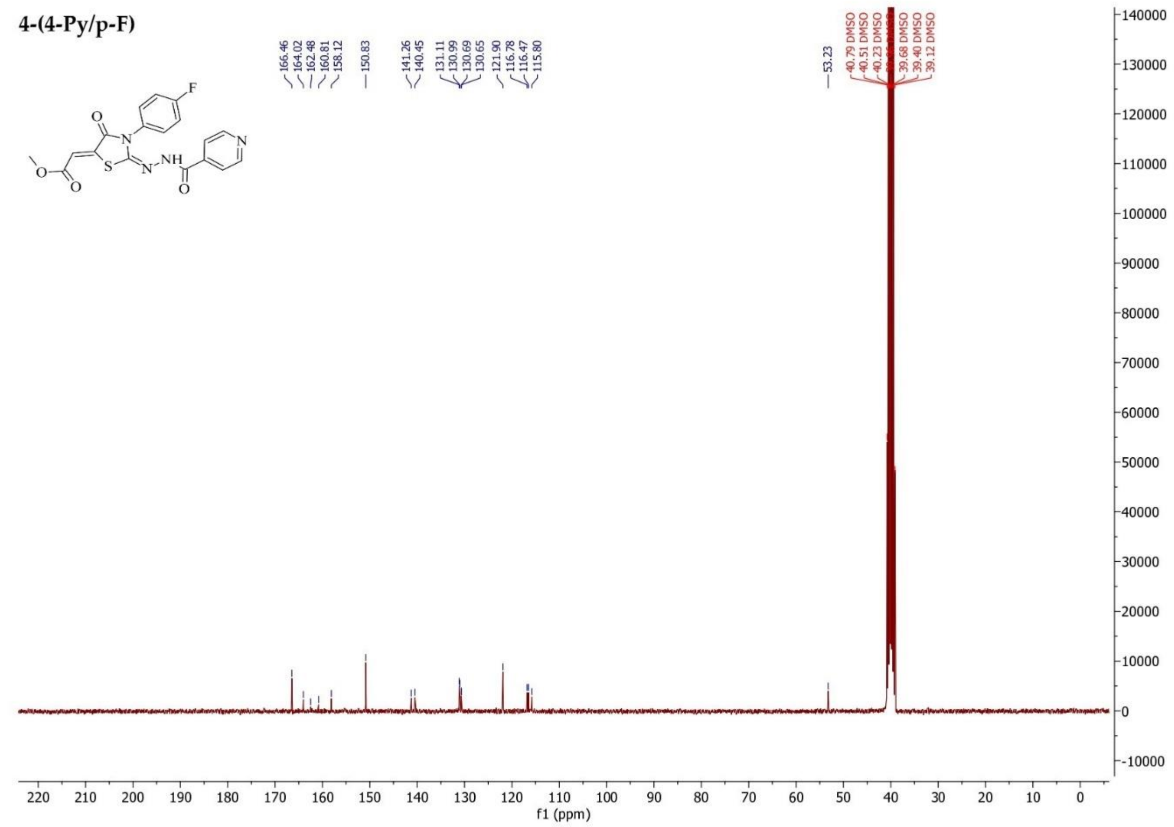

Figure S52. The ^{13}C NMR spectrum of compound **4-(4-Py/*p*-F)**

Table S1. Optimization of reaction condition and isolated yields of the target compounds.

Thiosemicarbazide precursor	Reagents and solvents	Reaction condition	Structure of cyclization products	Isolated yield (%)
**1-(2-Py/*p*-Cl)**	**$BrCH_2COOEt$, K_2CO_3, aceton**	**Stirred at rt for 2h**	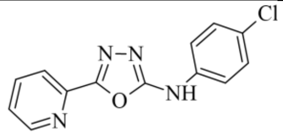	**0**
**1-(2-Py/*p*-Cl)**	**$BrCH_2COOEt$, NaOH, water**	**Stirred at rt for 2h**	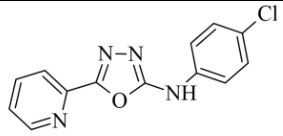	**0**
**1-(2-Py/*p*-Cl)**	**$BrCH_2COOEt$, K_2CO_3, aceton**	**Heat under reflux for 1.5h**	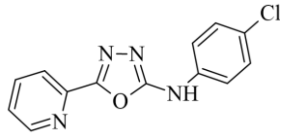	**21**
**1-(2-Py/*p*-Cl)**	**$BrCH_2COOEt$, K_2CO_3, DMF**	**Stirred at rt for 2h**	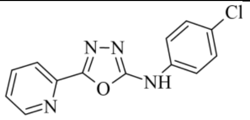	**0**
**1-(2-Py/*p*-Cl)**	**$BrCH_2COOEt$, K_2CO_3, DMF**	**Heat at 80°C for 2h**	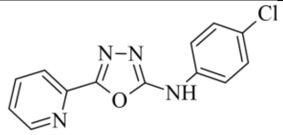	**18**
**1-(2-Py/*p*-Cl)**	**$BrCH_2COOEt$, NaOH, water**	**Heat under reflux for 1.5h**	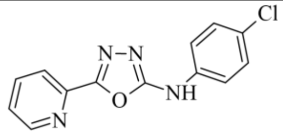	**0**
**1-(2-Py/*p*-Cl)**	**$BrCH_2COOEt$, AcONa, EtOH**	**Heat under reflux for 1.5h**	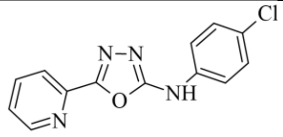	**62**
